# Structure of endogenous Pfs230:Pfs48/45 in complex with potent malaria transmission-blocking antibodies

**DOI:** 10.1101/2025.02.14.638310

**Published:** 2025-02-15

**Authors:** Ezra T. Bekkering, Randy Yoo, Sophia Hailemariam, Fabian Heide, Danton Ivanochko, Matthew Jackman, Nicholas I. Proellochs, Rianne Stoter, Oscar T. Wanders, Renate C. van Daalen, Maartje R. Inklaar, Carolina M. Andrade, Pascal W.T.C. Jansen, Michiel Vermeulen, Teun Bousema, John L. Rubinstein, Taco W.A. Kooij, Matthijs M. Jore, Jean-Philippe Julien

**Affiliations:** 1 Department of Medical Microbiology, Radboud University Medical Center, The Netherlands; 2 Program in Molecular Medicine, The Hospital for Sick Children Research Institute, Toronto, ON, Canada.; 3 Department of Biochemistry, University of Toronto, Toronto, ON, Canada.; 4 Department of Molecular Biology, Faculty of Science, Oncode Institute, Radboud University Nijmegen, Nijmegen, The Netherlands; 5 Division of Molecular Genetics, The Netherlands Cancer Institute, Amsterdam, The Netherlands; 6 Department of Medical Biophysics, University of Toronto, Toronto, Ontario, Canada; 7 Department of Immunology, University of Toronto, Toronto, Ontario, Canada

**Keywords:** Malaria, *Plasmodium falciparum*, Pfs230, Pfs48/45, cryo-electron microscopy, monoclonal antibodies, transmission-reducing activity, transmission-blocking vaccines

## Abstract

The Pfs230:Pfs48/45 complex is essential for malaria parasites to infect mosquitoes and forms the basis for current leading transmission-blocking vaccine candidates, yet little is known about its molecular assembly. Here, we used cryogenic electron microscopy to elucidate the structure of the endogenous Pfs230:Pfs48/45 complex bound to six potent transmission-blocking antibodies. Pfs230 consists of multiple domain clusters rigidified by interactions mediated through insertion domains. Membrane-anchored Pfs48/45 forms a disc-like structure and interacts with a short C-terminal peptide on Pfs230 that is critical for Pfs230 membrane-retention *in vivo*. Analyses of Pfs48/45- and Pfs230-targeted antibodies identify conserved epitopes on the Pfs230:Pfs48/45 complex and provides a structural paradigm for complement-dependent activity of Pfs230-targeting antibodies. Altogether, the Pfs230:Pfs48/45 antibody-complex structure presented improves our understanding of malaria transmission biology and the mechanisms of action of transmission-blocking antibodies, informing the development of next-generation transmission-blocking interventions.

## Introduction

Nearly half of the human population is at risk for malaria infection, making malaria one of the largest public health concerns (1). The parasite that is responsible for the majority of fatal human cases, *Plasmodium falciparum*, is efficiently transmitted by *Anopheles* mosquitoes throughout the population, with low parasite numbers being sufficient for mosquito infections (2) and studies reporting basic reproductive numbers R_0_ over 100 (3). Human-to-mosquito transmission is mediated by mature male and female gametocytes that are taken up by an *Anopheles* mosquito during a bloodmeal (4). Inside the mosquito midgut, male and female gametocytes rapidly activate and egress from their red blood cells (RBCs) as micro- and macrogametes, respectively. Microgametes fuse with macrogametes creating a zygote that forms the basis for further parasite development in the mosquito, eventually resulting in an infectious mosquito that can further spread the parasite and its related disease (5).

**Figure 1:**
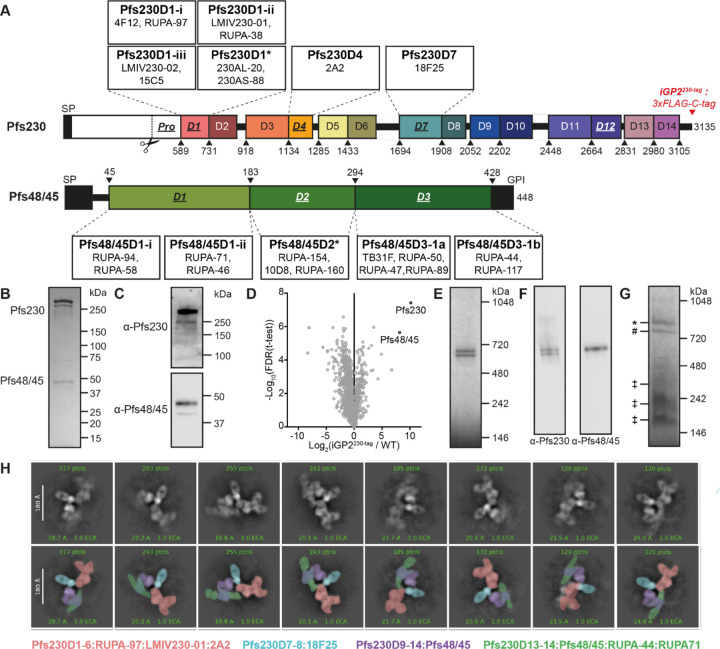
Purification of Pfs230:Pfs48/45:6Fab complex from *P. falciparum* gametocytes. (A) Schematic illustration of Pfs230 and Pfs48/45 domains (not to scale), epitopes found within these domains, and examples of mAbs targeting these epitopes (see also Table S1). Asterisks indicate multiple additional distinct epitopes at the indicated domain. Domains that contain known transmission-blocking epitopes are indicated by underlined italic. Arrows indicate amino acid number of the approximate start of the domain as determined by (15). SP = Signal peptide, GPI = Glycosylphosphatidylinositol anchor site. (B-C) Coomassie Blue (B) and immunoblot (C) staining of a denaturing SDS-PAGE gel of FLAG-tag purified Pfs230:Pfs48/45 complex. (D) Mass spectrometry analysis of FLAG-tag purified Pfs230:Pfs48/45 complex. The volcano plot shows the log_2_ fold change plotted against the log_10_ fold false discovery rate, comparing FLAG-tag co-immunoprecipitation of iGP2^230-tag^ versus NF54/WT parasites. (E-G) Blue-Native PAGE analysis, showing (E) Coomassie blue and (F) α-Pfs230 and α-Pfs48/45 immunoblot staining of neat purified Pfs230:Pfs48/45 complex, or (G) Coomassie blue staining of eluted complex incubated with molar excess of six Fabs (RUPA-97, LMIV230–01, 2A2, 18F25, RUPA-44, and RUPA-71). Symbols: * = Pfs230:Pfs48/45:6Fab ; # = Pfs230:4Fab ; ‡ = excess Fab fragment. (H) Eight representative 2D class averages generated from cryo-EM of HPLC-SEC purified Pfs230:Pfs48/45:6Fab complex. Structures are annotated in the lower row, with orange highlighting Pfs230D1–6:RUPA-97:LMIV230–01:2A2, blue Pfs230D7–8:18F25, purple Pfs230D-13–14:Pfs48/45, and green Pfs230D13–14:Pfs48/45:RUPA-44:RUPA-71. See also Figures S1-3 and Table S1.

Two sexual-stage surface proteins essential for *Plasmodium* transmission, Pfs230 (6) and Pfs48/45 (7), are expressed in both gametocytes and gametes. Pfs48/45 and Pfs230 knockout (KO) parasites showed markedly lower oocyst formation rates, demonstrating that these proteins play a critical role in establishing mosquito infection (8, 9). Based on rodent models, it has been hypothesized that these proteins are only essential for male fertility (9, 10), but this remains to be demonstrated for *P. falciparum*. Pfs48/45 is predicted to have a GPI-anchor and localizes together with Pfs230 to the parasite plasma membrane (PPM) (11). Pfs230, without a recognizable GPI anchor or transmembrane motif, is thought to associate with the PPM by forming a stable heterodimeric complex with Pfs48/45, as shown in co-immunoprecipitation studies (12, 13). This is further supported by the observation that Pfs48/45 KO parasites lose Pfs230 surface retention (8). Both proteins are part of the 6-Cysteine (6-Cys) protein family, characterized by the presence of 6-Cys domains: Immunoglobulin-like folds that contain up to six cysteines that can form disulfide bonds (14, 15). Pfs230 contains fourteen 6-Cys domains, whereas Pfs48/45 contains three (Figure 1A). Structural information on the full Pfs230:Pfs48/45 structure has been limited; two full-length structures of recombinant Pfs48/45 have been reported, adopting two distinct conformations – a disc-like (16) and an extended (17) conformation – whereas the structural characterization of Pfs230 has been limited to just the first two domains of the protein (18–22). Additionally, there is a lack of structural understanding of how Pfs230 and Pfs48/45 interact with one another.

The human-to-mosquito transmission stages form a developmental bottleneck for the malaria parasite and are therefore an attractive target for interventions seeking malaria elimination (23), such as transmission-blocking vaccines (TBVs). Indeed, modeling studies have suggested that TBVs could synergistically increase the effectiveness of vaccines targeting other parasite stages (24–26). Importantly, both Pfs230 and Pfs48/45 were recognized in the early 1980s as the targets for antibodies that had potent human-to-mosquito transmission-reducing activity (TRA) (11, 13, 27). Since then, multiple studies have shown that naturally infected individuals can acquire Pfs230 and Pfs48/45-targeted antibodies that are associated with high-level TRA in serum (*e.g.* (28–33)) and these antibodies can block transmission when purified (28). The structure, epitopes, and TRA of these naturally acquired antibodies have been partially characterized at the monoclonal antibody level (17, 22, 34). Domain 1 of Pfs230 (Pfs230D1) and domain 3 of Pfs48/45 (Pfs48/45D3) are the target of the most potent monoclonal antibodies (mAbs) with TRA that can potently block human-to-mosquito transmission (*e.g.* (17, 19, 21, 22, 34–37)). Initial reports suggested that other domains of Pfs230 and Pfs48/45 were not able to elicit comparably potent transmission-blocking antibodies (37). Recently at least three other Pfs230 domains, and Pfs48/45D1, have been identified as targets for potent transmission-blocking antibodies, but structural insight into these epitopes is lacking in comparison to Pfs230D1 and Pfs48/45D3 (34, 38–41).

Regardless of the domain they target, the vast majority of the potent α-Pfs230 antibodies is complement-dependent (19, 22, 38, 42), although some antibodies retain TRA at very high concentrations in the absence of complement (18, 36). In contrast, α-Pfs48/45 antibodies can be neutralizing at low concentrations in a complement-independent manner (17, 20, 34, 35, 40, 43). The rationale behind what drives antibody potency for both α-Pfs230 and α-Pfs48/45 antibodies remains unclear. As both proteins are capable of eliciting highly potent antibodies, they have been targeted as lead antigens and are currently the most advanced and promising TBVs in development (44, 45). Indeed, immunogens based off these two proteins have been extensively evaluated in preclinical and phase I/II clinical trials (46–49). However, these vaccines did not block transmission completely and it remains unclear whether they can induce long-lasting immunity. Structure-function relationships of how potent antibodies target the Pfs230:Pfs48 complex would not only enhance our understanding of *P. falciparum* transmission biology and antibody mechanism of actions, but also aid in the design and development of next-generation malaria vaccines more fully capable of blocking transmission to reach elimination goals.

Here, we report the molecular structure of the endogenous Pfs230:Pfs48/45 heterodimer in complex with six antibodies with potent TRA as solved by cryogenic electron microscopy (cryo-EM). Cumulatively, our data provide critical molecular insights into the Pfs230:Pfs48/45 heterodimer complex, moving towards deepening our understanding of *P. falciparum* transmission biology and opportunities to block this process through next-generation biomedical interventions.

## Results

### Elucidation of the endogenous Pfs230:Pfs48/45 complex structure

We set out to determine the cryo-EM structure of the Pfs230:Pfs48/45 complex by purifying it from mature *P. falciparum* gametocytes. Using the inducible gametocyte producing parasite line NF54/iGP2 (50), we fused *pfs230* (Pf3D7_0209000) C-terminally with a 3xFLAG-C-tag coding sequence, yielding parasite line iGP2^230-tag^ (Figure S1A-D). iGP2^230-tag^ gametocytes showed normal Pfs230 and Pfs48/45 localization and retained normal *in vitro* exflagellation and mosquito infectivity in standard membrane feeding assays (SMFA; Figure S1E-F). The Pfs230:Pfs48/45 complex was purified using anti-FLAG resin. Pfs230 and Pfs48/45 were the highest enriched proteins from this purification as detected by LC-MS/MS (Figure 1B-D). Two high-molecular weight bands were observed in Blue-Native Polyacrylamide Gel Electrophoresis (BN-PAGE). Both bands were stained by α-Pfs230 antibodies in immunoblotting assays, but only one band was stained by α-Pfs48/45 antibodies, attributing these two bands to the Pfs230:Pfs48/45 complex and Pfs230 alone, respectively (Figure 1E-F). BN-PAGE analysis of eluted protein incubated with a molar excess of different fragment of antigen-binding (Fab) fragments of transmission-blocking antibodies, targeting a range of conformational epitopes on Pfs48/45 and Pfs230, confirmed the native structure of the eluted proteins (Figure S1G). While α-Pfs230 Fabs shifted both protein species upwards, α-Pfs48/45 Fabs only affected the upper band, further ascertaining the top band as the Pfs230:Pfs48/45 complex.

We posited that Fabs could be used as fiducial markers to help distinguish in a cryo-EM structure the seventeen highly similar 6-Cys domains found in the Pfs230:Pfs48/45 complex. As such, we obtained a size exclusion chromatography (SEC)-purified Pfs230:Pfs48/45 heterodimer in complex with six Fab fragments, using the structurally delineated RUPA-97 (22), LMIV230–01 (21), and RUPA-44 (34), and the yet-to-be-structurally delineated 2A2 (40, 42, 51), 18F25 (38, 51), and RUPA-71 (17, 34) inhibitory antibodies (Figure 1G, Figure S1H). Cryo-EM data of this Pfs230:Pfs48/45:6Fab complex was collected at 0°, 35°, and 40° tilt. 2D class averages from this dataset clearly revealed the presence of all components of the complex: Pfs230 bound to four Fabs (LMIV230–01, RUPA-97, 2A2, 18F25) in complex with Pfs48/45 bound to two Fabs (RUPA-44 and RUPA-71) (Figure 1H).

The highest resolution structural information was derived by generating four locally refined maps corresponding to more rigid components of the complex: Pfs230D1–6 bound to RUPA-97, LMIV230–01, and 2A2 at a global resolution of 3.6 Å; Pfs230D7–8 bound to 18F25 at a global resolution of 3.8 Å; Pfs230D9–14 bound to Pfs48/45 at a global resolution of 4.7 Å; and Pfs230D13–14 bound to Pfs48/45, RUPA-44, and RUPA-71 at a global resolution of 3.5 Å (Figure 1H, Figures S2-3, Table 1).

### Insertion domains are responsible for Pfs230 domain organization

**Figure 2:**
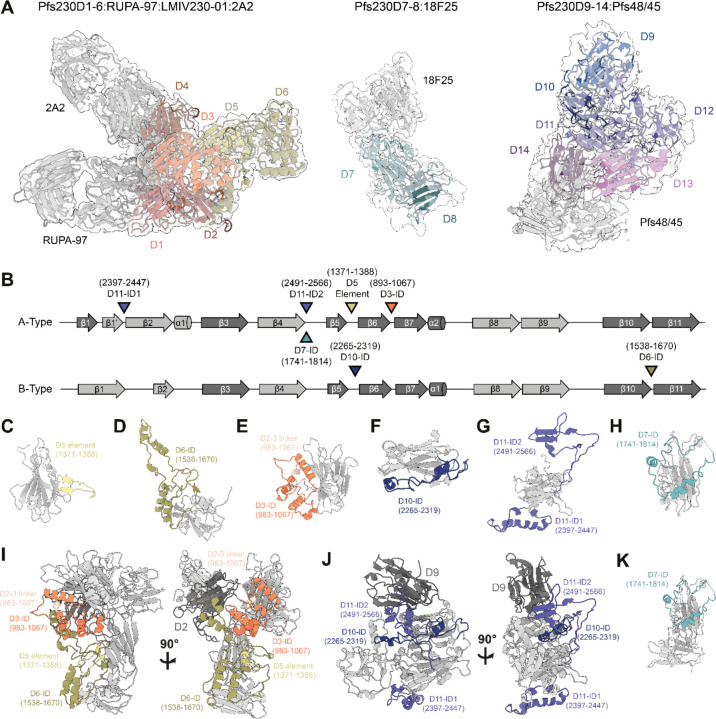
Structural Basis of Pfs230 6-Cys Domain Clustering. (A) Models of Pfs230D1–6:RUPA-97:LMIV230–01:2A2, PfsD7–8:18F25, and Pfs230D9–14:Pfs4845 shown in their respective maps (rendered at 4.5 Å radius around the models). Domains are coloured as in Figure 1. (B) Secondary structural elements of A- and B-type domains, with insertion domains (ID) or non-canonical structural element (D5 element) found in respective domains indicated by coloured arrows and with residue range in brackets. (C) D5 structural element, (D) Pfs230D6-ID, (E) D2-D3 linker (light orange) and D3-ID, (F) D10-ID, (G) D11-ID1 and –ID2, (H) and D7-ID are coloured as done previously with the rest of the domain coloured in silver. (I) The Pfs230D2-D3 linker (light orange), D3-ID (orange), D5 structural element (yellow), and D6-ID (dark yellow) interact with one another forming a structural fold that packs against the base of Pfs230D2 (dark grey). (J) The Pfs230D10-ID (blue) and Pfs230D11-ID2 (purple) interact with one another, positioning the D11-ID2 to form an extended β-sheet with D9 (dark grey). (K) Pfs230D7–8 (silver) and Pfs2230D7-ID (cyan) lack interdomain contact. See also Figure S4.

Molecular models were built into each of the locally refined maps to elucidate structural features of the interdomain topologies and dispositions of potent mAbs. The models derived from the maps of Pfs230D1–6:RUPA-97:LMIV230–01:2A2 and Pfs230D7–8:18F25 (Figure 2A, Table 1) were initialized from experimentally determined crystal structures and predicted AlphaFold (AF) models, which were refined into maps with sufficiently high resolution to model sidechain positions (Table 1, Table S3). For the 4.7 Å resolution map of Pfs230D9–14:Pfs48/45, we report the backbone positions of a compositely refined Pfs230D9–12 structure in the presence of the Pfs230D13–14:Pfs48/45 structure decerned from the 3.5 Å resolution map (Figure 2A, Table 1). AF models of Pfs230D9-D12 were initially docked and manually rearranged into the Pfs230D9–14:Pfs48/45 map. A template-guided inference strategy in AF2 followed by refinement using map-constrained energy minimizations resulted in the final model. We attribute the lower local resolution of Pfs230D9–12 in our Pfs48/45:Pfs230D9–14 map to intrinsic flexibility between the D12 and D13 domains (Figure S3).

Each tandem structural unit of Pfs230 consists of A- and B-type 6-Cys domains (Figure S4A). Most A-type domains feature a 5-on-5 β-sandwich whereas B-type domains feature a 4-on-5 β-sandwich fold (Figure S4B). A key differentiating feature of these folds is the way in which the first two β-strands are arranged. In the A-type folds (i.e. Pfs230 D1, D3, D5, D7, D9, D11, and D13), the first canonical β1 strand is split between the two β-sheets (β1 and β1’) of the 6-Cys β-sandwich fold before transitioning into the β2 strand. In contrast, in the B-type folds (i.e. Pfs230 D2, D4, D6, D8, D10, D12 and D14), the first two β-strands (β1 and β2) are part of the same β-sheet (Figure S4B).

While small deviations from the canonical 6-Cys domains exist, primarily near the perpendicular β-hairpin (Figure 2B-C, Figure S4C-D), most of the structural diversity that distinguishes the different Pfs230 6-Cys domains lie in long sequence insertions in loop regions between the β-strands. These regions were previously coined as insertion domains (IDs) (52, 53) and are present in Pfs230D3, D6, D7, D10 and D11 (Figure 2B). Many of these IDs (i.e. Pfs230D3, D6, D10, and D11-ID2) contribute to interdomain contacts (Figure 2C-G), while others (i.e. Pfs230D11-ID1 and D7) were not observed to interact with other domains (Figure 2G-H). The presence of these IDs mediates the formation of domain clusters in Pfs230 (Figure 2I-J).

The Pfs230D6-ID is the most extensive insertion domain (Figure 2D) (res. 1538–1670) and forms structural elements that interface with a β-hairpin in Pfs230D5 (res. 1371–1388) and interacts with the Pfs230D3-ID (res. 983–1067) and the Pfs230D2–3 linker (res. 983–1067) (Figure 2I). A portion of this structural fold localizes near Pfs230D3 and these elements altogether pack against the base of Pfs230D2, holding the Pfs230D1-D6 domain cluster together. Similarly, the Pfs230D10-ID (res. 2265–2319) localizes to the same region as Pfs230D11-ID2 (res. 2491–2566) near Pfs230D9. Here, the Pfs230D11-ID2 forms a β-hairpin that interacts with a β-sheet of Pfs230D9 – effectively forming an extended β-sheet consisting of two different domains (Figure 2J). The resolution of the map from which this part of the model was derived is low (4.7 Å), especially in the Pfs230 section of the map, making the modeling of Pfs230D11-ID2 more challenging (Figure S3D). Nonetheless, the portion of the ID that interacts with Pfs230D9 (res: 2514–2525) is consistently predicted to interact with Pfs230D9 across multiple AF runs and density accounting for this region is present in our map with a local resolution of approximately 6–7 Å (Figure S4E). This in conjunction with the integrative modeling approach utilized provides confidence in the backbone positions of these interactions. In summary, Pfs230 consists of four domain clusters (e.g. Pfs230D1–6, D7–8, D9–12, and D13–14) that are coordinated closely by IDs, with evident flexibility between the four domain clusters.

### Pfs230 interacts with Pfs48/45 through its C-terminus

**Figure 3:**
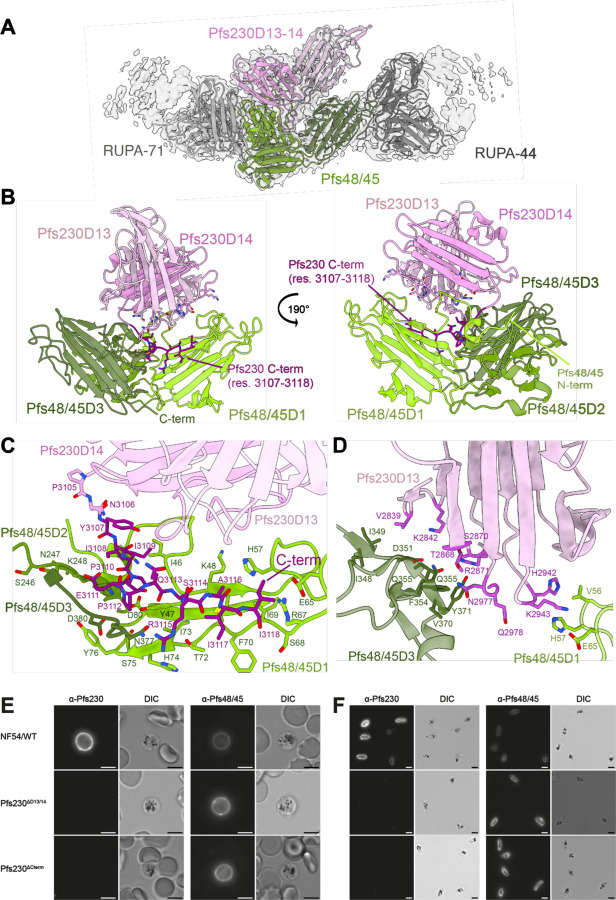
Molecular description of the Pfs230 and Pfs48/45 interaction. (A) Overview of the structure of Pfs48/45 (yellow green, olive drab, and dark olive drab for domains 1–3, respectively) bound to Pfs230D13–14 (plum), RUPA-71 Fab (dark grey), and RUPA-44 Fab (light grey) with the cryo-EM map shown in grey. (B) Pfs48/45 bound to Pfs230D13–14 with key Pfs230 contact residues shown as sticks and the C-terminal region of Pfs230 highlighted in dark magenta. (C-D) binding interactions between Pfs48/45 and Pfs230D14-Cterm (C) and Pfs230D13 (D) with key residue contacts shown as sticks. (E-F) Representative suspension immunofluorescence microscopy images of NF54/WT, 230^ΔD13/14^, and 230^ΔCterm^ female macrogametes (E) and saponin-treated stage V gametocytes (F). Parasites were incubated with 15 μg/ml 18F25-DyLight488 (α-Pfs230) or 45.1-DyLight488 (α-Pfs48/45). All images per experiment were taken and analyzed with the same settings. Scale bar represents 5 μm. See also Figure S5-6, Table S2.

The interaction site between Pfs48/45 and Pfs230 was delineated at 3.4 Å resolution (Figure 3A, Figure S3C, Table 1). Our structure indicates that Pfs230 engages with all three domains of Pfs48/45 through its terminal 6-Cys domains, Pfs230D13 (296 Å^2^) and Pfs230D14 (1,376 Å^2^) (Figure 3B). Pfs48/45 adopts a disc-like conformation in our structure with Pfs230, with Pfs48/45D1 (1057 Å^2^) and Pfs48/45D3 (478 Å^2^) accounting for the majority of contacts with Pfs230, while Pfs48/45D2 (112 Å^2^) contributes more modestly (Figure 3B, Figure S5A, Table S2). Pfs230 binds to Pfs48/45 on the opposite face of the disc conformation compared to the C-terminus of Pfs48/45, where the GPI linker is located for attachment to the PPM (Figure 3B). Previously, two different conformations of Pfs48/45 have been observed in the absence of Pfs230 binding, a compact, disc-like structure solved using X-ray crystallography (16) and an elongated Pfs48/45 structure determined by cryo-EM (17). A comparison of these models with our Pfs230-bound Pfs48/45 structure indicates very similar arrangements of Pfs48/45D1–2 (RSMD = 0.92–1.08 Å) (Figure S5B). However, the position of Pfs48/45D3 relative to Pfs48/45D1–2 varies considerably more. There is almost no overlap between Pfs48/45D3 from Pfs230-bound Pfs48/45 and the elongated Pfs48/45 structure while modest positional differences are observed between Pfs48/45D3 of the two disc-like Pfs48/45 structures (RMSD = 4.12 Å; Figure S5B).

The C-terminus of Pfs230 (Pfs230Cterm; res. 3108–3118) – a sequence extending out of the canonical 6-Cys domain – is integral for mediating the Pfs230:Pfs48/45 interaction (Figure 3C). These residues occupy a groove of the Pfs48/45 disc and form extensive interactions with loop 45–49 and strand 65–76 of Pfs48/45D1, loop 246–250 of Pfs48/45D2, and loop 377–380 of Pfs48/45D3 (Figure 3B). While accounting for fewer interactions than Pfs230D14, Pfs230D13 residues 2866–2871 and the intervening loop between Pfs230D13 and Pfs230D14 (res. 2977–2984) support complexation by engaging with a portion of a Pfs48/45D3 extended loop (res. 348–371) (Figure 3D). Additionally, the Pfs48/45 N-terminus wraps around Pfs48/45D1–2 and through residues 41–50 forms interactions with loops 2993–2998 and 3108–3114 and strand 3007–3014 of Pfs230D14 (Figure 3B). While the N-terminal region of Pfs48/45 remained largely unresolved in previous structural studies of Pfs48/45 without Pfs230 implying flexibility (16, 17), this region is well-resolved in the Pfs230-bound structure and reveals that unlike other A-type domains, Pfs48/45D1 features a different β1 orientation and connectivity (Figure S5C), localizing to the interface. An analysis of single nucleotide polymorphisms (SNP) across *P. falciparum* revealed that the interface between Pfs48/45 and Pfs230 is highly conserved (Figure S5A and S5D).

Corroborating these structural findings, a recombinantly produced protein construct spanning Pfs230D13–14-Cterm bound to female gametes on which Pfs48/45 is anchored, while similarly produced constructs spanning either Pfs230D1 or Pfs230D10 could not (Figure S6A). To further confirm the interaction between Pfs230D13–14-Cterm and Pfs48/45, two transgenic parasite lines carrying a truncated *pfs230* gene were created (Figure S6B-E). Pfs230^ΔD13/14^ parasites express Pfs230 up until Pfs230D12, while Pfs230 expressed in Pfs230^ΔCterm^ gametocytes only lacks the last 29 C-terminal residues (res. 3107 – 3135) seen as central to the Pfs230:Pfs48/45 interface in our cryo-EM structure. Wildtype Pfs230 is expected to localize on the outside of the PPM, facing into the parasitophorous vacuole, and is expected to co-localize with PPM-bound Pfs48/45. Western blot analysis shows that late-stage Pfs230^ΔD13/14^ and Pfs230^ΔCterm^ gametocytes still contain (truncated) Pfs230. However, saponin-treated parasites with a disrupted parasitophorous vacuole membrane (PVM) lose all Pfs230, demonstrating that Pfs230 is no longer retained on the PPM (Figure S6F-G). In fixed immunofluorescence microscopy, full-length and truncated Pfs230 proteins localized in close proximity to Pfs48/45, suggesting that Pfs230 protein trafficking is unaffected in both mutant parasite lines (Figure S6H). Interestingly, both truncated Pfs230 proteins seemed to localize slightly more towards the outside of the cell compared to the PPM-tethered full-length Pfs230, (Figure S6H). When the PVM was naturally disrupted upon activation of macrogametes (Figure 3E, Figure S6I) or chemically disrupted in saponin-treated gametocytes (Figure 3F), Pfs230 remained bound on the PPM surface in wildtype parasites in gamete binding and suspension immunofluorescence microscopy assays. In contrast, both Pfs230^ΔD13/14^ and Pfs230^ΔCterm^ gametocytes and gametes lacked surface-bound Pfs230 in these experiments (Figure 3E-F, Figure S6I). The α-Pfs48/45 signal was found to be slightly higher for Pfs230-truncated parasites in both microscopy and flow cytometry experiments. Together, our structural and *in vivo* experiments demonstrate that the C-terminal region of Pfs230 is essential for the formation of the Pfs230:Pfs48/45 complex.

### Structural characterization of potent Pfs48/45D1-targeting antibody RUPA-71

**Figure 4:**
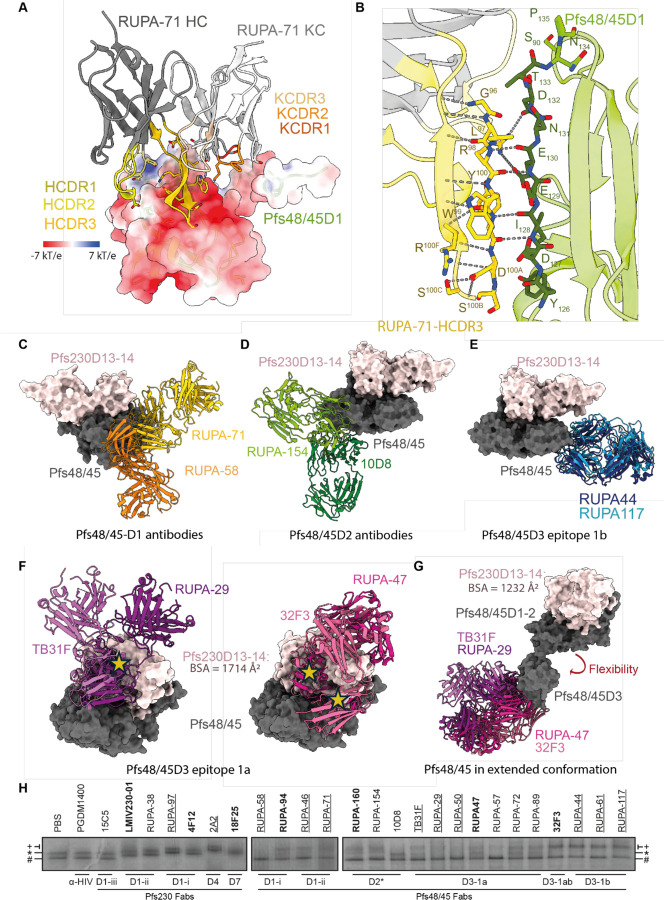
Molecular characterization of anti-Pfs48/45 epitopes in the Pfs230-bound state of Pfs48/45. (A) Structure of RUPA-71 bound to Pfs48/45D1 depicted as surface and coloured by electrostatic potential calculated by Adaptive Poisson-Boltzmann Solver (APBS) and visualized in ChimeraX. (B) RUPA-71 HCDR3 (gold) bound to Pfs48/45D1 (yellow green) with key residues shown as sticks and hydrogen bonds or electrostatic interactions indicated by dashed lines. (C-G) Model of Pfs48/45 (dark gray, depicted in surface) bound to Pfs230D13–14 (misty rose, depicted in surface) in the disc-like (C-F) or extended (G) conformation bound to (C) Pfs48/45D1 binders RUPA-71 (gold), RUPA-58 (orange, PDB ID: 8U1P)); (D) Pfs48/45D2 binders 10D8 (dark green, PDB ID: 7ZXF), RUPA-154 (yellow green, PDB ID: 8U1P)); (E) Pfs48/45D3–1B binders (RUPA-44 (dark blue), RUPA-117 (sky blue, PDB ID: 7UNB)); and (F-G) Pfs48/45D3–1a binders (TB31F (orchid, PDB ID: 6E63), RUPA-29 (dark magenta, PDB ID: 7UXL), RUPA-47 (violet red, PDB ID: 7UNB), and 32F3 (pale violet red, PDB ID: 7ZWI)). Clashes are indicated with yellow stars. (**H**) Blue Native PAGE-based mobility shift assay of FLAG-tag purified Pfs230:Pfs48/45 complex, incubated with a tenfold molar excess of individual anti-Pfs230 or anti-Pfs48/45 Fab fragments. The first lane shows the Pfs230:Pfs48/45 complex with the addition of a mock liquid (PBS) as a reference, and PGDM1400, an anti-HIV envelope protein targeted Fab, was included as negative control. Left panel shows anti-Pfs230 Fabs, right panels show anti-Pfs48/45 Fabs. Fabs are sorted by antibody epitope and potency (underlined: IC_80_ < 10 μg/ml; bold: IC_80_ = 10–100 μg/ml, others: IC_80_ > 100 μg/ml; See also Table S1). Note that due to the nature of Blue Native PAGE, the hydrodynamic radius and therefore the angle of approach can heavily influence the extend of Fab-induced mobility retardation. The symbols #, *, and + denote Pfs230, Pfs230:Pfs48/45, and Pfs230:Pfs48/45:Fab, respectively. See also Figure S7, Table S1 and S3.

The X-ray crystal structure of unliganded RUPA-71 was solved at 2.3 Å resolution (Table S3) to use as a high-resolution model to fit and refine into the 3.4 Å resolution Pfs230D13–14:Pfs48/45:RUPA-44:RUPA-71 cryo-EM map (Figure 4A-B, Table 1). RUPA-71 predominantly interacts with strand 126–135 of Pfs48/45D1, with additional contacts on Pfs48/45D1 loop 88–90 (Figure 4A-B, Table S4). This is consistent with the previous epitope mapping derived from HDX-MS (17). Heavy chain Complementarity-Determining Region 1 (HCDR1; 108 Å^2^), HCDR2 (66 Å^2^), HCDR3 (472 Å^2^), and Kappa chain CDR1 (KCDR1; 52 Å^2^) of RUPA-71 all contact Pfs48/45D1, with the extended HCDR3 (20 aa) contributing most key contacts (Figure 4A, Table S4). The epitope of RUPA-71 is highly charged with several negative and positive patches. RUPA-71 binds to these regions through salt bridges formed between RUPA-71 KCDR1 residue K^32^ and HCDR1–3 residues D^31^ and R^98^ and E_129_, K_89_, K_101_, E_130_, and D_132_ of Pfs48/45D1 (Figure 4A, Table S4). Noticeably, the HCDR3 of RUPA-71 forms a β-hairpin that forms a β-sheet-like structure with two β-strands of Pfs48/45D1 (res. 126–143) (Figure 4B). In addition to intra-chain hydrogen bonds, this β-sheet is held together by five backbone hydrogen bonds between RUPA-71 G^96^, R^98^, and Y^100^ and D_132_, E_130_, and I_128_ of Pfs48/45D1 (Figure 4B, Table S4). Sequence analysis across parasite field isolates revealed only five rare SNPs within the RUPA-71 epitope: K33T (0.015%), K33N (0.007%), S90R (0.007%), K101T (0.029%), and S119T (0.007%), making this epitope highly conserved (Figure S7A) (40). When compared to RUPA-58 which is the only other D1 antibody that has been structurally characterized to date, RUPA-71 binds to a largely separate epitope, although with some overlap in residue contacts (K_88_, K_89_, S_90_, K_101_, D_132_, and R_140_) that would result in steric clashes (Figure S7B) (17). RUPA-58 and RUPA-71, as well as other antibodies that compete with them, require low concentrations to inhibit in the SMFA (IC_80_ < 10 μg/ml), indicating that mAbs that bind to this region of Pfs48/45D1 (res. 86–101 and 126–139) are potently inhibitory (17).

### Pfs48/45 epitope accessibility in the Pfs230:Pfs48/45 complex

Next, we investigated how the Pfs230 interaction with Pfs48/45 might affect the accessibility of Pfs48/45-targeting antibodies. An overlay of structures of previously characterized antibodies with the Pfs230-bound structure revealed that Pfs48/45D1, Pfs48/45D2 and Pfs48/45D3–1b epitope antibodies approach from the side of the Pfs48/45 disc and are accessible when Pfs230 is bound (Figure 4C-E). In contrast, many antibodies that target the Pfs48/45D3–1a epitope, including highly potent antibodies TB31F and RUPA-29, would have considerable clashes with both Pfs230D13 and Pfs230D14 in this conformation (Figure 4F-G) (17).

To determine whether these antibodies can bind to Pfs48/45 in the context of the Pfs230:Pfs48/45 complex, we used the BN-PAGE-based mobility shift assay to study the changes in migratory pattern of Fab-bound endogenous Pfs230:Pfs48/45 complex. To this end, the purified complex was incubated with tenfold molar excess Fab derived from a range of both potent and non-potent TRA antibodies, spanning almost all currently known epitopes on the complex (Figure 1A). As expected, α-Pfs230 Fabs shifted both bands upwards, with the exception of 15C5, which targets epitope III on Pfs230D1 that is shielded by Pfs230D2 and thus incompatible with binding full-length Pfs230 (Figure 4H) (19, 22). None of the other tested α-Pfs230 Fabs, targeting epitopes on Pfs230D1, Pfs230D4 and Pfs230D7, disrupted the Pfs230:Pfs48/45 complex, in agreement with the relative orientation of Pfs230 on Pfs48/45 in our structure. The α-Pfs48/45 Fabs did not shift the lower free Pfs230 band, but did affect migration of the upper band. While some Fabs i) bound to the intact complex (*e.g.* RUPA-44, RUPA-117), others ii) disrupted the Pfs230:Pfs48/45 complex (*e.g.* RUPA-58 and RUPA-50), or iii) showed a heterogeneous effect resulting in Fab-bound and unbound Pfs230:Pfs48/45, as well as disrupted protein complex (*e.g.* TB31F, RUPA-47) (Figure 4H). The non-potent 10D8, targeting Pfs48/45-D2, could not bind to the Pfs230:Pfs48/45 complex. To confirm the observation that some but not all Pfs48/45-targeted antibodies could disrupt the Pfs230:Pfs48/45 complex, we tested whether a subset of these Fabs could displace Pfs230 on the female gamete surface in a competition assay. In agreement with the BN-PAGE results, RUPA-50, RUPA-57, and RUPA-58 significantly decreased Pfs230 on the gamete surface in a dose-dependent manner, while *e.g.* TB31F, RUPA-46, and RUPA-89 Fabs showed no displacement of Pfs230 (Figure S7C).

Intriguingly, TB31F could still partially bind to the Pfs230:Pfs48/45 complex in BN-PAGE (Figure 4H) and did not displace Pfs230 on the gamete surface (Figure S7C), despite showing significant steric clashes in our Pfs230-bound Pfs48/45 structure. In this context, it is interesting to note again that it has previously been described how Pfs48/45 can adopt a disc-like conformation (similar to the structure reported here in complex with Pfs230), but also more extended conformations where Pfs48/45D3 can reorient from the rest of the protein (17). Given that Pfs48/45D1–2 contributes 1169 Å^2^ of a total 1647 Å^2^ buried surface area within the Pfs48/45-Pfs230 interface, it is conceivable that Pfs48/45D1–2 is sufficient for Pfs230 binding, allowing for Pfs48/45D3 to retain a range of motion in the complex. A superimposition of Pfs48/45D3 antibodies onto the previously solved extended conformation of Pfs48/45 with Pfs230D13–14 aligned to its Pfs48/45D1–2 binding site demonstrates that Pfs48/45D3–1a epitope would be accessible in this arrangement (Figure 4G). This elongated conformation can also accommodate binding for most anti-Pfs48/45 antibodies that target epitope 1b or Pfs48/45D1–2 (Figure S7D). Together, this could indicate that Pfs230-bound Pfs48/45 retains flexibility, and that this mobility may allow for the binding of antibodies that would otherwise be blocked in the Pfs230-bound disc-like conformation of Pfs48/45.

Interestingly, the ability of Fab fragments to induce partial disruption of the Pfs230:Pfs48/45 complex does not seem to be associated with TRA of the corresponding antibody in SMFAs (Figure 4H, Figure S7C, Table S1). This is illustrated by Pfs48/45D3–1a targeting antibodies RUPA-57 (IC_80_ > 50 μg/ml) and RUPA-50 (IC_80_ < 2 μg/ml) that are both able to fully dissociate the complex *in vitro* and partially *in vivo*, but have very different potencies. Also, the very potent TB31F (IC_80_ < 2 μg/ml) and the non-potent RUPA-89 (IC_80_ >100 μg/ml) show only partial dissociation *in vitro* and no displacement of Pfs230 *in vivo*, but again have very different potencies. Together, these data strongly suggest that Pfs230:Pfs48/45 complex disruption is not a generalizable mode-of-action for functional α-Pfs48/45 and α-Pfs230 antibodies.

### Structural delineation of potent Pfs230D4 and D7 epitopes

**Figure 5:**
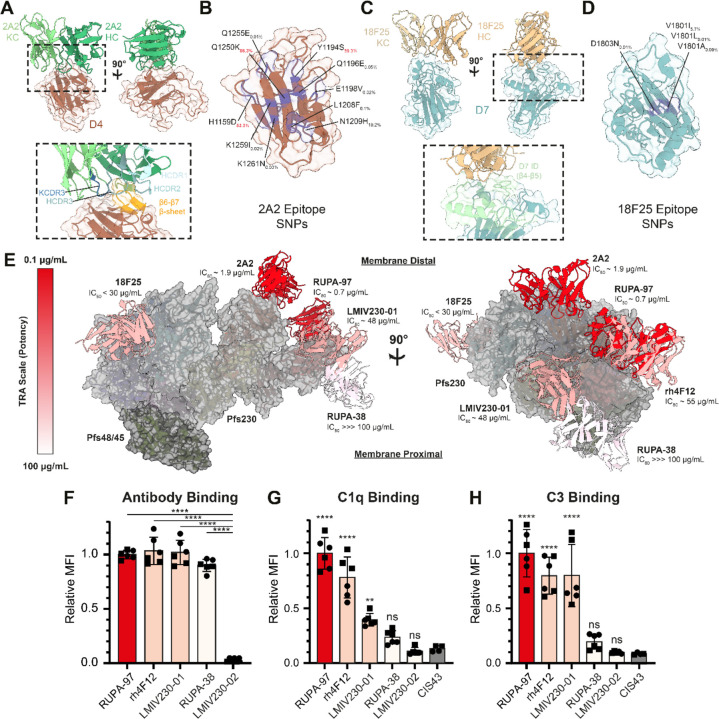
Molecular basis of Pfs230-directed mAb potency. (A) The variable region of 2A2 (green) and Pfs230D4 (orange) are shown with key contacts (inset) made by the HCDR3, KCDR1, KCDR2, and KCDR3 (shades of blue) of mAb 2A2 with the β6-β7 perpendicular β-hairpin (yellow) of Pfs230D4 highlighted. (B) SNPs of Pfs230D4 identified in the MalariaGEN Pf7 database (54) are indicated in purple and labeled. The frequency of the SNP is shown as a subscript with values exceeding 1% coloured in red. (C) The variable region of 18F25 (tan) and Pfs230D7 (cyan) are shown. Key contacts made by 18F25 with Pfs230D7 (inset) include the ID of Pfs230D7 (light green). (D) SNPs of Pfs230D7 identified in the MalariaGEN Pf7 database (54) are indicated in purple and labeled. The frequency of the SNP is shown as a subscript. (E) The binding sites of 18F25, LMIV230–01, 2A2, RUPA-97, RUPA-38, and rh4F12 shown in the context of Pfs230 (surface: gray, ribbons: domains coloured as done in Figure 1 and 2) and Pfs48/45 (surface: black, ribbons: domains coloured as done in Figure 1). The antibodies are coloured in accordance with their IC_80_ values or TRA via heat map with darker colours indicating higher TRA or potency (left scale). The membrane distal side corresponds to the top of the panel and the membrane proximal side at the bottom of the panel. (F-H) Anti-Pfs230D1 mAb binding (F), or antibody-mediated human C1q recruitment (G) and human C3 deposition (H) at 1 μg/ml on the surface of wildtype NF54 macrogametes as determined by flow cytometry. CIS43, an anti-PfCSP antibody, was included as a negative control for the C1q and C3 deposition assays. Mean fluorescence intensity (MFI) values were normalized against the mean of RUPA-97 replicates, to allow averaging across two independent experiments (depicted as different symbols) with two to three technical replicates each. Mean ± standard deviation is shown. Statistical analysis was done using an ordinary one-way ANOVA with either a Tukey’s multiple comparisons test with a single pooled variance comparing all groups to each other (F, only showing significant comparisons) or Dunnett’s multiple comparisons test with a single pooled variance comparing all groups to CIS43 (G-H). ns = not significant; **=p<0.01, ****=p<0.0001. See also Figure S8 and Table S1.

Our cryo-EM studies reveal potent epitopes outside of Pfs230D1 that had not been previously structurally elucidated. Fab 2A2 could be resolved in the Pfs230D1–6 map, while Fab 18F25 could be resolved in the Pfs230D7–8 map (Figure 2A, Table 1). X-ray crystal structures of unliganded 2A2 and 18F25 Fabs were solved to 1.2 Å and 1.6 Å resolution (Table S3), respectively and docked and refined into the maps. Our cryo-EM structure reveals that 2A2 targets Pfs230D4 (Figure 5A), confirming the epitope location previously proposed based on the differential binding and TRA of mAb 2A2 against *P. falciparum* strains that contain SNPs in Pfs230D4 (40). 2A2 buries 824 Å^2^ on Pfs230D4, primarily making contacts through the HCDR1/3 and KCDR2/3 loops as well as the N-terminus of the heavy chain (Table S4). Most residues contacted by the antibody lie in the canonical perpendicular β-hairpin of Pfs230D4, and a moderate number in the loop region between β9 and β10 (Figure 5A, Table S4). A subset (H1159D, Y1194S, Q1196E, N1209Y, Q1250K) of the SNPs found in the strains of *P. falciparum* against which 2A2 had diminished TRA localize to the epitope (Figure 5B) (45). In addition, analysis of the MalariaGEN Pf7 database reveals that more SNPs differing from the 3D7 strain are found in the epitope (Figure 5B) (54). Some of the SNPs are quite rare with frequencies less than 1% (Q1255E, K1259I, K1261N, Q1196E, E1198V, L1208F, and N1209Y), while others are seemingly more common (Q1250K – 86.3%, Y1194S – 59.3%, and H1159D – 53.3%).

The epitope of mAb 18F25 localizes to Pfs230D7 (Figure 5C) (38). 18F25 buries 515 Å^2^ on Pfs230D7 through all CDRs of the antibody (Table S4). Our structure reveals that 18F25 primarily engages with the Pfs230D7-ID (Figure 2H and 2K) found in between β4 and β5, with a small amount of interactions localized to the β8-β9 loop (Figure 5C, Table S4). Unlike the 2A2 epitope and Pfs230D4 overall, both the 18F25 epitope and Pfs230D7 are more conserved (Figure 5D). Only two SNPs in Pfs230D7 identified from the MalariaGEN Pf7 database have frequencies higher than 10% (Y1829N – 69.4% and I1870V – 13.5%) and these are not contained within the 18F25 epitope (54). Only two residues within the epitope are reported with mutations in the database. V1801I, V1801L and V1801A, are conservative mutations that have a combined frequency of 5.8% (5.7%, 0.01%, and 0.09%, respectively). The relatively conservative D1803N mutation has only been observed in 0.01% of strains. Together, elucidation of the 18F25 and 2A2 epitopes on Pfs230D4 and D7 provide much-needed structural insights into Pfs230-directed antibody response outside of Pfs230D1.

### Potent anti-Pfs230 antibodies target membrane distal epitopes

mAb 2A2, while showing strain-dependent TRA, was highly potent in SMFAs utilizing the NF54 strain (IC_80_ = 1.9 μg/ml (40)). The potency was on par with the highly potent RUPA-97 that targets Pfs230D1 (IC_80_ = 0.7 μg/mL (22)), suggesting that a common feature of these mAbs might be responsible for the high potency. These two mAbs, despite binding different domains, bind to the same side of Pfs230 with a similar angle of approach (Figure 5E). mAb 18F25 (IC_80_ < 30 μg/ml (38)) also binds to the same face of Pfs230, while differing slightly in its angle of approach (Figure 5E). Interestingly, Pfs230D1-directed mAbs of lower potency, such as RUPA-38 (IC_80_ > 100 μg/mL) (22), bind to the opposite face of where RUPA-97, 2A2, and 18F25 bind to Pfs230 (Figure 5E). This trend is generally applicable to α-Pfs230D1 mAbs published to date (Figure S8) (19).

These Pfs230-targeting mAbs have been demonstrated to be complement-dependent for their TRA (22, 38–40, 42). Previously, it has been shown that for efficient C1q deposition on an antigenic surface, the first step in the classical complement activation pathway, IgGs need to form into ordered hexameric antibody structures (55, 56). Intriguingly, the epitopes of potent mAbs RUPA-97, 2A2, and 18F25 are localized to the opposite face of where Pfs48/45 and presumably the PPM would be (Figure 5E). In contrast, the epitope of non-potent RUPA-38 is localized near the presumed location of the PPM (Figure 5E). This correlation led us to hypothesize that the angle of approach of the antibody is a determinant of Pfs230-directed antibody potency, as antibodies that are near the PPM might be prevented from efficient recruitment of complement components and thus complement activation (Figure 5E). To test this hypothesis, we selected five different mAbs targeting Pfs230D1 with different potencies (RUPA-97 (IC_80_ = 0.7 μg/mL (22)), rh4F12 (IC_80_ = 55 μg/mL (22)), LMIV230–01 (IC_80_ = 48 μg/mL (22)), RUPA-38 (IC_80_ > 100 μg/mL) (22)), and LMIV230–02 (IC_80_ > 1,000 μg/mL (21)), expressed these as IgGs of the same complement-fixing subclass, and tested antibody binding, C1q recruitment and C3 deposition on live female NF54 gametes. All antibodies except LMIV230–02 bound gametes with similar intensity (Figure 5F), which is in agreement with previously determined *in vitro* affinities (22). In contrast, the highly potent antibody RUPA-97 and moderately potent antibodies rh4F12 and LMIV230–01 were able to recruit C1q and deposit C3 on the surface, while the non-potent RUPA-38 was not able to do so efficiently (Figure 5G-H). These findings, as implied by the Pfs230:Pfs48/45 complex structure, suggest that the angle of approach relative to the PPM might be a critical determinant for potency in complement-dependent Pfs230-directed antibodies.

## Discussion

Targeting human-to-mosquito transmission, a developmental bottleneck in the *Plasmodium* life cycle, is a promising intervention strategy for malaria control and elimination. Here, we reveal the structural disposition of the heterodimer complex Pfs230:Pfs48/45, which is essential for *P. falciparum* transmission and a major target for malaria transmission-blocking vaccine development. Using advances in molecular parasitology and cryo-EM, we were able to circumvent recombinant expression by purifying the approximately 410 kDa large, membrane-bound Pfs230:Pfs48/45 complex from its native source, late-stage *P. falciparum* gametocytes. Previously, similar methods have been used to elucidate the structure of the *Plasmodium* Translocon of Exported Proteins (PTEX) complex (57) and the soluble RhopH complex (58, 59), both crucial protein complexes within the *Plasmodium* life cycle non-amenable to standard recombinant protein production. Using genome engineering to enable the purification of endogenous proteins for cryo-EM studies has also found traction in research on other organisms, including human cells (60), and bacterial pathogens like *Pseudomonas spp.* (61) and *Mycobacteria spp.* (62). This highlights the opportunities that this approach, combined with recent advances in the field of structural biology including bottom-up structural proteomic platforms such as cryoID (63) and protein folding prediction software such as AlphaFold 3 (64), may offer to the many other *Plasmodium* proteins refractory to recombinant expression.

**Figure 6.**
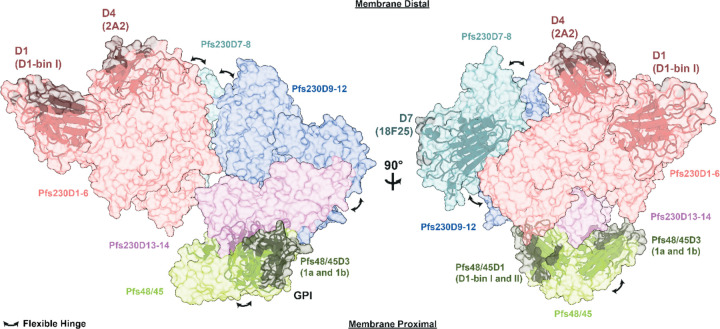
Structural model of the Pfs230:Pfs48/45 complex and potent epitopes. Domain clusters of Pfs230 and Pfs48/45 are indicated (Pfs230D1–6: red, Pfs230D7–8: teal, Pfs230D9–12: blue, Pfs230D13–14: pink, and Pfs48/45: green). Flexible hinges within the complex are indicated as a double-sided arrow. Domains targeted by potent mAbs (IC_80_ < 30 μg/mL) are shown as ribbons with the surfaces of these epitopes targeted by these antibodies coloured in a darker shade. Surfaces of epitopes are based off RUPA-97 (Pfs230D1-bin I), 2A2, 18F25, RUPA-58 (Pfs48/45D1-bin I), RUPA-71 (Pfs48/45D1-bin II), TB31F (Pfs48/45D3–1a), and RUPA-44 (Pfs48/45D3–1b).

Our structure reveals how insertion domains present within Pfs230 6-Cys domains mediate inter-domain interactions. Regions without ID-mediated interdomain contacts show increased flexibility, which results in the Pfs230 structure being segmented into four rigid domain clusters of relative flexibility in relation to one another (Pfs230D1–6, Pfs230D7–8, Pfs230D9–12 and Pfs230D13–14; Figure 6, Figure S3). ID-mediated inter-domain contacts have also been observed in the Pf12-Pf41 heterodimer complex, which are two other malarial proteins that are composed of 6-Cys domains (52, 53). Additionally, inter-protein contacts between Pfs230 and Pfs48/45 appear to be mediated by regions outside of the canonical 6-Cys fold; we have shown how the C-terminal extension of Pfs230 is critical for binding, and observed that the N-terminal extension of Pfs48/45 also localized to this interface. This suggests that regions outside of the canonical 6-Cys fold may have been introduced to provide functional roles by mediating interactions, which may be a generalizable property of 6-Cys domain family of proteins.

Although it is currently unknown what exact cellular process the Pfs230:Pfs48/45 complex is involved in, knockout studies in *P. falciparum* and the rodent malaria parasite *Plasmodium berghei* suggest the Pfs230:Pfs48/45 complex is involved in microgamete-to-macrogamete adhesion and microgamete-to-RBC adhesion (8–10). It is tempting to speculate that flexibility between Pfs230 domain clusters might play a role in its cellular function. The presented structure will enable the design of domain clusters with more precision than previously possible for the generation of genetically modified parasite lines that can interrogate Pfs230 function. While the C-terminal Pfs230 domains are involved in the Pfs48/45-based membrane anchoring onto the PPM, the N-terminal domains are free to interact with other elements. The previous descriptions of some functional complement-independent α-Pfs230D1 antibodies suggest that Pfs230D1 might be involved in some protein interactions that are disrupted upon binding of these antibodies (18, 36). Interestingly, the addition of recombinant Pfs230Pro-D1–2 was previously shown to inhibit the formation of exflagellation centers (18), further suggesting that the N-terminus of Pfs230 might be involved in cell-to-cell adhesion. On the Pfs48/45 side, we showed that functional activity of complement-independent Pfs48/45 antibodies does not directly correlate with their ability to disrupt the Pfs230:Pfs48/45 complex. This implies that some Pfs48/45 targeting antibodies may prevent interactions of Pfs48/45 with other proteins and that Pfs48/45 does not merely serve as Pfs230 membrane retention. Future studies will continue to provide insights into interaction partners of the Pfs230:Pfs48/45 complex and uncover the roles of Pfs230 and Pfs48/45 in parasite transmission.

Here, we found that Pfs230 binds to the GPI-anchored Pfs48/45 through its C-terminal peptide. It has been reported that Pfs230 can be membrane-bound in the absence of Pfs48/45 in a small subpopulation of female macrogametes via a currently unknown mechanism (20). We did not observe any Pfs230^ΔCterm^ macrogamete or gametocyte still harboring membrane-bound Pfs230, suggesting that the presumed Pfs48/45-independent membrane-binding of Pfs230 is mediated through the same Pfs230 C-terminal peptide. Previously, co-immunoprecipitation experiments have suggested the existence of additional interacting partners in the Pfs230:Pfs48/45 complex, including a large complex consisting of six adhesion-linked *Pf*CCp proteins, the PPM-bound and macrogamete-specific protein Pfs25, and the WD40-repeat protein PfWLP1 (65–67). Interestingly, we could not detect enrichment for any of these proteins after FLAG-tag based co-immunoprecipitation performed on lysates from late-stage iGP2^230-tag^ gametocytes (Figure 1D), although we cannot exclude that differences between the protocols used might have disrupted more labile interactions. Future studies using the iGP2^230-tag^ line might reveal additional targets for TBVs by fully characterizing the protein-interaction network of Pfs230:Pfs48/45, throughout all sexual parasite stages.

Next to characterizing the full-length structure of the Pfs230:Pfs48/45 heterodimer, we also molecularly characterized the epitope of multiple transmission-blocking antibodies. The potent Pfs48/45D1 antibody, RUPA-71, binds an adjacent epitope to the previously characterized mAb, RUPA-58 (17). All highly potent Pfs48/45D1 antibodies compete with either RUPA-58 or RUPA-71, indicating that binding to this conserved portion of Pfs48/45D1 is associated with transmission-reducing activity (17). Additionally, we structurally characterized potent epitopes outside of Pfs230D1 found on Pfs230D4 and Pfs230D7 targeted by murine mAbs, 2A2 and 18F25, respectively. While mAb 2A2 is notably potent when used against the NF54 strain (40, 51), the epitope is highly polymorphic with many Pfs230D4 SNPs observed in field isolates (40) and described in the MalariaGEN Pf7 database (54). In contrast, both Pfs230D7 and the 18F25 epitope are highly conserved and may thus be an ideal domain for subunit vaccine development. However, in comparison to 2A2 and highly potent Pfs230D1-bin I binders, 18F25 exhibits approximately 10-fold lower potency. The ability to design Pfs230 probes based on the full-length structure will now enable discovery efforts seeking more potent antibodies. In addition, the structure of the full Pfs230:Pfs48/45 heterodimer complex reported here with structure-function relationships of antibody inhibition, now enables a more fulsome opportunity for structure-guided immunogen design, which has been transformative in vaccinology during the development of *e.g.* SARS-CoV-2, and respiratory syncytial virus vaccines (68, 69).

Immunogens based on Pfs230 and Pfs48/45 have already shown promising results in clinical trials (46–49), though the induced inhibitory activity was incomplete and the durability of the response remains uncertain. These findings suggest that current TBV candidates can be further optimized, for example by including Pfs230 domains that were previously thought to be unable to induce functional transmission-blocking antibodies (38–40), or by rational structure-based immunogen design (70, 71). Currently, in the case of Pfs230 domains, Pfs230D1, D4, D7, and D12 have been demonstrated to elicit potent antibodies (22, 38–40). Interestingly, these Pfs230 domains share a common feature in that they are membrane distal, suggesting that domains present on this side should be prioritized for subunit vaccine development. By this logic, Pfs230D8 and D9 may also be relevant targets (Figure 6). To date, most Pfs48/45-based interventions focus on Pfs48/45D3, but Pfs48/45D1 epitopes may be beneficial inclusions into future Pfs48/45-based immunogens. Future immunogen design efforts should integrate inter-domain structural elements to present well-folded epitopes. Approaching immunogen design through stabilizing structural clusters instead of individual domains will help eliminate the presentation of non-neutralizing epitopes that are absent endogenously, as previously evidenced by the elicitation of Pfs230D1-binIII mAbs (15C5, LMIV230–02, and 230AS-26) and 230AL-20 by a Pfs230D1-only antigen (Figure S8). The presented Pfs230:Pfs48/45 structure might also allow for future protein-engineering campaigns that graft multiple potent epitopes onto a single chimeric immunogen (72), or use stabilizing mutations in conjunction with display on nanocages (73). Additionally, advances in mRNA vaccination approaches combined with precise immunogen design might allow for the *in situ* expression of stable, native Pfs230:Pfs48/45 co-assemblies. Ultimately, the aim will be to create the most potent next-generation TBVs possible to aid in meeting the ambitious goals of malaria elimination and eradication.

### Limitations of the study

One limitation of our structure is the low resolution obtained for the Pfs230D9–12 domains (global resolution of 4.7 Å). This prevented us from building an atomic model for these domains, limiting the information we could confidently extract from this part of the map. Instead, we combined this low-resolution map with an AF-based modeling approach to obtain a backbone model with reasonable confidence regarding the domain orientations in this region of Pfs230. The flexibility of the different structural units in Pfs230, in conjunction with the flexibility within membrane-anchored Pfs48/45, suggests Pfs230 likely has a considerable range of motion on the parasite surface (Figure 6 and S3). Future studies should look to elucidate whether this flexibility plays an important role in the function of Pfs230:Pfs48/45. Finally, we purified the Pfs230:Pfs48/45 from late-stage gametocytes instead of gametes, due to practical reasons. After gametogenesis, however, the Pro-domain of Pfs230 is proteolytically cleaved (74), and therefore our structure does not fully recapitulate the mature protein species. How these additional Pro-domain residues add onto the structure and if these influence the overall Pfs230:Pfs48/45 conformation, remains currently unknown.

## Resource availability

### Lead contact

Further information and requests for resources and reagents should be directed to and will be fulfilled by the lead contact, Jean-Philippe Julien (jean-philippe.julien@sickkids.ca) upon reasonable request.

### Materials availability

All unique reagents generated in this study are available from the lead contact with a completed Materials Transfer Agreement.

### Data and code availability

- Previously unpublished antibody sequences are available in Table S6. Crystal and cryo-EM structures have been deposited in the Protein Data Bank and are publicly available as the date of publication. EMDB and PDB IDs are listed in Table 1 and Table S3. The proteomics dataset has been deposited in the ProteomeXchange Consortium via the PRIDE partner repository (75) with the dataset identifier PXD060716.
- This paper does not report original code.
- Any additional information required to reanalyze the data reported in this paper is available from the lead contact upon reasonable request.

## Methods

### *Plasmodium falciparum* genome modification plasmids

To create the Cas9 guide plasmids, a pair of complementary guide-encoding oligonucleotides 5’-TTAATGATGGCTCTTGATTG-3’ (targeting Pfs230 C-terminus to create iGP2^230-tag^ / 230^ΔD13/D14^ / 230^ΔCterm^) and 5’-ATTCTATTACATTATCAAGA-3’ (targeting Pfs230D13 to create 230^ΔD13/14^) were treated with T4 polynucleotide kinase, annealed, and ligated into BbsI-digested pMLB626 (a kind gift from Marcus Lee) (77).

To generate the iGP2^230-tag^ homology-directed repair (HDR) template, partial 5’ and full 3’ homology regions (HR) were PCR amplified from NF54 genomic DNA (Table S5). Synonymous mutations to shield the Cas9 guide region in the full 5’HR were inserted by overlap PCR using the partial 5’HR and two annealed custom-made oligonucleotides (Sigma-Aldrich, Table S5). A selection cassette containing the i) PfH2B promoter, ii) the *mScarlet* gene, and iii) a bidirectional 3’UTR (PBANKA_142660) was PCR amplified from an in-house plasmid (78) as two separate amplicons to remove internal restriction sites. These two amplicons were cloned into pGGASelect using a BsaI-HFv2 Golden Gate reaction, and further sub-cloned using AatII and BamHI-HF into a pUC19-based plasmid that had all internal type IIS restriction enzymes removed via site-directed mutagenesis, yielding pRF0508. The final HDR template was created using the NEBridge Golden Gate Assembly Kit (BsmBI-v2), combining i) pGGAselect, ii) the 5’ HR amplicon, iii) two annealed oligonucleotides coding for a tandem affinity purification tag (GTSG-(3xFLAG)-GSG-EPEA-stop) (Table S5), iv) pRF0508, and v) the 3’ HR amplicon.

To generate HDR templates to make Pfs230^ΔD13/14^ and Pfs230^ΔCterm^, the respective 5’HRs (Pfs230^ΔD13/D14^ spanning Pfs230D11–12, Pfs230^ΔCterm^ spanning Pfs230D13–14) and 3’HR were PCR amplified from NF54 genomic DNA (Table S5). The selection cassette was modified by swapping the PfH2B promoter in pRF0508 for the PfGAPDH promoter (Pf3D7_1462800). The HDR templates were created in a golden gate reaction combining i) pGGAselect, ii) respective 5’HR amplicon, iii) PCR amplicon coding for (GGSG-3xFLAG-stop), iv) GAPDH selection marker, and v) the 3’ HR amplicon.

All PCR reactions were performed using Primestar^®^ GXL DNA polymerase (Takara Bio), all other cloning enzymes were obtained via New England Biolabs. Sequences were confirmed by Sanger Sequencing (Baseclear).

### *Plasmodium falciparum* culturing, transfections, and gametocyte production

*P. falciparum* cultures were maintained at 37 °C with 3% O_2_ and 4% CO_2_, in complete medium (RPMI1640 medium with 25 mM HEPES, 25 mM NaHCO_3_, 10% human type A serum) with 5% O^+^ human red blood cells (Sanquin, The Netherlands) (79). Asexual NF54/iGP2 and NF54/iGP2^230-tag^ parasites were cultured in the presence of 2.5 mM D-(+)-glucosamine hydrochloride (Sigma #1514). Parasites were synchronized using sorbitol treatment before transfection or gametocyte induction (80).

For transfection, 80 μg of HDR plasmid was linearized overnight, ethanol precipitated and co-transfected with 80 μg of Cas9 guide plasmid by ring-stage transfection as described previously (81, 82). Briefly, plasmids were resuspended in cytomix (10 mM K_2_HPO_4_/KH_2_PO_4_ pH 7.6, 120 mM KCl, 0.15 mM CaCl_2_, 5 mM MgCl_2_, 25 mM HEPES, 2 mM EDTA) and added to a 3% ring-stage NF54/iGP2 (to generate iGP2^230-tag^) or NF54 (to generate 230^ΔD13/D14^ / 230^ΔCterm^) culture. Parasites were electroporated (310 V, 950 μF), allowed to recover for 4 h, after which transfected parasites were selected using 2.5 nM WR99210 (Jacobus Pharmaceuticals) for five days. To obtain single-cell clones for iGP2^230-tag^, late-stage parasites were stained using 1 μg/ml Rhodamine123 solution, and single-cell sorted using a FACSAria III Cell Sorter (BD Biosciences) in a round-bottom 96 well plate containing 100 μl complete medium with 3% haematocrit. To obtain an isogenic population for 230^ΔD13/D14^ and 230^ΔCterm^, parasites were sorted based on mScarlet expression using a Cytoflex SRT Benctop Cell Sorter (Beckman Coulter). Successful integration was confirmed by diagnostic PCR (Table S5).

To induce gametocytogenesis, a 1% early trophozoite culture (±24 hours post invasion) was induced with AlbuMAX medium (RPMI1640 supplemented with 25 mM HEPES, 25 mM NaHCO_3_, and 0.5% AlbuMAX-II (Gibco, #11021–045)), in a previously described automatic shaker setup with media changes every 12 h (83, 84). Additionally, glucosamine was removed from iGP2-based cultures to further increase gametocyte induction. 36 h post induction until completion, cultures were maintained in complete medium. From day 4 – 8 post induction, gametocytes were cultured in the presence of 20 units/ml heparin (Sigma, #H3393) to eliminate asexual parasite growth. To obtain late-stage gametocyte saponin pellets, gametocytes were harvested on day 13 post induction by incubating the cells for 10 min in a 10x pellet volume ice-cold 0.06% (w/v) saponin in PBS supplemented with 1x Complete^™^ Protease Inhibitor (PI) Cocktail (Roche), and subsequent centrifugation at 3,000xg for 5 min at 4 °C. Pellets were washed three times in ice-cold PBS with 1x PI, flash frozen and stored at −70 °C.

### Western blot

Saponin pellets from late-stage gametocyte cultures were resuspended in lysis buffer (10 mM Tris-HCl (pH 8.0), 150 mM NaCl, 1% (w/v) sodium deoxycholate, and 1x PI) for 15 min at RT. Lysate was cleared by centrifugation, and supernatant was supplemented with 1x NuPAGE LDS buffer, and heated to 56 °C for 15 min. Samples were loaded on 4–12% Bis-Tris SDS-PAGE gels (SurePAGE), using Precision Plus Dual Color (BioRad) as a reference. After electrophoresis, proteins were transferred to 0.45 μm Immun-Blot PVDF membrane (Bio-Rad) using the Trans-Blot Turbo transfer system (Bio-Rad). Membranes were blocked in PBS with 3% BSA, before incubation with primary antibody diluted in PBS-T with 1% BSA. The following primary antibodies were used: 18F25 (mouse anti-Pfs230 (38), 5 μg/ml), 32F3 (mouse anti-Pfs48/45 (27), 5 μg/ml), M2 (mouse anti-FLAG, F1804 Sigma, 1:2000), CaptureSelect^™^ (biotinylated single domain antibody fragment anti-C-tag, Thermofisher, 1:1000). After washing, blots were incubated with one of the following secondary antibodies: HRP-conjugated rabbit anti-mouse (P0260, Dako, 1:2000), HRP-conjugated streptavidin (890803, R&D Systems, 1:500). Blots were developed with Clarity Western ECL substrate (BioRad) and imaged on an ImageQuant^™^ LAS 4000 (GE Healthcare).

### Immuno-fluorescence microscopy

Heparin-treated stage V gametocytes (day 13) were fixed (4% EM-grade paraformaldehyde, 0.0075% glutaraldehyde in PBS) for 20 min on pre-warmed poly-L-lysine coated coverslips at 37 °C. Cells were permeabilized with 0.1% Triton X-100, washed in PBS and blocked using 3% BSA in PBS for 1 h. Primary and secondary antibodies were incubated for 1 h at RT, with PBS washes in between. Primary antibodies were diluted in 1% BSA in PBS, and included M2 (mouse anti-FLAG, F1804 (Sigma), 1:500), RUPA-55 mAb (human anti-Pfs230 (22), 5 μg/ml), and 45.1 mAb (rat anti-Pfs48/45 (27), 5 μg/ml). Secondary antibodies (goat anti-mouse 594 (A11031), goat anti-human 488 (A11013), and chicken anti-rat 674 (A21472) (ThermoFisher)) were all diluted 1:500 in PBS with 1 μM DAPI. Coverslips were mounted using Vectashield^®^ Antifade Mounting Medium (Vector Laboratories). Images were taken on a Zeiss LSM900 Airyscan confocal microscope using a 63x oil objective with 405, 488, 561, and 633 nm laser excitation and an Electronically Switchable Illumination and Detection Model (ESID) for transmitted light. Images were processed for airyscan, using the Zeiss Zen Blue software and analysed using FIJI software (85).

### Suspension Immunofluorescence Microscopy (SIFA)

For gamete SIFAs, heparin-treated stage V gametocytes (day 13 post-induction) were collected, spun down to remove the culture medium, and resuspended in half the culture volume of FCS. After incubation with gentle agitation at RT for 1 h, parasites were spun down and washed in ice-cold PBS. For saponin-treated gametocytes, heparin-treated stage V gametocytes (day 13 post-induction) were collected, spun down, and resuspended in half the volume of ice-cold PBS supplemented with 0.06% Saponin and 1x protease inhibitor. After 10 min incubation on ice, saponin-treated gametocytes were harvested by centrifugation and washed three times in ice-cold SIFA buffer (PBS supplemented with 0.5% FCS). All subsequent steps were performed at 4 °C.

Both gametes and gametocytes were incubated for 1 h in SIFA buffer with 15 μg/ml mAb 18F25 or 45.1, labeled with LYNX Rapid Plus DyLight488 Antibody Conjugation Kit (Bio-Rad). After washing in ice-cold SIFA buffer, parasites were resuspended in ice-cold PBS and deposited in pre-cooled poly-L-lysin-coated μ-slide 8 well Ibidi chambers. Parasites were allowed to settle at 4 °C, after which the Ibidi chamber was transferred to a room-temperature Axio Observer 7 Inverted LED microscope equipped with a Colibri 7 LED source and Axiocam 705 mono (Zeiss). Images were taken with a 63x oil objective using the 475 nm LED module and a Differential Interference Contrast enhanced brightfield channel. Images for different parasite lines were taken on the same day with the same microscope settings, and were analyzed with the same settings in FIJI software (85).

### Exflagellation assay and standard membrane feeding assay

Day 15 cultures of non-heparin treated gametocytes were used to test exflagellation. Cultures were mixed 1:1 with 50 μM xanthurenic acid in complete media, and incubated at room temperature in a dark, humid chamber for 15 min. The number of exflagellation centres per ml was determined by bright-field microscopy using a Neubauer chamber. To assess mosquito infectivity, day 15 non-heparin treated gametocytes were added to the bloodmeal of *Anopheles stephensi* mosquitoes (colony maintained at Radboudumc (Nijmegen, The Netherlands)), as described previously (86). Mosquito midguts from 10 – 20 mosquitoes were dissected 6–8 days after the blood meal, stained with mercurochrome, and oocysts were counted by microscopy.

### Affinity purification of Pfs230:Pfs48/45 complex from stage V gametocytes

Frozen stage V gametocyte pellets were resuspended in solubilization buffer (25 mM HEPES pH 7.4, 150 mM KCl, 10% glycerol, 1x PI, and 0.25% (v/v) DDM), and rotated at 4 °C for 1 h. The cell suspension was centrifuged at 18,000xg at 4 °C for 30 min, after which the pellet was solubilized a second time following the previous steps. The combined supernatants were applied to anti-FLAG^®^ M2 affinity resin (Merck), and incubated overnight at 4 °C. The resin was extensively washed in Wash buffer (25 mM HEPES pH 7.4, 150 mM KCl, 10% glycerol, 1x PI, and 0.02% DDM), after which proteins were eluted using 5x resin volume of elution buffer (25 mM HEPES pH 7.4, 150 mM KCl) supplemented with 0.02% DDM, and 150 μg/ml 3xFLAG peptide (Merck)). For cryo-EM studies, the eluted proteins were mixed with a fivefold molar excess of RUPA-97, LMIV230–01, 2A2, 18F25, RUPA-71, and RUPA-44 Fabs and incubated on ice for 30 min. After concentrating the protein fraction, the Fab-bound complex was separated from unbound Fabs by high-pressure size exclusion chromatography using a Bio SEC-3 300 Å column (Agilent, 5190–5213), pre-equilibrated with elution buffer.

### Expression and purification of Fabs, mAbs, and recombinant proteins

Expression and purification of individual Fabs was performed as described previously (22, 34). Variable light and heavy chains were gene synthesized (GeneArt), and cloned into a custom pcDNA3.4 expression vector directly upstream of the constant chains. The variable chain sequences for 32F3, TB31F, LMIV-230s, and all RUPA mAbs were described previously (22, 34, 43). Variable sequences for 18F25 (Table S6) were determined by a previously published workflow for hybridoma sequencing (76). In short, RNA was obtained from 18F25.1 hybridoma cells by Trizol purification, which was used as template for RT-PCR with heavy/kappa constant chain specific reverse primer and a universal template-switching oligonucleotide (Table S5). The obtained cDNA was used for a PCR, and obtained PCR products were inserted into a TOPO vector, and sequenced by Sanger Sequencing (Baseclear, The Netherlands). Variable sequences for 2A2 were sequenced by Genmab (Table S6). For the IgGs produced for complement-fixation assays, sequences of the variable chain of α-Pfs230D1 IgGs were described previously (22), and ordered as synthetic products subcloned into custom-made pcDNA3.4 vectors directly upstream of Igγ1-CH1-CH3 or Igκ-CL1.

Fab and IgG purification were done in a similar manner. Plasmids coding for Fab/IgG heavy chain and light chain were co-transfected at a 1:1 molar ratio using PEI MAX^®^ transfection reagent (PolySciences) into Freestyle 293-F or 293-S cells (ThermoFisher). Cells were cultured in FreeStyle 293 Expression medium (Gibco) at 37 °C and 8% CO_2_ while shaking for seven days, after which the supernatant containing secreted recombinant protein was collected. After filtering, the supernatant was used for affinity purification using a HiTrap Kappaselect column (Cytiva) for recombinant Fab, or the HiTrap Protein G HP column (Cytiva) for recombinant IgG, pre-equilibrated in 1x PBS. In both cases, recombinant protein was eluted in 100 mM glycine (pH 2.2). Fabs were further purified by cation-exchange chromatography using a MonoS column (Cytiva) with a buffer of 20 mM sodium acetate (pH 5.6) across a 0–1.0 M potassium chloride gradient.

Single Pfs230 domains Pfs230D1 and Pfs230D10 were expressed and purified as described previously (39). Coding sequences for the double Pfs230 domain D13–14-Cterm (residue 2831–3105) were ordered and synthesized as *Drosophila melanogaster* codon-optimized sequences (Baseclear). The synthetic gene was subcloned into pExpreS2.2 plasmid (ExpreS2ion Biotechnologies), in frame with an N-terminal BiP signal peptide – His_6_ tag. The expression plasmid was used to transfect *D. melanogaster* S2 cells (ExpreS2ion Biotechnologies) to generate stable cell lines as described previously (39). Pfs230D13–14-Cterm-containing supernatant was loaded directly on a 1 ml cOmplete^™^ His-Tag Purification Column (Roche). The column was washed with PBS, and Pfs230D13–14-Cterm was eluted using PBS with 250 mM imidazole. After overnight dialysis against PBS, the sample was concentrated and further purified by size-exclusion chromatography using a Superdex200 Increase column (Cytiva), using PBS as running buffer.

### Blue Native Polyacrylamide Gel Electrophoresis

For BN-PAGE, the elution fraction from FLAG-based affinity purification, containing purified Pfs230:Pfs48/45 protein complex, was used. To test Fab binding to the complex, a tenfold molar excess of Fab fragment was added to the purified Pfs230:Pfs48/45 complex, and incubated on ice for 30 min. Protein samples were supplemented with a home-made 1x loading solution (final concentration 10% glycerol, 0.025% Coomassie Blue G-250), and loaded on a NativePAGE^™^ 3–12% Bis-Tris protein gels (ThermoFisher) following manufacturer’s protocols. NativeMark^™^ Unstained Protein Standard was used as marker. After electrophoresis, gels were either stained with Coomassie InstantBlue^®^ protein stain (Abcam) or incubated in 0.1% SDS for western blotting. For western blotting, proteins were transferred to 0.45 μm Immun-Blot PVDF membrane (BioRad) using the Trans-Blot Turbo Transfer system (BioRad). After transfer, proteins were fixed to the membrane using 8% acetic acid. Membranes were air-dried, washed in pure methanol and re-hydrated using MQ. Blocking, antibody incubation and detection were performed as described above.

### Mass spectrometry

Three batches of 40 ml heparin-treated, late-stage gametocyte cultures (iGP2^230-tag^ and iGP2 WT) of comparable gametocytaemia were harvested and lysed in parallel, as described as above for purification of the endogenous Pfs230:Pfs48/45 complex. Protein concentration in parasite lysate was determined using the PierceTM BCA Protein Assay kit. 120 μg of protein extract in 300 μl solubilization buffer (25 mM HEPES pH 7.4, 150 mM KCl, 10% glycerol, 1x PI, and 0.25% (v/v) DDM) was applied on 10 μl anti-FLAG^®^ M2 affinity resin, and incubated overnight. The resin was washed in ice-cold wash buffer (25 mM HEPES pH 7.4, 150 mM KCl, 10% glycerol, 1x PI, and 0.02% DDM) three times, and consequently washed three times in ice-cold PBS (Gibco). After harvesting, beads were resuspended in 50 μl elution buffer (100 mM Tris-HCl pH 8.0, 2 M Urea, 10 mM DTT), and incubated for 20 min at 25 °C with shaking. 50 mM iodoacetamide was added to alkylate cysteines, after which samples were kept in the dark for 10 min at 25 °C. After adding 0.25 μg sequencing grade trypsin (Promega), samples were incubated at 25 °C for 2 h with gentle shaking. Samples were spun down, supernatants were collected, and the beads were resuspended in 50 μl fresh elution buffer. After 5 min incubation on a shaker, the samples were spun down again and the supernatant combined with the previous elution fraction. Another 0.1 μg of fresh trypsin was added to the combined supernatants, which was left to digest overnight at 25 °C. The following day, samples were concentrated and purified on C18 StageTips (87). Samples were analyzed on a Obitrap Exploris 480 mass spectrometer (ThermoScientific) ran in Top20 mode (with dynamic exclusion enabled for 45 s), operated with an online Easy-nLC 1000. A gradient of buffer B (80% acetonitrile, 0.1% formic acid) was applied for 60 min. Raw data was analyzed using Maxquant software (version 2.1.4.0), and analyzed against a *Plasmodium* database (PlasmoDB, downloaded 21–10-2022). LFQ, iBAQ and match between runs were enabled, and deamidation (NQ) was added as additional variable modification. The resulting output was filtered using Perseus (version 1.0.15), removing potential contaminants, reverse hits, and proteins with less than two unique peptides or with less than three valid values in at least one group. Missing values were imputed using default settings, and a t-test was performed to identify outliers. Data was visualized using GraphPad Prism.

### Cryo-EM data collection and image processing

The Pfs230:Pfs48/45:RUPA-97:LMIV230–01:2A2:18F25:RUPA-71:RUPA-44 Fab complex was concentrated to 0.8 mg/ml, and 1.8 μl sample was deposited on homemade holey gold grids (88) that were glow-discharged in air for 15 s. Excess sample was blotted away for 1.7 s using a Leica EM GP2 Automatic Plunge freezer at 4 °C and 90% humidity, and plunge-frozen in liquid ethane. Data were collected on a Thermo Fisher Scientific Titan Krios G3 equipped with a Selectris X energy filter (slit width: 10 eV) and Falcon 4i camera, operated at 300 kV and automated with the EPU software. Data was collected at a magnification of 130,000× with a calibrated pixel size of 0.93 Å. Exposures were collected for 6.5 s as movies with a camera exposure rate of ~7.11 or ∼7.15 e^−^ per pixel per s, and total exposure of ~50 or ~53.7 electrons/Å^2^ and recorded in Electron Event Representation mode (89). Before processing, movies were fractionated into 40 frames. A total of 8193, 1572, and 6885 raw movies were obtained at 0^o^, 35^o^, and 40^o^ tilt, respectively.

The collected cryo-EM data was processed using CryoSPARC v4.6.0 (90). The movies from the 35° tilted, 40° tilted, and untilted data collections were corrected for motion in patches, and contrast transfer function (CTF) parameters were estimated in patches. Resulting micrographs were curated using thresholds for average defocus values (4000–35,000 Å), CTF fit resolution (2.0–5.0 Å), and relative ice thickness (0.95–1.10). An *in silico* model of the Pfs230:Pfs48/45:RUPA-97:LMIV230–01:2A2:18F25:RUPA-71:RUPA-44 complex was built from AF models and arranged based on views seen from 2D classes. The resulting model was used to generate particle templates corresponding to 40 evenly distributed projections which were low-pass filtered to 20 Å to avoid model bias. Initially, particle images were selected from a subset of 2000 curated micrographs, which were then extracted in 450x450 pixel boxes and subjected to 2D classification. Classes where the average showed clear views of the complex were used to train a Topaz particle selection model (91), with subsequent 2D classification steps used to clean the dataset of selected particle images. To focus on the various subsections of the full complex, 2D class average images showing the appropriate domains were used as templates to select more particles from the full dataset of 12,192 curated micrographs. Further Topaz models were trained on specific regions (Pfs230D1–6:RUPA-97:LMIV230–01:2A2, Pfs230D9–14:Pfs48/45:RUPA-71:RUPA-44) of the full complex, after which particles images were selected and extracted with the trained models. Initial junk particle image removal was performed with multiple rounds of 2D classification, followed by *ab initio* 3D reconstruction. Non-particle images and poorly behaved particle images were removed with a combination of heterogeneous refinement, 3D classification, and 2D classification. Selected particle images were used to generate 3D maps with reconstructions in non-uniform refinement (92) jobs without enforced symmetry (C1). The resulting maps were further improved by local refinement and post-processing with DeepEMhancer (93). Heterogeneity for 2A2 Fab binding could be distinctly observed, such that two maps of Pfs230D1–6:LMIV230–01:RUPA-97 were obtained: with and without 2A2 Fab. A detailed processing flow path and experimental map details are available in the supplementary document (Figure S2, S3A-S3D).

For the Pfs230D7–8:18F25 map, particle images from a subset of 500 micrographs were used from one set of collections of the untilted (2003), 35° tilted (337), and 40° tilted (2866) micrographs for blob picking after motion correction, CTF estimation, and curation of the micrographs (average defocus = 1000–30,000 Å; CTF fit resolution < 5 Å; relative ice thickness = 0.954–1.1; astigmatism < 4000 Å). The 101,225 particle stack was cleaned using multiple rounds of 2D classification, ab initio reconstruction, and homogeneous refinement (4.89 Å). The rebalance orientations job was used, followed by ab initio reconstruction and non-uniform refinement (4.19 Å). A map containing the interface between Pfs230 and 18F25 Fab was solved by cleaning the particle stack with 2D classification, followed by ab initio reconstruction and homogeneous refinement (10.03 Å). This map, along with the 4.19 Å map and a junk class generated with ab initio reconstruction were used as input for heterogeneous refinement, resulting in a class containing 18F25 with 35,162 particles. The resulting particle stack was cleaned with multiple rounds of 2D classification, with the final particle stack used for Topaz training on denoised micrographs. Ab initio reconstruction with five classes was run using the resulting 100,481 particle stack, followed by heterogeneous refinement. The particle stack from the class containing 18F25 was cleaned using 2D classification, then used again for Topaz training on all micrographs (6372 untilted, 1125 35° tilted, 2866 40° tilted). To ensure the particles were centered on 18F25, ab initio reconstruction, followed by non-uniform refinement was run with the resulting 366,876 particle stack at a box size of 600 pix and Fourier crop of 300 pix (5.79 Å), followed by centering of the particles using volume alignment tools and re-extraction of the particles to 300 pix boxes. 2D classification without re-centering, 2 class ab initio reconstruction, and heterogeneous refinement were used to obtain a clean 216,684 particle stack. A further three rounds of 2D classification and two rounds of non-uniform refinement and local refinement resulted in a 3.88 Å map using 106,349 particles. Post-processing was performed in EMReady to prepare the map for model building. A detailed processing workflow and experimental map details are available in the supplementary document (Figure S2, S3E).

### X-Ray Crystallography and Structural Determination

Fractions containing purified 2A2, 18F25, and RUPA-71 Fab following cationic exchange chromatography were pooled and concentrated at 16.8 mg/mL, 27.3 mg/mL, and 10 mg/ml, respectively in preparation for crystallization trials. RUPA-71 Fab was mixed in a 1:1 ratio with reservoir conditions from a JCSG Top96 screen (0.2 μL + 0.2 μL) using the sitting drop vapor diffusion method. RUPA-71 crystals then formed in a drop containing 0.2 M (NH4)_2_SO_4_, 0.1 M MES 6.5 pH, and 30 %w/v PEG MME 5K. For the 2A2 and 18F25 Fab crystallization trials, reservoir conditions from a JCSG Top96 screen and Fab were mixed at a 1:1 volumetric ratio (0.3 μL + 0.3 μL) resulting in 0.6 μL drops. Approximately 48 h after setting drops at room temperature, 2A2 crystals were obtained from a drop containing reservoir solution: 30% (w/v) PEG 4K. 18F25 crystals were obtained from a drop containing reservoir solution: 0.2 M Na2SO4 and 20% (w/v) PEG 3350. The 2A2 and 18F25 crystals were cryo-protected with 25% glycerol (v/v) and 15% polyethylene glycol 200 (PEG200), respectively. Crystals of all three samples were flash-frozen in liquid nitrogen. Data collection on 2A2 and 18F25 was performed at the 17-ID-1 beamline at the Brookhaven National Laboratory Synchrotron Light Source. RUPA-71 data collection occurred at the CMCF-ID beamline at the Canadian Light Source. Datasets were processed using autoproc (94) and xds (95). A molecular replacement solution was obtained using PhaserMR (96). Model building and refinement was performed using Coot (97) and phenix.refine (98). Inter- and intra-molecular contacts were determined using PISA (99) and manual inspection. Structural figures were generated using UCSF ChimeraX (100).

### Cryo-EM model building

Starting structural models were obtained by manually fitting previously determined structural models (PDB: 8U1P, Pfs48/45-D1D2; 7UXL, Pfs48/45-D3:RUPA-44; 7UVQ, Pfs230-D1:RUPA-97; and 7UFW, Pfs230-D1D2:LMIV230–01), novel crystal structures (Fabs of 18F25, RUPA-71, and 2A2), and models generated by AlphaFold2 (101) and AlphaFold3 (64) into the experimentally determined maps. Structural refinements were performed using Isolde (102), PyRosetta (103), and PHENIX (104); models were manually checked and improved with Coot (97). Images were generated using ChimeraX (100). Access to all software was supported through SBGrid (105).

### Single nucleotide polymorphism detection

Single nucleotide polymorphisms were obtained from the MalariaGEN Catalogue of Genetic Variation in Pf version 7 (54). Genotype calls for chromosomes 2 and 13 (Pf3D7_02_v3 and Pf3D7_13_v3, respectively) were downloaded (https://ngs.sanger.ac.uk/production/malaria/Resource/34/Pf7_vcf/). Bcftools (106) was used to subset calls and calculate the allele frequencies between nucleotide positions 369,351 and 380,156(+) on chromosome 2 that coincide with Pfs230 and between nucleotide positions 1,875,452 and 1,878,087(-) on chromosome 13 that coincide with Pfs48/45. SNPs calls below MalariaGEN’s quality filter (Low_VQSLOD) were removed before identification of missense variants.

### Gamete binding assay with flow cytometry

Heparin treated stage V gametocytes (day 13 – 16 post induction) were collected and resuspended in FCS with half the volume of the original culture volume. Gamete maturation was induced for 30 min at room temperature while shaking, after which samples were centrifuged at 2,000xg at 4 °C. The pellet was resuspended in PBS and placed on a layer of 12.4% Nycodenz (Serumwerk Bernburg) and centrifuged for 30 min at 3,500xg without break at 4°C. The top layer containing female gametes was harvested and washed in PBS before continuing.

50,000 female gametes per well were deposited in a V-bottom non-treated 96-well plate (Costar). All gamete incubations were carried out at RT in PBS supplemented with 2% Fetal Calf Serum (FCS, Gibco) and 0.02% sodium azide, and washed in ice-cold PBS. All secondary antibody incubations included eBioscience^™^ Fixable Viability Dye eFluor^™^ 780 (Invitrogen, 1:1000). For the Pfs230 domain binding experiment (Figure S6A), gametes were incubated for 1 h with recombinant proteins and washed in PBS. Afterwards, gametes were incubated with mouse anti-his tag antibody (Sigma-Aldrich, A7058, 1:200) for 1 h, washed, and stained with chicken-anti-mouse 488 secondary antibodies (Invitrogen, A21200, 1:200) for 30 min. For the antibody gamete binding experiment (Figure S6H, Figure 5G), gametes were incubated with 1 μg/ml antibody for 1 h, washed, and stained with goat-anti-human 488 secondary antibody (Invitrogen, A11013, 1:200). For the Fab competition experiment (Figure S7C), gametes were incubated for 1 h with Fabs. After washing in PBS, gametes were stained with 18F25 antibody labeled with LYNX Rapid Plus DyLight488 Antibody Conjugation Kit (Bio-Rad, 5 μg/ml). Finally, for complement deposition assay, gametes were incubated with 1 μg/ml antibody for 1 h and washed. Gametes were then incubated with 10% Normal Human Serum (Sanquin, The Netherlands) and incubated for 45 min at RT. After washing, parasites were first stained with polyclonal goat anti-human C1q (CompTech, 1:5,000) or anti-human C3 (CompTech, 1:10,000), washed, and finally stained with donkey-anti-goat 488 secondary antibody (Invitrogen, A11055, 1:200). After staining, gametes were resuspended in PBS and fluorescence was measured for approximately 2,000 gametes with the Gallios^™^ 10-color system (Beckman Coulter), and analyzed with FlowJo (BD, version v10.10.0). For all gamete binding experiments only live gametes were analyzed, except for the complement deposition assays for which both live and dead gametes were included in the analysis (gating strategy in Fig S6J). Statistical analyses were performed using GraphPad Prism (version 10.4.1), and are described in the figure legends.

## Supplementary Material

1

## Figures and Tables

**Table 1.** CryoEM data collection and refinement statistics.

Data Collection	Pfs230D1-C: RUPA-37:LMIV1:2A2	Pfs230D7–8:18F25	Pfs230D3–14:Pfs48/45	Pfs230D13–14:Pfs48/45: RUPA-44:RUPA-71
Electron microscope	Titan Krios G3	Titan Krios G3	Titan Krios G3	Titan Krios G3
Camera	Falcon 4i Direct Electron Detector, SelectrisX energy filter	Falcon 4i Direct Electron Detector, SelectrisX energy filter	Falcon 4i Direct Electron Detector, SelectrisX energy filter	Falcon 4i Direct Electron Detector, SelectrisX energy filter
Voltage (kV)	300	300	300	300
Nominal magnification	130,000	130,000	130,000	130,000
Calibrated physical pixel size (Å)	0.93	0.93	0.93	0.93
Total exposure (e^-^/Å^2^)	50–53	50–53	50–53	50–53
Number of frames	30	30	30	30
***Image Processing***				
Motion correction software	cryoSPARC v4	cryoSPARC v4	cryoSPARC v4	cryoSPARC v4
CTF estimation software	cryoSPARC v4	cryoSPARC v4	cryoSPARC v4	cryoSPARC v4
Particle selection software	cryoSPARC v4	cryoSPARC v4	cryoSPARC v4	cryoSPARC v4
3D map classification and refinement software	cryoSPARC v4	cryoSPARC v4	cryoSPARC v4	cryoSPARC v4
Micrographs used	12,192	10,363	12,192	12,192
Particle images selected	1,167,622	237,721	1,942,561	1,942,561
Global resolution (Å)	3.6	3.8	4.7	3.4
Particle images contributing to final maps	70,113	165,992	138,915	146,814
***Model Building***				
Modeling software	Coot, phenix.real_space_refine, ISOLDE, Rosetta	Coot, phenix.real_space_refine, ISOLDE, Rosetta	Coot, phenix.real_space_refine, ISOLDE, Rosetta	Coot, phenix.real_space_refine, ISOLDE, Rosetta
Number of residues built	2430	546	1476	1156
RMS (bonds)	0.002	0.002	0.002	0.003
RMS (angles)	0.582	0.629	0.534	0.550
Ramachandran outliers (%)	0.04	0	0	0
Ramachandran allowed (%)	4.0	5.8	5.1	6.0
Ramachandran favoured (%)	96.0	94.2	94.9	94
Rotamer outliers (%)	0.3	0	0.2	1.3
Clashscore	6.3	5.2	4.3	10.3
MolProbity score	1.63	1.67	1.65	2.03
EMRinger Score	1.14	0.60	0.83	2.26
PDB, EMDB codes	9N5H, EMD-48921	9N5O, EMD-48941	9N5K, EMD-48924	9N5I, EMD-48922
