# Supplementary material for: Structure of endogenous Pfs230:Pfs48/45 in complex with potent malaria transmission-blocking antibodies
Source: bioRxiv. 2025 Jun 15:2025.02.14.638310. Originally published 2025 Feb 15. Preprint. [Version 2] doi: 10.1101/2025.02.14.638310 (PMC11844449; doi:10.1101/2025.02.14.638310)
Supplement: 1 [file NIHPP2025.02.14.638310V2-supplement-1.pdf]

bioRxiv preprint doi: https://doi.org/10.1101/2025.02.14.638310; this version posted June 15, 2025. The copyright holder for this preprint (which was not certified by peer review) is the author/funder, who has granted bioRxiv a license to display the preprint in perpetuity. It is made available under aCC-BY 4.0 International license.

SUPPLEMENTARY FIGURES

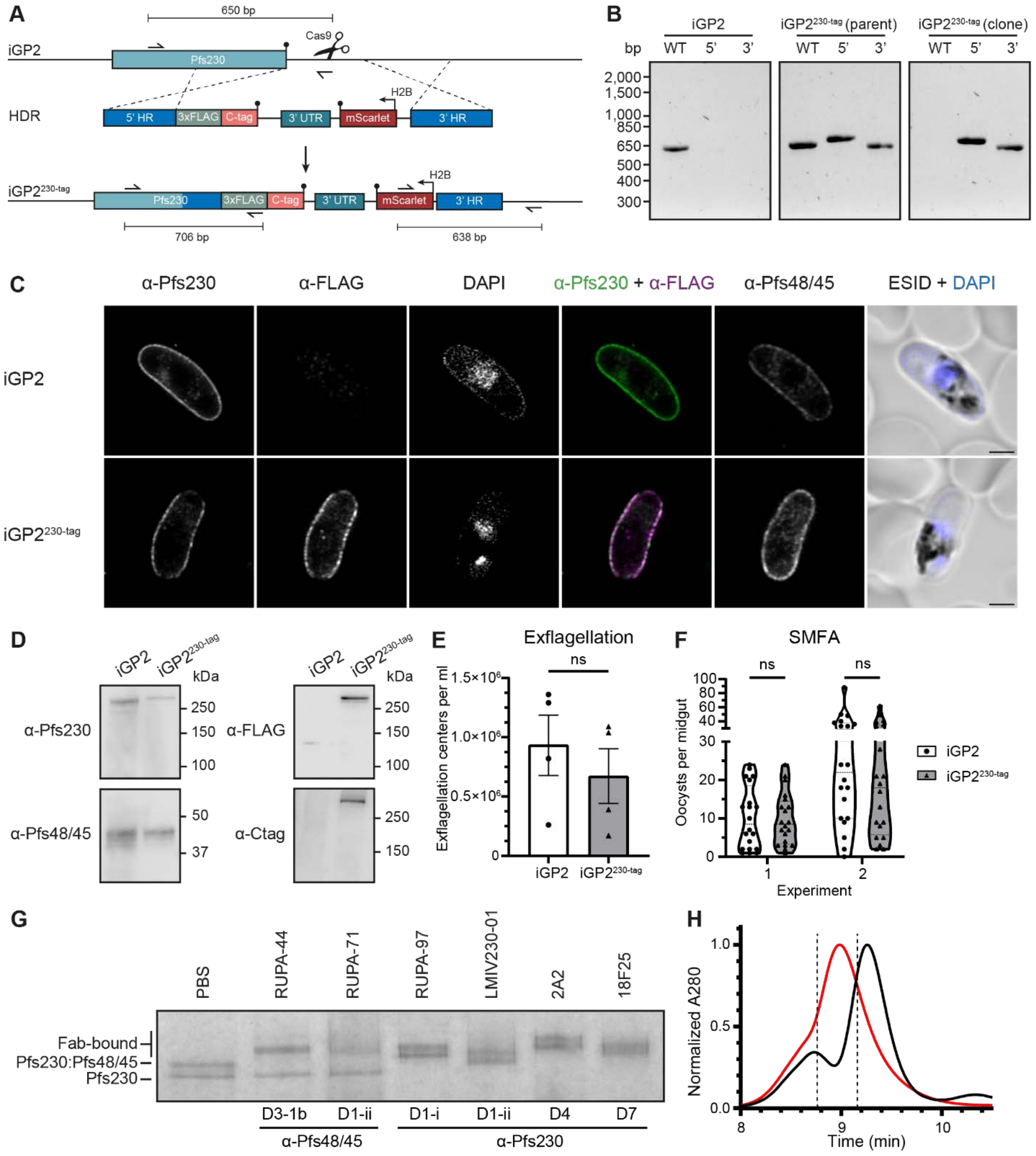

Figure S1: The iGP2$^{230\text{-tag}}$ parasite line allows for purification of endogenous Pfs230:Pfs48/45 complex recognized by transmission-blocking antibodies. (A) Schematic overview of the genomic modification to obtain the iGP2$^{230\text{-tag}}$ parasite line. HDR = Homology Directed Repair, HR = homology region, 3xFLAG = triple FLAG tag, C-tag = EPEA* tag, 3'UTR = bidirectional 3'untranslated region of PBANKA_142660, H2B = promoter of Pf3D7_1105100. **(B)** Genomic integration PCR to confirm 5' and 3' integration and the absence of wildtype parasites. Primers and expected PCR product are indicated in (A). **(C)** Immunofluorescence microscopy images of paraformaldehyde/glutaraldehyde-fixed and permeabilized iGP2 wildtype and iGP2$^{230\text{-tag}}$ stage V gametocytes. Parasites were stained for Pfs230 (green,

bioRxiv preprint doi: https://doi.org/10.1101/2025.02.14.638310; this version posted June 15, 2025. The copyright holder for this preprint (which was not certified by peer review) is the author/funder, who has granted bioRxiv a license to display the preprint in perpetuity. It is made available under aCC-BY 4.0 International license.

RUPA-55), FLAG-tag (magenta, M2), and Pfs48/45 (45.1), and DAPI (blue). Scalebar represents 5 µm. ESID = Electronically Switchable Illumination and Detection brightfield image. **(D)** Western blot analysis of iGP2 and iGP2$^{230\text{-}tag}$ to confirm FLAG-tag and C-tag integration. **(E)** Exflagellation of stage V iGP2 (white) and iGP2$^{230\text{-}tag}$ (gray) gametocytes. Bars represent mean ± standard deviation of number of exflagellation centers per ml from four independent cultures (dots). No statistical significance was found using an unpaired t-test. **(F)** Transmission of iGP2 (white) and iGP2$^{230\text{-}tag}$ (gray) to mosquitoes in two independent standard membrane feeding experiments. Dots represent the number of oocysts per midgut *Anopheles stephensi* mosquitoes (n=20 per experiment) in two independent standard membrane feeding assays using iGP2 (white) and iGP2$^{230\text{-}tag}$ (gray) parasites. Statistical analysis was done using a Mann-Whitney test with Holm-Šídák's multiple comparisons test (α = 0.05). ns = not significant. **(G)** Coomassie blue stained Blue Native PAGE gel of purified Pfs230:Pfs48/45 (and free Pfs230) incubated with molar excess of the following individual Fab fragments: RUPA-44, RUPA-71, RUP-97, LMIV230-01, 2A2, or 18F25. Epitopes are defined in Figure 1A. **(H)** Normalized absorption at 280 nm during high-pressure liquid size-exclusion chromatography of Pfs230:Pfs48/45 (black) or Pfs230-Pfs48/45:6Fab (red) complexes. Dotted lines indicate fractions of eluted Pfs230:Pfs48/45:6Fab that were used for cryo-electron microscopy. *Related to Figure 1.*

bioRxiv preprint doi: https://doi.org/10.1101/2025.02.14.638310; this version posted June 15, 2025. The copyright holder for this preprint (which was not certified by peer review) is the author/funder, who has granted bioRxiv a license to display the preprint in perpetuity. It is made available under aCC-BY 4.0 International license.

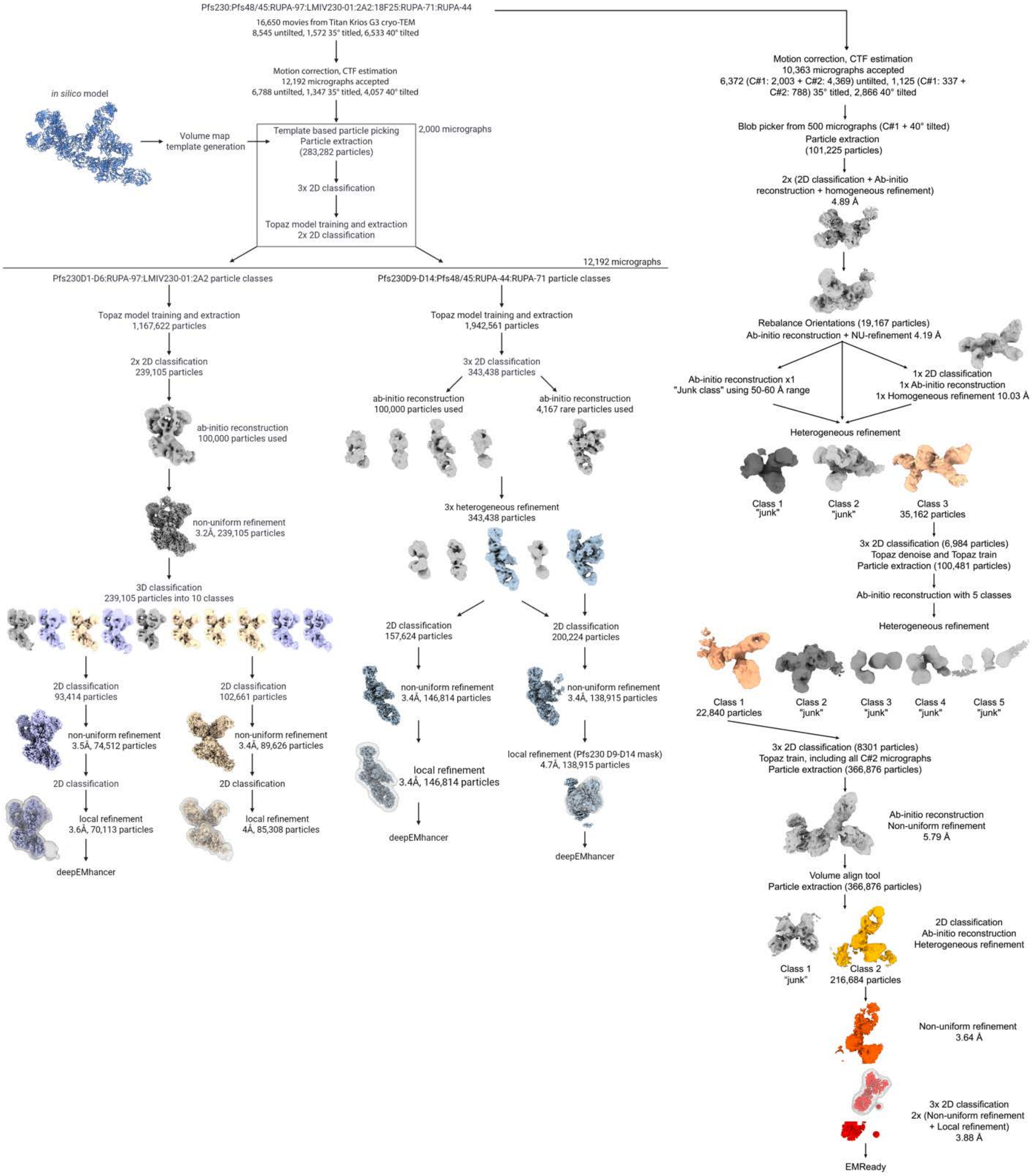

Figure S2: Cryo-EM processing workflow. Shown from left to right are the maps of Pfs230D1-6:LMIV230-01:RUPA-97:2A2, Pfs230D1-6:LMIV230-01:RUPA-97, Pfs230D13-14:Pfs48/45:RUPA-44:RUPA-71, the focused refinement for Pfs230D9-14:Pfs48/45, and the Pfs230D7-8:18F25 map. Abbreviations for the two data collection sessions are abbreviated in the Pfs230D7-8:18F25 workflow (C#1: collection C#2: collection 2). *Related to Figure 1.*

bioRxiv preprint doi: https://doi.org/10.1101/2025.02.14.638310; this version posted June 15, 2025. The copyright holder for this preprint (which was not certified by peer review) is the author/funder, who has granted bioRxiv a license to display the preprint in perpetuity. It is made available under aCC-BY 4.0 International license.

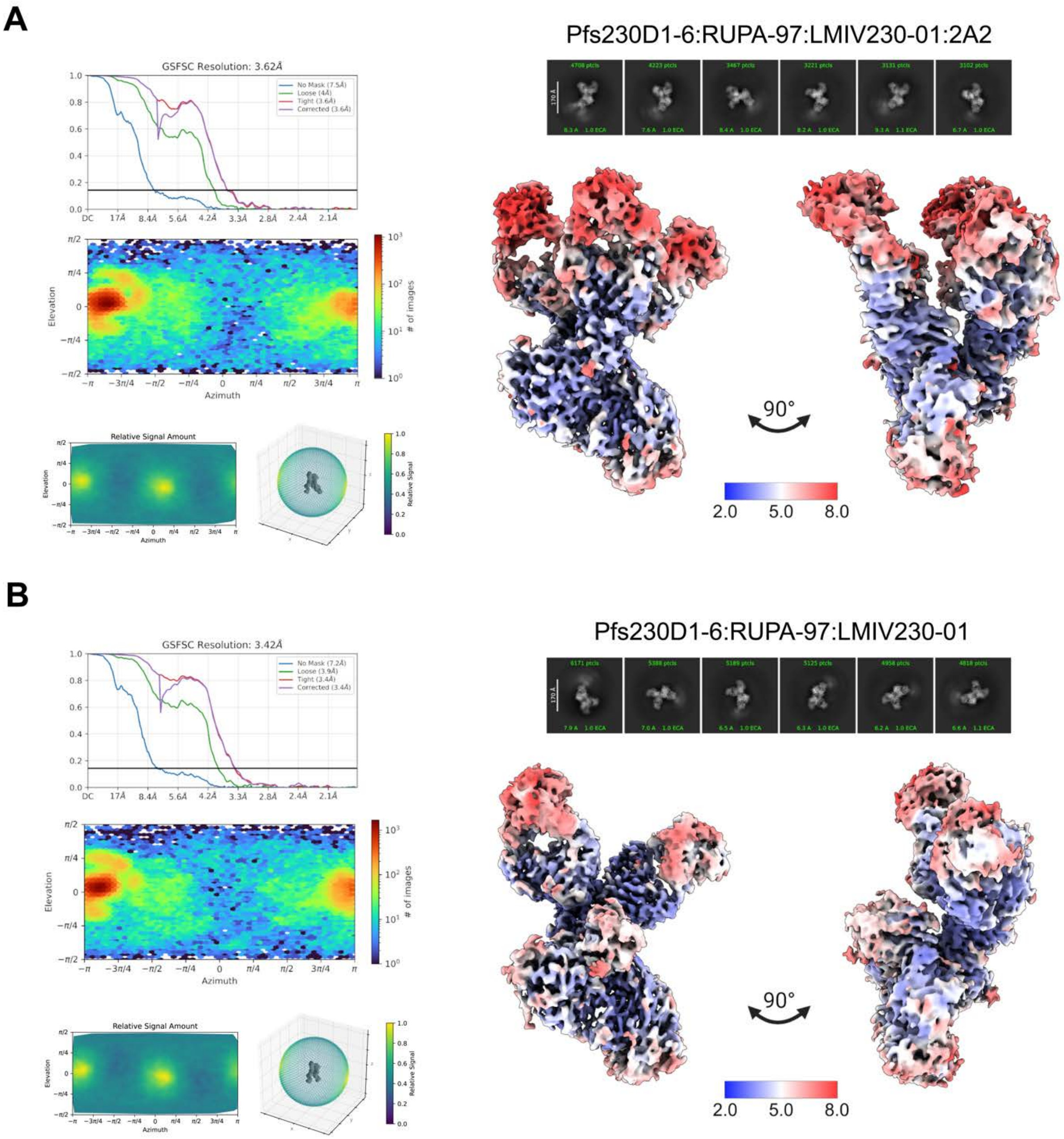

bioRxiv preprint doi: https://doi.org/10.1101/2025.02.14.638310; this version posted June 15, 2025. The copyright holder for this preprint (which was not certified by peer review) is the author/funder, who has granted bioRxiv a license to display the preprint in perpetuity. It is made available under aCC-BY 4.0 International license.

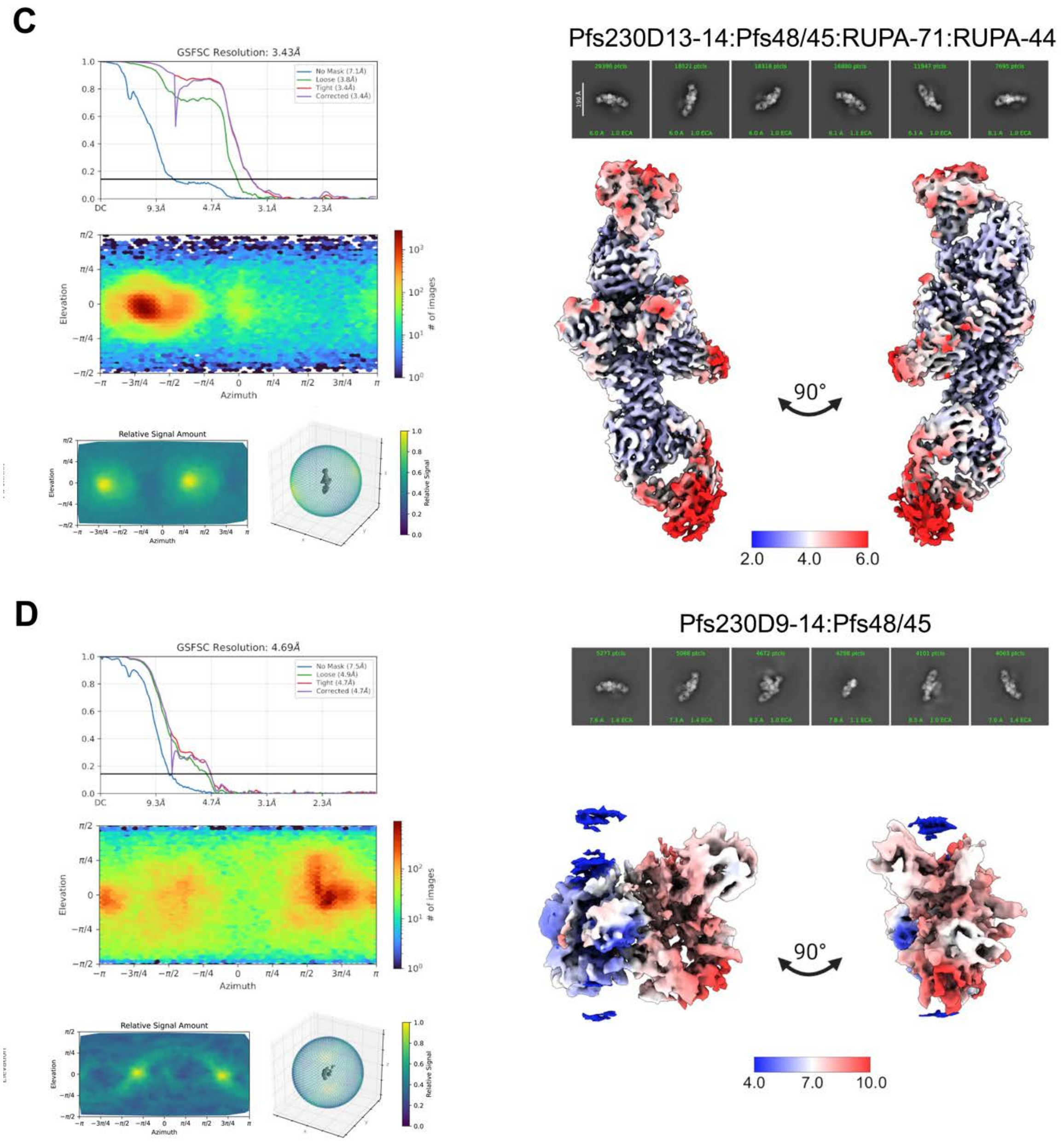

bioRxiv preprint doi: https://doi.org/10.1101/2025.02.14.638310; this version posted June 15, 2025. The copyright holder for this preprint (which was not certified by peer review) is the author/funder, who has granted bioRxiv a license to display the preprint in perpetuity. It is made available under aCC-BY 4.0 International license.

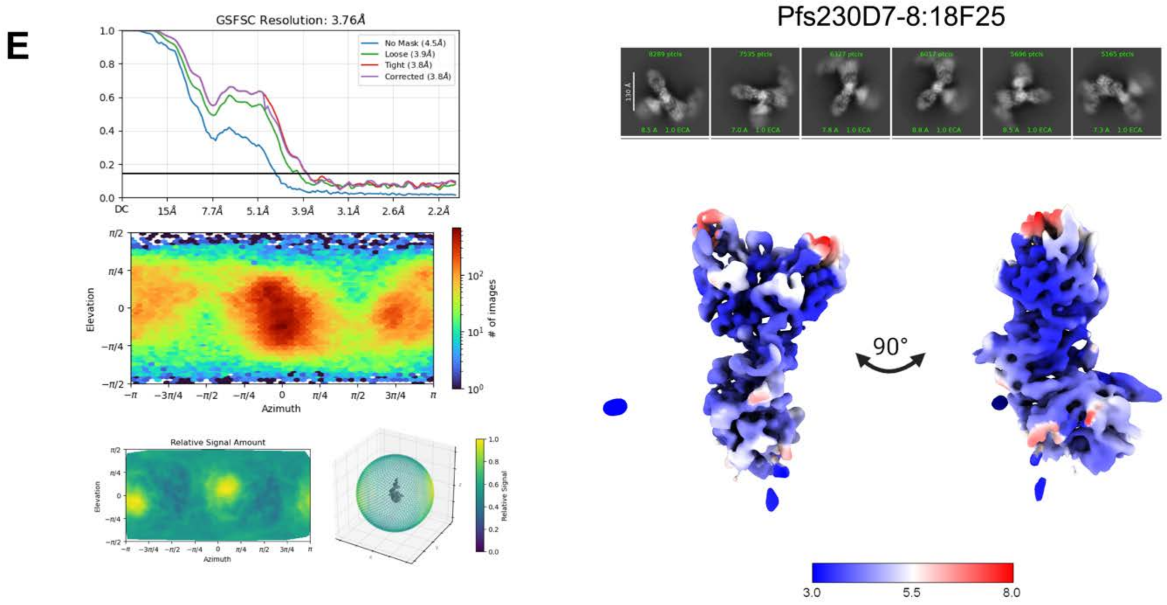

Figure S3: Cryo-EM map validation data. **(A-E)** The structures of (A) Pfs230D1-6:RUPA-97:LMIV230-01:2A2; (B) Pfs230D1-6:RUPA-97:LMIV230-01; (C) Pfs230D13-14:Pfs48/45:RUPA-44:RUPA-71; (D) focused refinement for Pfs230D9-14:Pfs48/45; and (E) Pfs230D7-8:18F25. The Fourier shell correlation curves following a gold-standard refinement with correction for the effects of masking for varying masks, the viewing direction particle distribution, and orientation diagnostic plots are shown (left panel). Additionally, representative 2D classes and cryo-EM maps coloured by local resolution are shown for each processed map (right panel). *Related to Figure 1.*

bioRxiv preprint doi: https://doi.org/10.1101/2025.02.14.638310; this version posted June 15, 2025. The copyright holder for this preprint (which was not certified by peer review) is the author/funder, who has granted bioRxiv a license to display the preprint in perpetuity. It is made available under aCC-BY 4.0 International license.

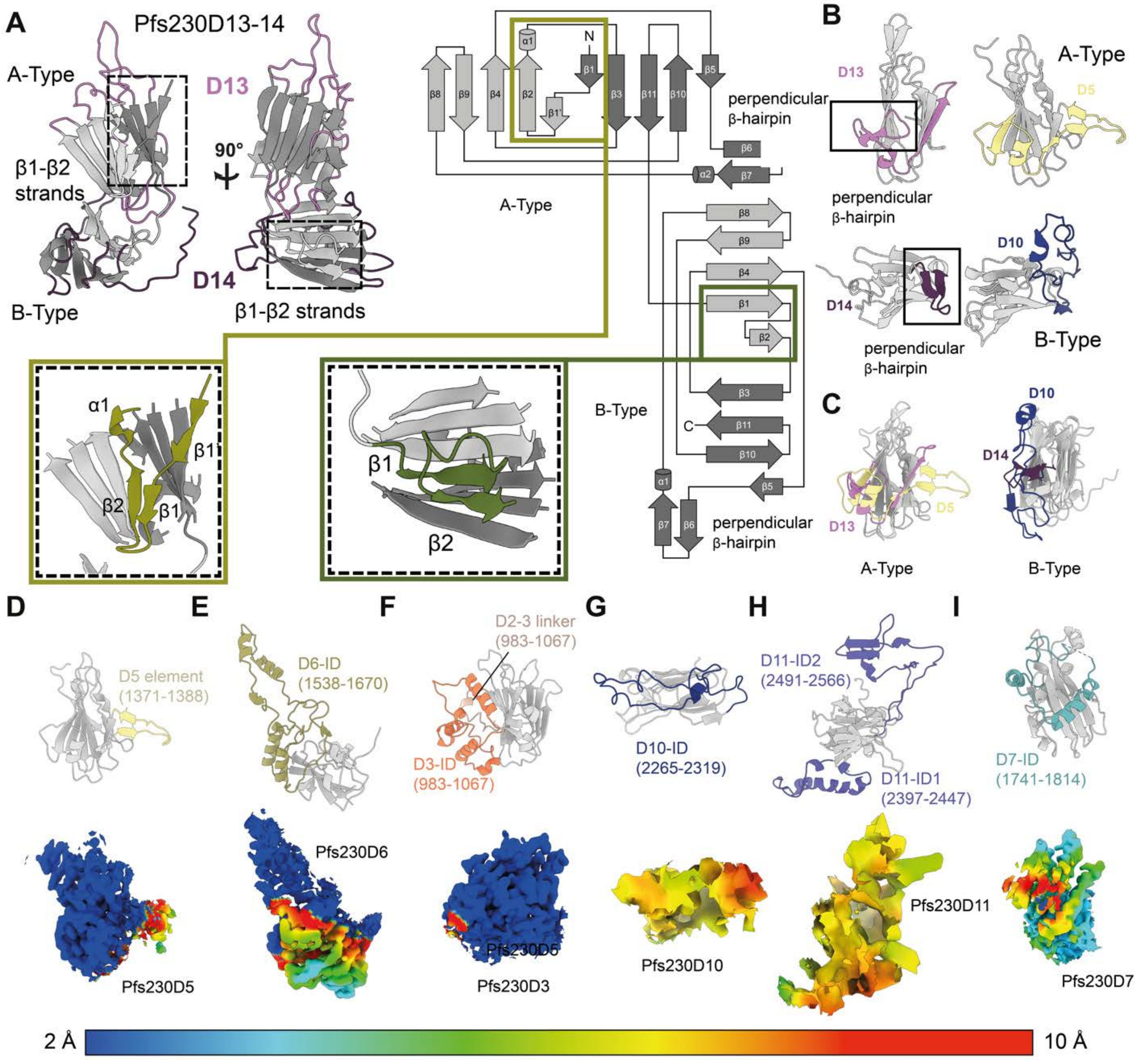

Figure S4: Structural features of Pfs230 6-Cys domains and Pfs230 domain clustering. (A) Pfs230D13-14 is shown as prototypical tandem A- and B-type 6-Cys domains of Pfs230. Loops of D13 and D14 are coloured according to the colouring scheme in Figure 1. The first β-sheet of the A-type and B-type domains is coloured distinctly (light gray) from the second β-sheet (gray). The distinguishing features of the A- and B-type domains (β1 to β2 strands) are indicated through the dashed box. Secondary structure topology of the two 6-Cys domains is shown with distinguishing features of A- (olive) and B-type (green) domains (β1 to β2 strands) indicated (inset) with a 3D representation of these differences shown. **(B-C)** Comparison of prototypical tandem 6-Cys domains (D13 and D14) to D5 and D10 respectively, (B) side by side and (C) overlaid, highlighting the structural differences of D5 and D10 at the perpendicular β-hairpin region. The regions that are structurally distinct are coloured according to the scheme in Figure 1. **(D-I)** Pfs230 domains that harbour IDs (Pfs230D3, D6, D7, D10, and D11) or structural elements contributing to ID clustering (Pfs230D3 and D5) are shown as ribbon representations with the cryoEM map corresponding to that portion of the model being shown underneath (shown at 5 Å radius around the models) and coloured according to local resolution (scale bar below). Non-canonical elements and IDs are coloured according to the colouring scheme specified in Figure 2. *Related to Figure 2.*

bioRxiv preprint doi: https://doi.org/10.1101/2025.02.14.638310; this version posted June 15, 2025. The copyright holder for this preprint (which was not certified by peer review) is the author/funder, who has granted bioRxiv a license to display the preprint in perpetuity. It is made available under aCC-BY 4.0 International license.

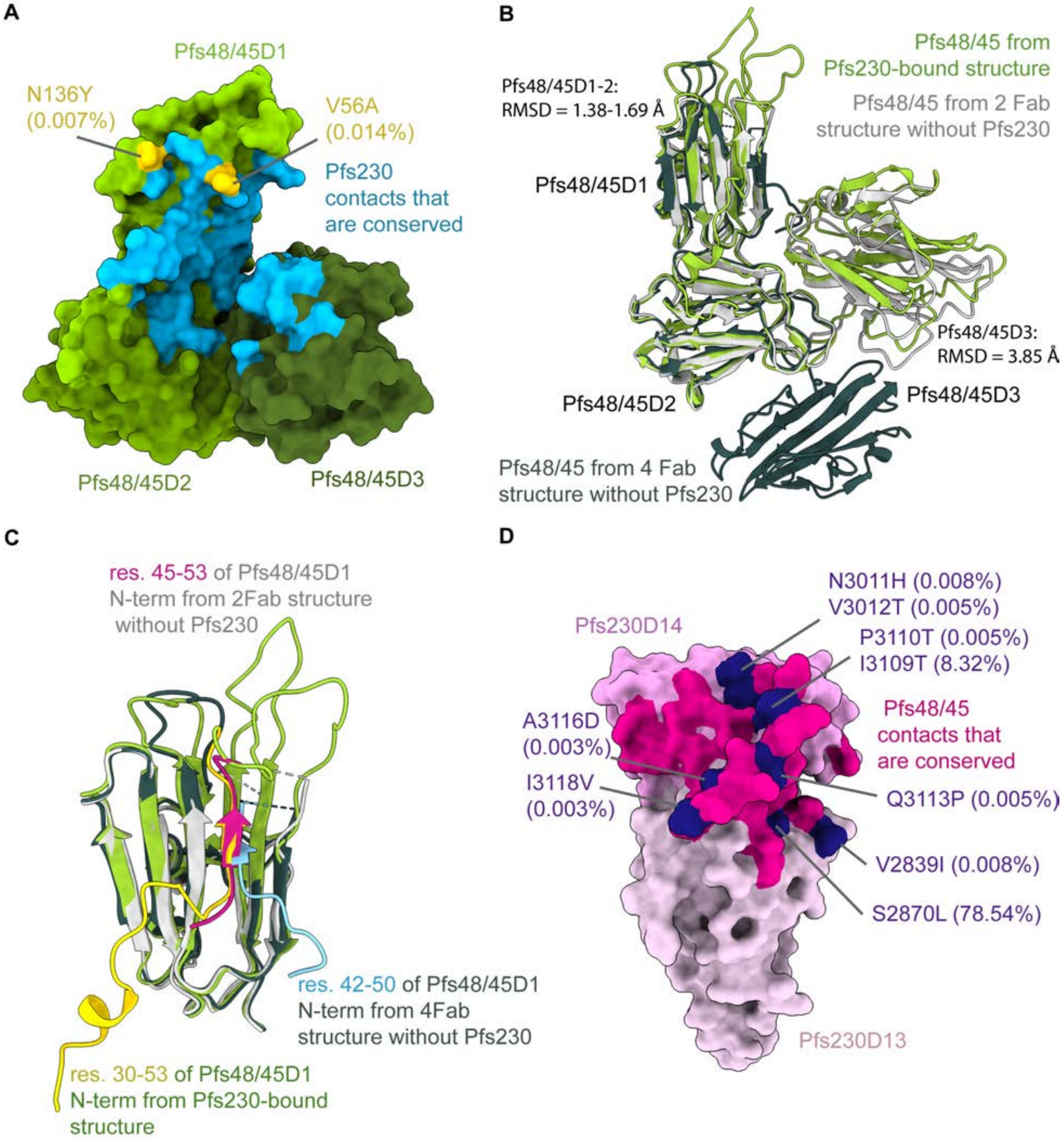

Figure S5: Comparison of Pfs48/45 conformation across determined structures and sequence conservation of Pfs230:Pfs48/45 binding site. (A) Structure of Pfs48/45 depicted as surface (yellow green, olive drab, and dark olive drab for Pfs48/45D1-3, respectively) with the Pfs230 binding site shown in sky blue and single nucleotide polymorphisms present within the epitope shown in gold. **(B)** Overlay of Pfs48/45 structures from Pfs48/45-Pfs230-6Fab complex (yellow green), Pfs48/45-4Fab complex (dark slate grey, PDB ID: 8U1P), and Pfs48/45-2 Fab complex (light grey, PDB ID: 7ZXF) aligned to Pfs48/45D1-2. **(C)** Overlay of N-terminal region of Pfs48/45-Pfs230-6Fab complex (green, yellow N-term), Pfs48/45-4Fab complex (dark slate grey, light blue N-term, PDB ID: 8U1P), and Pfs48/45-2 Fab complex (light grey, dark violet N-term, PDB ID: 7ZXF) aligned to Pfs48/45D1. **(D)** Pfs230D13 (thistle) and D14 (plum) shown as surface with the Pfs48/45 binding site in dark pink and SNPs in indigo. *Related to Figure 3.*

bioRxiv preprint doi: https://doi.org/10.1101/2025.02.14.638310; this version posted June 15, 2025. The copyright holder for this preprint (which was not certified by peer review) is the author/funder, who has granted bioRxiv a license to display the preprint in perpetuity. It is made available under aCC-BY 4.0 International license.

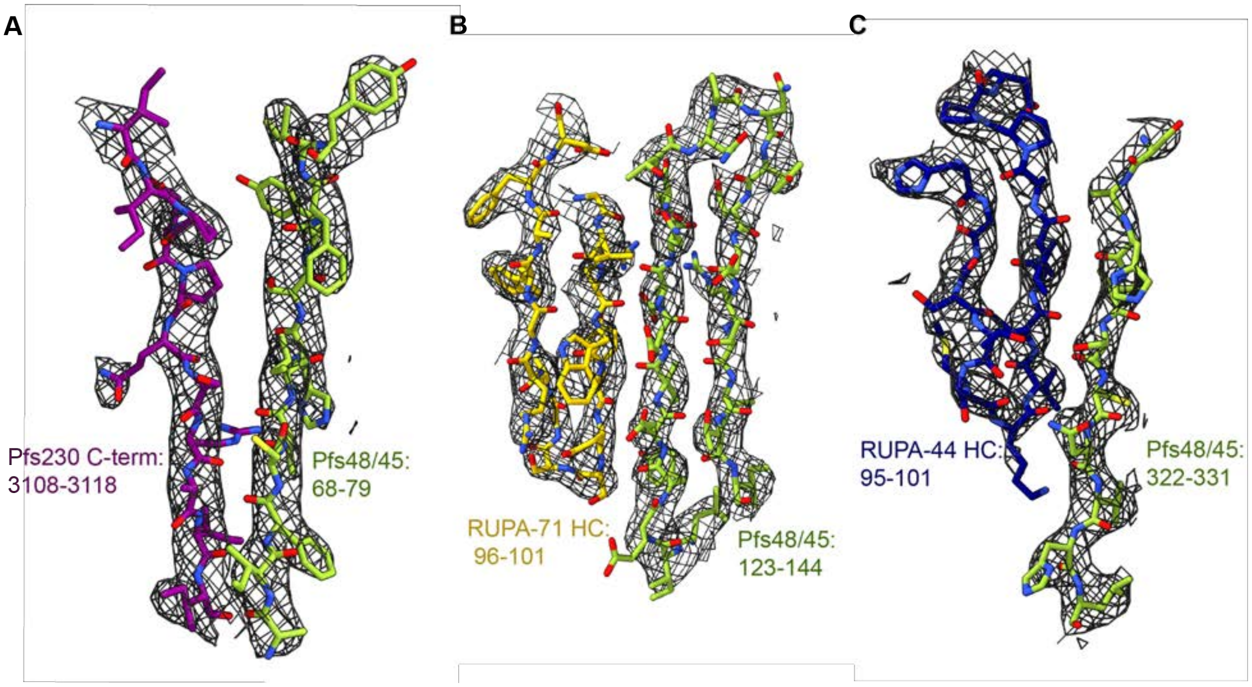

Figure S6: Cryo-EM map density around Pfs230:Pfs48/45:RUPA-71:RUPA-44 model. (A-D) Experimental map in grey mesh around key regions, including (A) the Pfs230 C-terminus and several of its Pfs48/45 contact residues, (B) residues at the Pfs48/45D1-RUPA-71 interface, and (C) residues at the Pfs48/45D3-RUPA-44 interface. Pfs48/45 (green), Pfs230D13-14 (purple), RUPA-71 (gold), and RUPA-44 (dark blue) are depicted as sticks. *Related to Figure 3-4.*

bioRxiv preprint doi: https://doi.org/10.1101/2025.02.14.638310; this version posted June 15, 2025. The copyright holder for this preprint (which was not certified by peer review) is the author/funder, who has granted bioRxiv a license to display the preprint in perpetuity. It is made available under aCC-BY 4.0 International license.

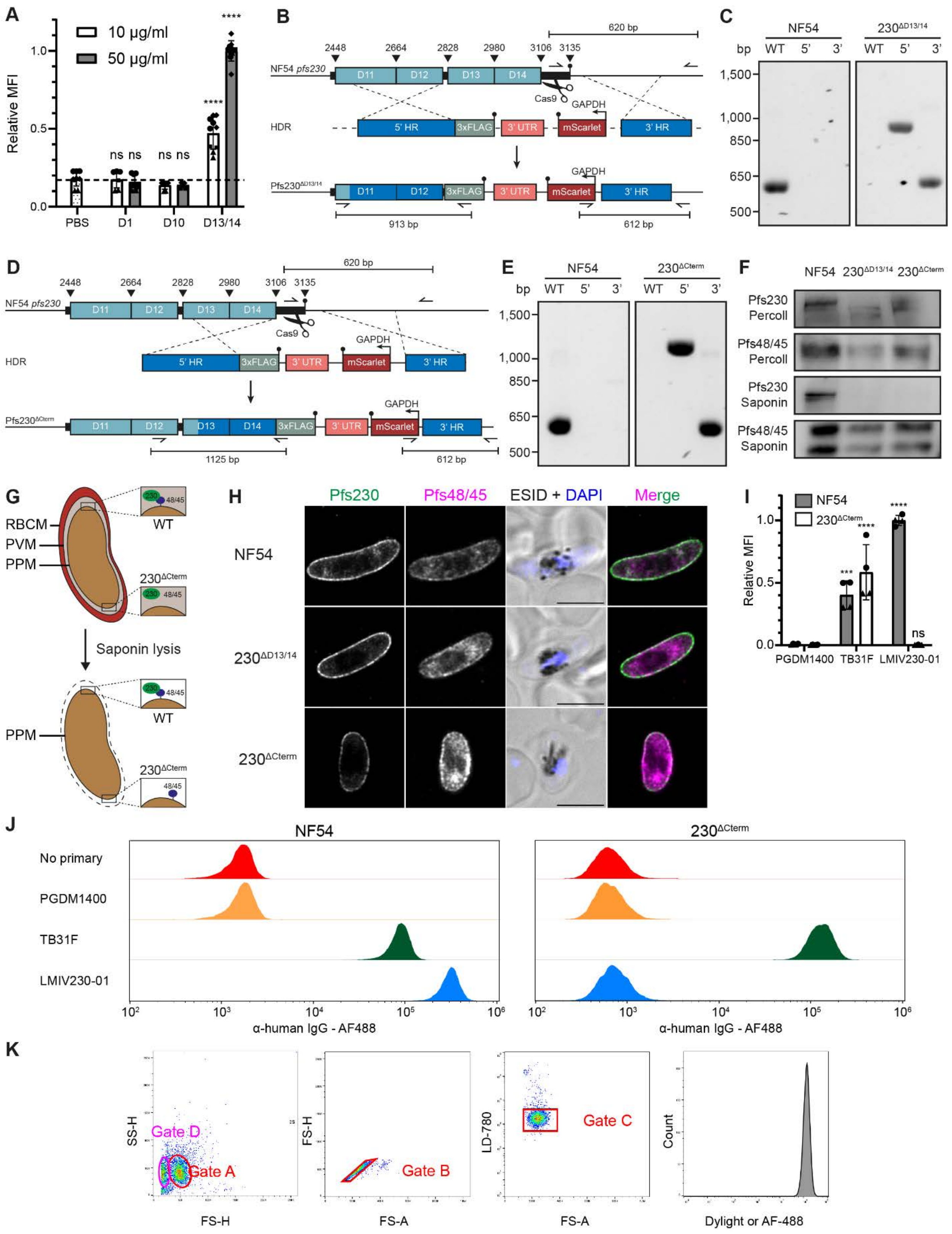

Figure S7: Pfs230:Pfs48/45 interactions are mediated by the C-terminal region Pfs230D13-14-Cterm *in vivo*. (A) Female gamete binding assay with recombinantly purified Pfs230D1, Pfs230D10 and Pfs230D13/14-Cterm at 10

bioRxiv preprint doi: https://doi.org/10.1101/2025.02.14.638310; this version posted June 15, 2025. The copyright holder for this preprint (which was not certified by peer review) is the author/funder, who has granted bioRxiv a license to display the preprint in perpetuity. It is made available under aCC-BY 4.0 International license.

µg/ml (white bar) or 50 µg/ml (gray bar). Bars show mean MFI (Mean Fluorescence Intensity) of two to four independent experiments with two to three technical replicates each. Error bars show standard deviation. Individual data points are shown, with different symbols for each independent experiment. Data was normalized against the mean of the highest value (50 µg/ml D13-14) in each independent experiment to allow for comparison across multiple experiments. Statistical analysis was done by comparing each group to the PBS control using an ordinary one-way ANOVA with Dunnett's multiple comparisons test with a single pooled variance. **(B-E)** Schematic overview of genomic integration of Pfs230$^{\Delta D13/14}$ (B) and Pfs230$^{\Delta Cterm}$ (D), and the corresponding diagnostic integration PCR (C, E). HDR = Homology Directed Repair, HR = homology region, 3xFLAG = triple FLAG tag, 3'UTR = bidirectional 3'untranslated region of PBANKA_142660, GAPDH = promoter of Pf3D7_1462800. **(F)** Western blot analysis of Pfs230 (18F25) and Pfs48/45 (32F3) expression in wildtype, Pfs230$^{\Delta D13/14}$ and Pfs230$^{\Delta Cterm}$ late-stage gametocytes, isolated with Percoll (intact RBC and PVM membrane) or saponin (permeabilized RBC and PVM membrane). **(G)** Schematic illustration of localization of the Pfs230:Pfs48/45 complex on the parasite plasma membrane (PPM), and the effect of saponin on the red blood cell membrane (RBCM) and parasite vacuole membrane (PVM). **(H)** Representative immunofluorescence microscopy images of paraformaldehyde/glutaraldehyde-fixed NF54 wildtype, Pfs230$^{\Delta D13/14}$ and Pfs230$^{\Delta Cterm}$ stage V gametocytes. Parasites were stained for Pfs230 (green, RUPA-55), Pfs48/45 (magenta, 45.1), and DNA (DAPI, blue). Scale bar is 5 µm. ESID = Electronically Switchable Illumination and Detection brightfield image. **(I)** Binding assay with wildtype (white bar) and Pfs230$^{\Delta Cterm}$ (gray bar) female gametes, testing the binding of PDGM1400 (α-HIV-1 envelope glycoprotein), TB31F (α-Pfs48/45), and LMIV230-01 (α-Pfs230) antibodies at 1 µg/ml. Bars show the mean relative MFI of two independent experiments with two technical replicates each (±2000 live gametes per condition), error bars show standard deviation. MFI was normalized against the average of LMIV230-01 in the independent experiments. MFI values were compared within each parasite line to the PGDM1400 control using an ordinary two-way ANOVA and Šídák's multiple comparison test with a single pooled variance. ns = not significant; ***=p<0.001, ****=p<0.0001. **(J)** Representative histogram of female gamete binding assay as shown in Figure S6I, comparing NF54 wildtype (top) and Pfs230$^{\Delta Cterm}$ (bottom) gametes. **(K)** Exemplary plots to provide an overview for the gating strategy of macrogamete flow cytometry experiments. Live gametes (gate A) were gated based on side-scatter (SS) and forward scatter (FS). Single cells (gate B) were selected based on FS area (FS-A) versus height (FS-H), after which live cells (gate C) were selected based on absence of LD efluor780 staining. The geometric mean fluorescence intensity of the Dylight/AlexaFluor-488 channel of the resulting population was then used as the mean fluorescence intensity. Note that both gate A and D (containing dead gametes) were used in the case of the complement deposition assays, when no live/dead stain was used. *Related to Figure 3.*

bioRxiv preprint doi: https://doi.org/10.1101/2025.02.14.638310; this version posted June 15, 2025. The copyright holder for this preprint (which was not certified by peer review) is the author/funder, who has granted bioRxiv a license to display the preprint in perpetuity. It is made available under aCC-BY 4.0 International license.

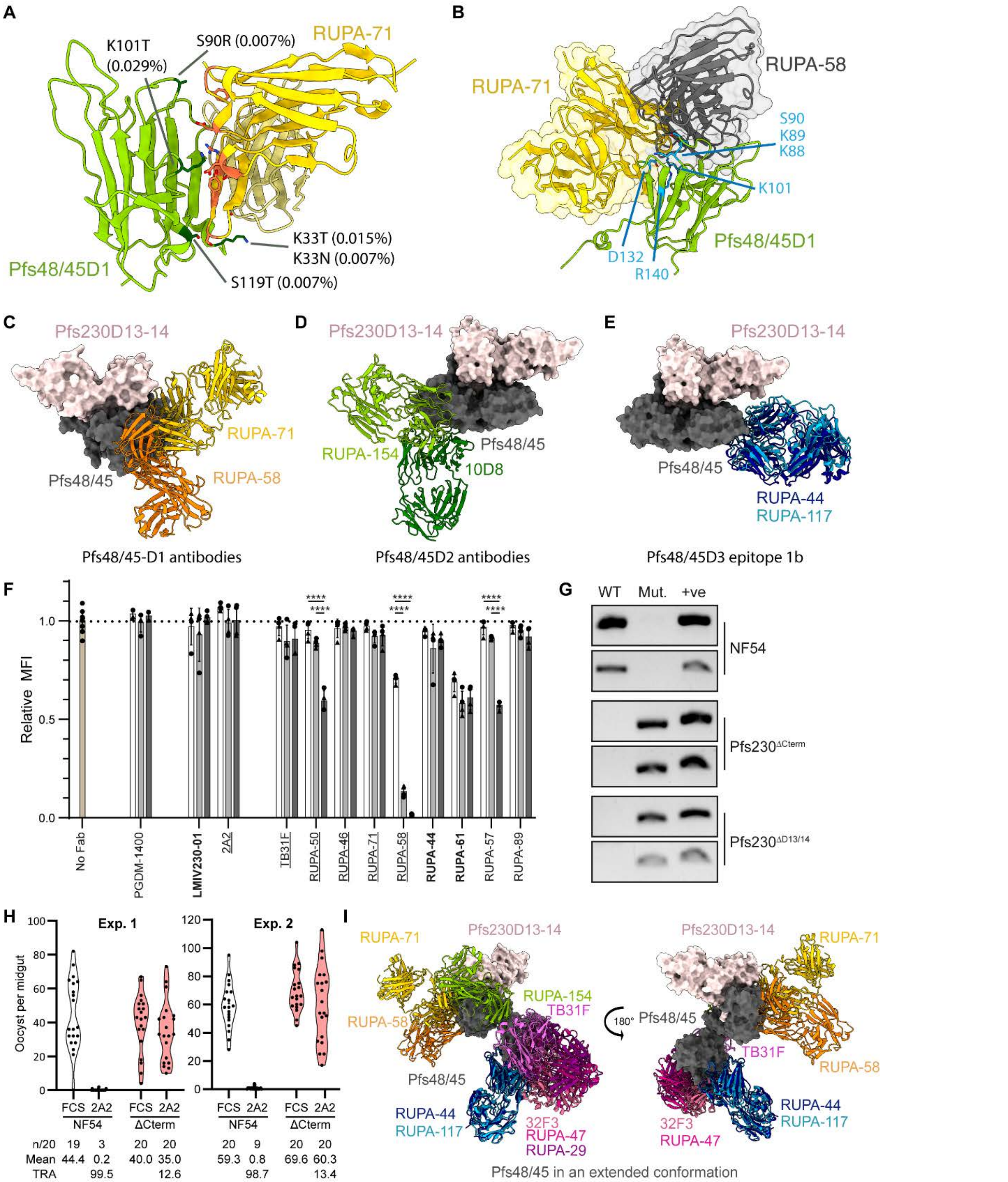

Figure S8: RUPA-71 epitope comparison and Pfs48/45-targeted Fab-induced dissociation of the Pfs230:Pfs48/45 complex *in vivo*. **(A)** RUPA-71 (yellow) bound to Pfs48/45 (yellow green) with Pfs48/45 residues with single nucleotide polymorphism in dark green and RUPA-71 residues that contact them in coral. **(B)** Overlay of Pfs48/45D1 (yellow green, cartoon) bound to antibodies RUPA-71 (gold) and RUPA-58 (dim gray; PDB ID: 8U1P). Pfs48/45 residues that contact both antibodies are indicated in blue. **(C-E)** Model of Pfs48/45 (dark gray, depicted in surface) bound to Pfs230D13-14 (misty rose, depicted in surface) in the disc-like conformation bound to (C) Pfs48/45D1 binders RUPA-71 (gold), RUPA-58 (orange, PDB ID: 8U1P)); (D) Pfs48/45D2 binders 10D8 (dark green, PDB ID: 7ZXF),

bioRxiv preprint doi: https://doi.org/10.1101/2025.02.14.638310; this version posted June 15, 2025. The copyright holder for this preprint (which was not certified by peer review) is the author/funder, who has granted bioRxiv a license to display the preprint in perpetuity. It is made available under aCC-BY 4.0 International license.

RUPA-154 (yellow green, PDB ID: 8U1P)); (E) Pfs48/45D3-1B binders (RUPA-44 (dark blue), RUPA-117 (sky blue, PDB ID: 7UNB)). **(F)** Live female *P. falciparum* NF54 macrogametes were incubated with increasing concentrations of Fab fragments (white bar: 1 µg/ml; light grey: 10 µg/ml; dark grey: 100 µg/ml), after which Pfs230 surface retention was measured by determining the binding of 18F25-DyLight488 by flow cytometry. MFI was normalized against the no Fab control to allow for averaging across experiments (two biological replicates with two technical replicates each). Bars depict mean ± standard deviation, different symbols depict different biological replicates. Anti-Pfs48/45 Fabs are sorted on potency as determined by the IC_{80} value (Underlined: IC_{80} < 10 µg/ml ; Bold: IC_{80} = 10-100 µg/ml, Others: IC_{80} > 100 µg/ml, see **Table S**1). Statistical analysis to test for a dose-dependent reduction in 18F25-488 binding by comparing all concentrations per individual Fab using an ordinary two-way ANOVA with a Šídák's multiple comparisons test with a single pooled variance as one family. Only significant comparisons are shown. **** = p<0.0001. **(G)** PCR analysis of genomic DNA isolated from midgut oocysts from standard membrane feeding assay experiments of Pfs230 wildtype and truncation parasite lines. Semi-nested PCRs were used to detect: WT = wildtype Pfs230 genomic DNA; Mut = Pfs230 truncation genomic DNA; +ve = positive control for *P. falciparum* genomic DNA, amplifying the Pfs25 gene (PF3D7_1031000). **(H)** Raw standard membrane feeding assay data of NF54 wildtype (white) and $Pfs230^{\Delta Cterm}$ (pink) with and without the addition of 10 µg/ml 2A2 and active human complement. TRA was calculated as percentage of reduction in mean oocyst per midgut compared to the FCS control. n/20 represents the number of mosquitoes that had at least 1 oocyst, "mean" is the average number of oocyst per mosquito midgut. **(I)** Model of Pfs48/45 (dark gray, depicted in surface) bound to Pfs230D13-14 (misty rose, depicted in surface) in the extended conformation overlayed with RUPA-71 (gold), RUPA-58 (orange, PDB ID: 8U1P), , RUPA-154 (green, PDB ID: 8U1P)), RUPA-44 (dark blue), RUPA-117 (sky blue, PDB ID: 7UNB), TB31F (orchid, PDB ID: 6E63), RUPA-29 (magenta, PDB ID: 7UXL), RUPA-47 (violet red, PDB ID: 7UNB), and 32F3 (pale violet red, PDB ID: 7ZWI)). *Related to Figure 4.*

bioRxiv preprint doi: https://doi.org/10.1101/2025.02.14.638310; this version posted June 15, 2025. The copyright holder for this preprint (which was not certified by peer review) is the author/funder, who has granted bioRxiv a license to display the preprint in perpetuity. It is made available under aCC-BY 4.0 International license.

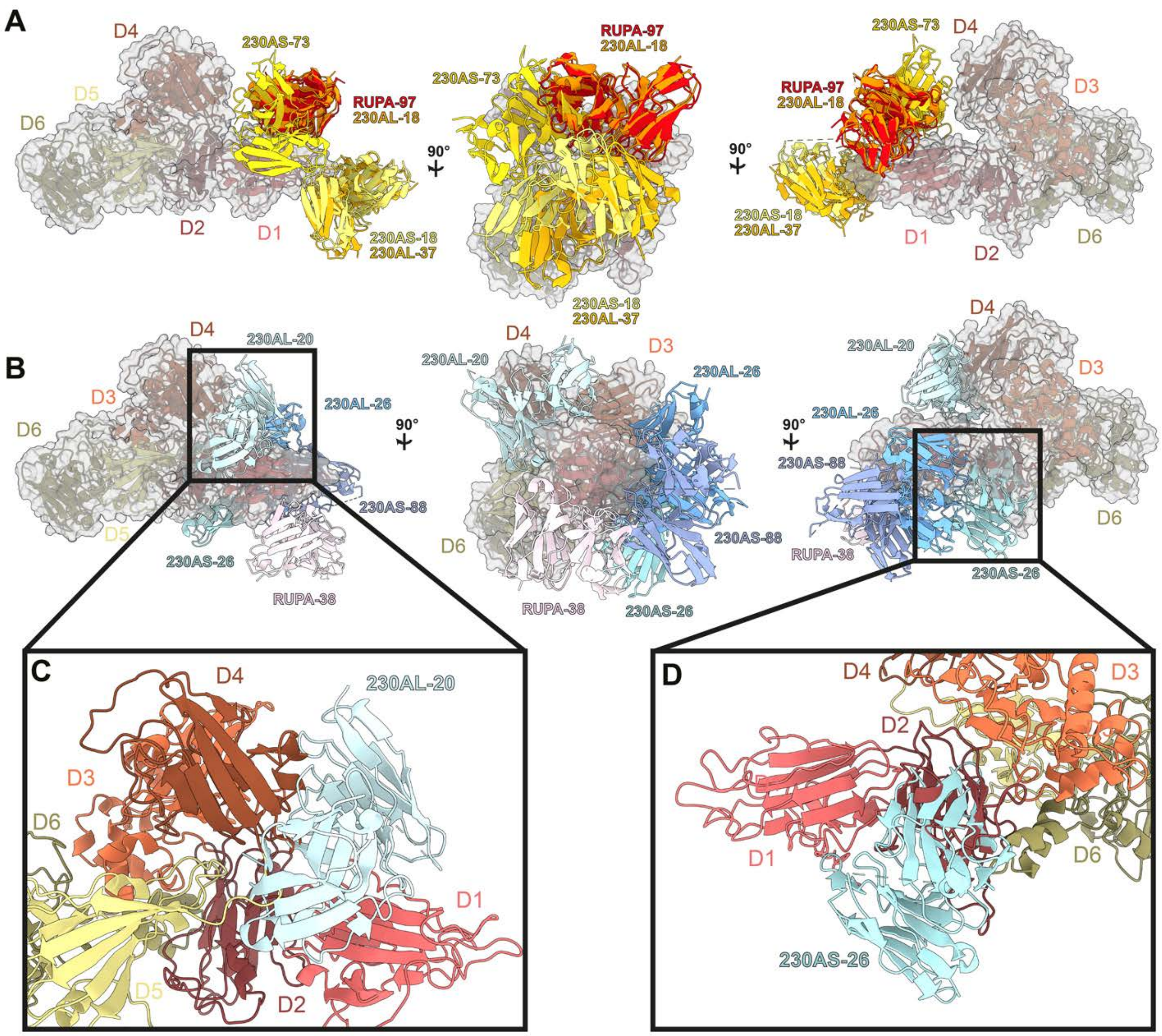

Figure S9. Overlay of Pfs230D1-EPA-elicted human mAbs (*17*). **(A)** Potent mAbs (TRA > 80% at 100 μg/mL in SMFA; shades of yellow and orange) are shown overlaid on Pfs230D1-6 (surface: gray, ribbons: coloured as done in Figure 1) compared to RUPA-97 (red). (**B-D**) Non-potent mAbs (TRA < 80% at 100 μg/mL in SMFA; shades of blue) are shown overlaid on Pfs230D1-6 compared to RUPA-38 (light pink) (B). Zoom in of (C) 230AL-20 and (D) 230AS-26 showcasing steric clashes that occur with Pfs230D4 and Pfs230D2, respectively – as examples of mAbs elicited via a single Pfs230 domain subunit vaccine exposing non-native epitopes that would be incompatible with full-length Pfs230 binding. *Related to Figure 6.*

bioRxiv preprint doi: https://doi.org/10.1101/2025.02.14.638310; this version posted June 15, 2025. The copyright holder for this preprint (which was not certified by peer review) is the author/funder, who has granted bioRxiv a license to display the preprint in perpetuity. It is made available under aCC-BY 4.0 International license.

SUPPLEMENTARY TABLES

Table S1. Overview of antibodies used or described in these studies. Shown are the targeted epitopes, structures in the RSCB/PDB database (structures in bold: this work), and estimates of the IC_{80} value (antibody concentration required to achieve 80% TRA in Standard Membrane Feeding Assay). [a] Estimate value from limited number of observations. [b] No blocking in High-Throughput membrane feeding assay at 100 µg/ml (32). *Related to Figures 1, 4, and 6.*

Target	Antibody	Epitope	IC_{80} (µg/ml)	Reference	Structure
Pfs230	RUPA-97	D1 – i	0.7	Ivanochko[20]	7UVQ/**9N5H**
	4F12	D1 – i	55	MacDonald[34], Ivanochko[20]	6OHG
	LMIV230-01	D1 – ii	48	Coelho[19], Ivanochko[20]	7UFW/7JUM/**9N5H**
	RUPA-38	D1 – ii	>>100	Ivanochko[20]	7UVI
	LMIV230-02	D1 – iii	>>100	Coelho[19], Ivanochko[20]	7UVS
	15C5	D1 – iii	>>100	Lee[75], Ivanochko[20]	7UVQ
	2A2	D4	1.9	Roeffen[49] , De Jong[38]	**9N5H/9N89**
	18F25	D7	10 to 30 [a]	Roeffen[49], Inklaar[37]	**9N5O/9N6U**
Pfs48/45	RUPA-58	D1-i	7.5	Kucharska[15]	8U1P
	RUPA-94	D1-i	<100 [a]	Fabra-García[32]	-
	RUPA-46	D1-ii	2 to 10 [a]	Fabra-García[32]	-
	RUPA-71	D1-ii	2 to 10 [a]	Fabra-García[32]	**9N5I/9N7K**
	RUPA-154	D2	103.1	Kucharska[15]	8U1P
	RUPA-160	D2	± 10 [a]	Fabra-García[32]	-
	10D8	D2	>350 [a]	Ko[14]	7ZXF/7ZXG/7ZWM
	TB31F	D3 - 1a	0.86	Kundu[41], Kucharska[15], Ko[14]	6E63/7ZXF/6H5N
	RUPA-29	D3 - 1a	0.4 to 2 [a]	Fabra-García[32]	7UXL
	RUPA-50	D3 - 1a	0.4 to 2 [a]	Fabra-García[32]	-
	RUPA-47	D3 - 1a	10 to 100 [a]	Fabra-García[32]	7UNB
	RUPA-57	D3 - 1a	>100 [b]	Fabra-García[32]	-
	RUPA-72	D3 - 1a	>100 [b]	Fabra-García[32]	-
	RUPA-89	D3 - 1a	>100 [b]	Fabra-García[32]	-
	32F3	D3 - 1ab	<25 [a]	Vermeulen[25], Ko[14]	7ZWI/7ZWF/7ZWM
	RUPA-61	D3 - 1b	± 10 [a]	Fabra-García[32]	-
	RUPA-44	D3 - 1b	11	Fabra-García[32]; Kucharska[15]	7UXL/**9N5I**
	RUPA-117	D3 - 1b	± 10 [a]	Fabra-García[32]	7UNB
Negative controls	PGDM1400	HIV Env.	n/a	n/a	n/a
	CIS43	PfCSP	n/a	n/a	n/a

bioRxiv preprint doi: https://doi.org/10.1101/2025.02.14.638310; this version posted June 15, 2025. The copyright holder for this preprint (which was not certified by peer review) is the author/funder, who has granted bioRxiv a license to display the preprint in perpetuity. It is made available under aCC-BY 4.0 International license.

Table S2: Pfs230:Pfs48/45 residue contact table. *Related to Figure 3*

Pfs48/45 Domain	Pfs48/45 Residue	BSA (Å^2)	Pfs230 Residue contacts
Domain 1	N38	18	3108
	E40	25	3011, 3013
	I41	60	3011, 3112, 3013
	S42	88	2986, 2997, 2998, 3011,3012
	G43	34	2997
	F44	15	2997
	I45	86	2984, 3108-3110
	G46	16	3110
	Y47	65	3110-3114
	K48	49	2982, 2997, 3114
	N50	17	2993
	E54	48	2990, 2993, 3001
	G55	9	2993
	V56	90	2942, 2991, 2993, 2994
	H57	78	2942, 3116, 3118
	E65	34	2943, 3118
	R67	30	3118
	S68	20	3117-3118
	I69	25	3116-3118
	F70	56	3115-3117
	C71	25	3114-3116
	T72	50	3113-3115
	I73	16	3114
	H74	24	3115
	S75	8	3112
	D80	17	3110
	N136	39	3001
	Y137	18	2999
Domain 2	N247	13	3111
	K248	71	3109-3111
	I249	11	3108
	I250	17	3108
Domain 3	I348	28	2839
	I349	11	2839
	F354	48	2871, 2980
	Q355	25	2870
	I369	53	2868, 2870, 2977
	V370	9	2977
	Y371	11	2870, 2871, 2978-2980
	D373	47	2980, 3113
	N377	31	3115
	G379	25	3111, 3112
	D380	26	3111, 3112
	E385	12	3079

bioRxiv preprint doi: https://doi.org/10.1101/2025.02.14.638310; this version posted June 15, 2025. The copyright holder for this preprint (which was not certified by peer review) is the author/funder, who has granted bioRxiv a license to display the preprint in perpetuity. It is made available under aCC-BY 4.0 International license.

Table S3. Data collection and refinement statistics for the RUPA-71, 2A2, and 18F25 Fab crystal structures. *Related to Figures 4 and 5.*

	**RUPA-71 Fab (PDB: 9N7K)**	**2A2 Fab (PDB: 9N89)**	**18F25 Fab (PDB: 9N6U)**
**Data collection**			
Space group	$P\ 2_1\ 2_1\ 2_1$	$P\ 2_1\ 2_1\ 2_1$	$P\ 2_1\ 2_1\ 2_1$
Cell dimensions			
a, *b*, *c* (Å)	64.66, 74.55, 204.90	59.97, 76.68, 91.87	37.64, 81.80, 140.53
a, b, g (°)	90, 90, 90	90, 90, 90	90, 90, 90
Resolution (Å)[a]	48.85 - 2.28 (2.36 –2.28)	28.51 - 1.20 (1.21 -1.20)	34.19 - 1.60 (1.63 - 1.60)
R_{sym} or R_{merge}[b]	38.1 (214.6)	6.9 (58.3)	7.2 (62.2)
R_{pim}[c]	9.9 (56.2)	4.2 (35.6)	4.6 (40.2)
I / s*I*	7.5 (1.7)	8.9 (2.1)	9.8 (1.8)
Completeness (%)	100 (100)	99.5 (98.2)	99.4 (98.6)
Redundancy	15.4 (15.2)	3.5 (3.6)	3.3 (3.3)
**Refinement**			
Resolution (Å)	2.28	1.20	1.60
No. reflections	711,113 (67,539)	891,870 (29,484)	365,949 (17,109)
No. unique reflections	46,105 (4434)	254,175 (8,275)	109,994 (5,231)
R_{work}[d] / R_{free}[e]	19.1/ 24.1	18.6 / 19.4	18.8 / 21.3
No. atoms	7037	3708	4056
Protein	6776	3343	3432
Ligand/ion	52	0	20
Water	209	427	604
B-factors			
Protein	41	12	17
Ligand/ion	53[f]	0	36[g]
Water	39	20	26
R.m.s. deviations			
Bond lengths (Å)	0.009	0.004	0.006
Bond angles (°)	0.99	0.78	0.83

[a]Values in brackets refer to the highest resolution bin.
[b]$R_{merge} = \Sigma_{hkl} \Sigma_i \mid I_{hkl,i} - <I_{hkl}> \mid / \Sigma_{hkl} <I_{hkl}>$
[c]$R_{pim} = \Sigma_{hkl} [1/(N-1)]^{1/2} \Sigma i \mid I_{hkl,i} - <I_{hkl}> \mid / \Sigma_{hkl} <I_{hkl}>$
[d]$R_{work} = (\Sigma \mid |Fo| - |Fc| \mid) / (\Sigma \mid |F_o|)$ - for all data except R_{free} (see footnote e).
[e]5% of data were used for the R_{free} calculation.
[f]Four glycerol and seven 1,2-ethanediol molecules
[g]Four SO_4 ions

bioRxiv preprint doi: https://doi.org/10.1101/2025.02.14.638310; this version posted June 15, 2025. The copyright holder for this preprint (which was not certified by peer review) is the author/funder, who has granted bioRxiv a license to display the preprint in perpetuity. It is made available under aCC-BY 4.0 International license.

Table S4: 2A2, 18F25, and RUPA-71 contact table in Kabat numbering. *Related to Figures 4 and 5.*

Pfs230 Residue	Structural Element	BSA (Å²)	2A2 HC Residue	Structural Element	BSA (Å²)	2A2 KC Residue	Structural Element	BSA (Å²)
H1159	D4: β2-β3 loop	37	N33	HCDR1	16	S1	N-term / FR1-IMGT	27
E1160	D4: β3	14	N35	FR2-IMGT	2	I2	N-term / FR1-IMGT	8
Y1194	D4: β5	27	W47	FR2-IMGT	22	Q27	KCDR1	73
Q1196	D4: β5	68	N50	FR2-IMGT	19	S28	KCDR1	8
E1198	D4: β5-β6 loop	20	D52	HCDR2	21	N30	KCDR1	6
Y1205	D4: β6	34	H55	HCDR2	58	D91	KCDR3	8
K1206	D4: β6-β7 loop	51	G57	HCDR2	23	Y92	KCDR3	80
G1207	D4: β6-β7 loop	21	T58	HCDR2	11	S93	KCDR3	42
L1208	D4: β6-β7 loop	170	T59	FR3-IMGT	60	S94	KCDR3	74
N1209	D4: β6-β7 loop	107	Y60	FR3-IMGT	8	P95	KCDR3	12
S1210	D4: β7	54	N61	FR3-IMGT	1	L96	KCDR3	26
V1211	D4: β7	44	Q62	FR3-IMGT	51			
T1215	D4: α1	31	K65	FR3-IMGT	14			
Q1250	D4: β10	33	T100	HCDR3	1			
T1252	D4: β10	9	L101	HCDR3	20			
Q1255	D4: β10-β11 loop	4	Y102	HCDR3	70			
V1256	D4: β11	8	G103	HCDR3	17			
V1257	D4: β11	70	S105	HCDR3	32			
K1259	D4: β11	41						
K1261	D4: β11	15						

Pfs230 Residue	Structural Element	BSA (Å²)	18F25 HC Residue	Structural Element	BSA (Å²)	18F25 KC Residue	Structural Element	BSA (Å²)
K1751	D7: β4-β5 loop (D7-ID)	36	Y32	HCDR1	33	T30	KCDR1	16
V1752	D7: β4-β5 loop (D7-ID)	47	W33	HCDR1	10	N31	KCDR1	10
I1754	D7: β4-β5 loop (D7-ID)	27	Y52	HCDR2	23	D32	KCDR1	32
E1756	D7: β4-β5 loop (D7-ID)	35	D54	HCDR2	9	Y49	FR2-IMGT	24
V1801	D7: β4-β5 loop (D7-ID)	20	D56	HCDR2	12	S50	KCDR2	4
L1802	D7: β4-β5 loop (D7-ID)	79	R58	HCDR2	11	Y53	KCDR2	32
D1803	D7: β4-β5 loop (D7-ID)	52	R94	HCDR3	2	Y55	FR3-IMGT	7
N1804	D7: β4-β5 loop (D7-ID)	7	L96	HCDR3	27	H91	KCDR3	28
T1806	D7: β4-β5 loop (D7-ID)	32	Y97	HCDR3	42	Y92	KCDR3	85
F1807	D7: β4-β5 loop (D7-ID)	115	L98	HCDR3	70	S93	KCDR3	1
E1808	D7: β4-β5 loop (D7-ID)	40						
K1809	D7: β4-β5 loop (D7-ID)	2						
K1856	D7: β8-β9 loop	25						
D1857	D7: β8-β9 loop	1						

Pfs48/45 Residue	Structural Element	BSA (Å²)	RUPA-71 HC Res.	Structural Element	BSA (Å²)	RUPA-71 KC Res.	Structural Element	BSA (Å²)
K33	D1: N-term	28	E2	N-term / FR1-IMGT	47	K32	KCDR1	40
S35	D1: N-term	14	G26	HCDR1	19	L46	FR2-IMGT	20
S36	D1: N-term	11	F27	HCDR1	14	Y49	FR2-IMGT	57
K88	D1: β3-β4 loop	35	T28	HCDR1	26	T56	FR3-IMGT	79

bioRxiv preprint doi: https://doi.org/10.1101/2025.02.14.638310; this version posted June 15, 2025. The copyright holder for this preprint (which was not certified by peer review) is the author/funder, who has granted bioRxiv a license to display the preprint in perpetuity. It is made available under aCC-BY 4.0 International license.

K89	D1: β3-β4 loop	40	D31	HCDR1	24	S67	FR3-IMGT	39
S90	D1: β3-β4 loop	45	Y32	HCDR1	25			
K101	D1: β4-β5 loop	81	D52C	HCDR2	48			
Q104	D1: β4-β5 loop	61	E53	HCDR2	18			
S119	D1: α1	21	R94	HCDR3	14			
Y126	D1: α1-β7 loop	47	G96	HCDR3	24			
E127	D1: β7	42	L97	HCDR3	60			
I128	D1: β7	42	R98	HCDR3	86			
E129	D1: β7	61	W99	HCDR3	59			
E130	D1: β7	76	Y100	HCDR3	96			
N131	D1: β7	51	D100A	HCDR3	35			
D132	D1: β7-β8 loop	71	S100B	HCDR3	69			
T133	D1: β7-β8 loop	113	D101	HCDR3	30			
N134	D1: β7-β8 loop	24						
P135	D1: β7-β8 loop	26						
R140	D1: β8	6						

bioRxiv preprint doi: https://doi.org/10.1101/2025.02.14.638310; this version posted June 15, 2025. The copyright holder for this preprint (which was not certified by peer review) is the author/funder, who has granted bioRxiv a license to display the preprint in perpetuity. It is made available under aCC-BY 4.0 International license.

Table S5: Oligonucleotides used in this study. Red or green nucleotides depict restriction sites used for cloning. Underlined nucleotides were used for overlap-PCR. Blue nucleotides highlight the CRISPR/Cas9 guide RNA sequence. Orange nucleotides are three sequential guanosine nucleotides used for template switching, as described in (*85*). *Related to Methods.*

	Function	Sequence
		**HDR template - primers**
p1	5'HR(-A) Pfs230-tag FW	cagttCGTCTCtggagCCACCATATGTACATAAAGATATACATTTCTCATTAG
p2	5'HR-A Pfs230-tag RV	aGCcCTactcTGAGGTTCTGGGATTATATAATTAGGATCAAATGTG
p3	5'HR Pfs230-tag RV	gtcaaCGTCTCataccAAGCTTTCTCAAGTATTTTG
p4	3'HR Pfs230-tag FW	cagttCGTCTCtgcacTCGTAAATAATTAATCAAACATATATATAATCAAAAGG
p5	3'HR Pfs230-tag RV	cagttCGTCTCtatggGGACTCTAAGATATTTCTTTCGCTAATCC
p6	H2B_mSc_biUTR-A FW	cagttGGTCTCTggagCGTCTCttaagCTAAATGGATGATTCCCCTCTTTGCAATATG
p7	H2B_mSc_biUTR-A RV	CAGTTggtctctTGACatttcatatttgcatgtgcatattaaaagtatcg
p8	H2B_mSc_biUTR-B FW	cagttggtctctGTCAgtctctgcataattttcgctatttaaatatat
p9	H2B_mSc_biUTR-B RV	cagttGGTCTCTatggCGTCTCtGTGCgTTCGAATCAACTCCGTTCAGTTAAAAATATTAC
p10	5'HR Pfs230ΔD13-14 FW	CAGTcgtctcTagagGGGATGATGTACATTTATTTTATCCTCCTC
p11	5'HR Pfs230ΔD13-14 RV	CAGTcgtctcTatccaccTGTTTTTTTTTCATCTATTTTTATTTTATCCATTGTAC
p12	5'HR Pfs230ΔCterm FW	CAGTcgtctcTagagCAATTGGTAAAGATATATGTAAATATGATGTTACTAC
p13	5'HR Pfs230ΔCterm RV	CAGTcgtctcTatccaccATTAGGATCAAATGTGATTCGTATAGTATAATTTG
p4	3'HR Pfs230Δ FW	See p4
p14	3'HR Pfs230Δ RV	cagtCGTCTCtcggtGGACTCTAAGATATTTCTTTCGCTAATCC
p15	GAPDH_swap FW	cagtGCTAGCGAAAAGAATTAAAAAGCCGAAGAAAAAATATATG
p16	GAPDH_swap RV	cagtGAGCTCGAGCTATGAAAAACATGGGTGTG
p17	C-terminal 3xFLAG FW	cagtGAATTcgtctcTggatCTGGTGATTACAAAGATCATGATGG
p18	C-terminal 3xFLAG RV	cagtAAGCTTcgtctcTcttaGTCATGATCCTTGTAATCTATATCGTG
		**HDR template - oligonucleotides**
p19	5'HR-B anneal Pfs230 FW	CTAATTATATAATCCCAGAACCTCAgagtAGgGCtATCATTAAATATGTAGATCTGCA AGATAAAAATTTTGCAAAATACTTGAGAAAGCTTggtaTGAGACGttgac
p20	5'HR-B anneal Pfs230 RV	gtcaaCGTCTCataccAAGCTTTCTCAAGTATTTTGCAAAATTTTTATCTTGCAGATCTA CATATTTAATGATaGCcCTactcTGAGGTTCTGGGATTATATAATTAG
p21	FLAG-C-tag FW	gatccgtctctGGTACATCTGGTGATTACAAAGATCATGATGGAGATTATAAAGATCAC GATATAGATTACAAGGATCATGACGGTTCAGGTGAACCAGAAGCATAAgtGAGACG
p22	FLAG-C-tag RV	aattCGTCTCacTTATGCTTCTGGTTCACCTGAACCGTCATGATCCTTGTAATCTATA TCGTGATCTTTATAATCTCCATCATGATCTTTGTAATCACCAGATGTACCagagacg
		**CRISPR/Cas9 guide plasmid oligonucleotides**
p23	Guide Pfs230 Cterm FW	tattgTTAATGATGGCTCTTGATTG
p24	Guide Pfs230 Cterm RV	aaacCAATCAAGAGCCATCATTAAc
p25	Guide Pfs230 D13 FW	tattgATTCTATTACATTATCAAGA
p26	Guide Pfs230 D13 RV	aaacTCTTGATAATGTAATAGAATc
		**Template-switch oligonucleotide for hybridoma sequencing**
p27	RT-PCR template switch FW	*AAGCAGTGGTATCAACGCAGAGTACATrGrGrG*
p28	RT-PCR for mIgK RV	TTGTCGTTCACTGCCATCAATC
p29	RT-PCR for mIgH RV	AGCTGGGAAGGTGTGCACAC
p30	Universal ISPCR primer FW	AAGCAGTGGTATCAACGCAGAG
p31	mIgK RV	ACATTGATGTCTTTGGGGTAGAAG
p32	mIgH RV	GGGATCCAGAGTTCCAGGTC

bioRxiv preprint doi: https://doi.org/10.1101/2025.02.14.638310; this version posted June 15, 2025. The copyright holder for this preprint (which was not certified by peer review) is the author/funder, who has granted bioRxiv a license to display the preprint in perpetuity. It is made available under aCC-BY 4.0 International license.

**Integration PCR to check genomic integration**		
p33	iGP2$^{230\text{-tag}}$ - WT FW	CTAATTATATAATCCCAGAACCTCAATCAAGAGCC
p34	iGP2$^{230\text{-tag}}$ - WT RV	GCAAAAATGAACAAACATCACACGAAGATGTG
p35	iGP2$^{230\text{-tag}}$ - 5’HR FW	GAACATATCTTACCAGGAGCCATTACAACAG
p36	iGP2$^{230\text{-tag}}$ - 5’HR RV	CTCCATCATGATCTTTGTAATCACCAGATG
p37	iGP2$^{230\text{-tag}}$ - 3’HR FW	GATGAACGATTTCTTAATATTGATAAGTATCATGTG
p34	iGP2$^{230\text{-tag}}$ - 3’HR RV	see p34
p38	230^{trunc} - WT FW	GAGCCATCATTAAATATGTAGATCTGCAAG
p34	230^{trunc} - WT RV	see p34
p39	230$^{\Delta\text{Cterm}}$ - 5’HR FW	CAGCTGATAAATATAATACATTTAGTAAAGAC
p40	230$^{\Delta\text{D13/14}}$ - 5’HR FW	CAAAGTTTTTATGGTGTTTCTCTTGATCATC
p41	230^{trunc} - 5’HR RV	CGTGATCTTTATAATCTCCATCATGATC
p42	230^{trunc} - 3’HR FW	CACACGATTTTAATTATCACACCCATG
p34	230^{trunc} - 3’HR RV	see p34
**Integration PCR midgut oocyst**		
p38	WT - reaction A - FW	see p38
p43	WT - reaction A - RV	CTTACGTATTCTCATTCTCATTGTGGAC
p38	WT - reaction B - FW	see p38
p34	WT - reaction B - RV	see p34
p42	Mut - reaction A - FW	see p42
p43	Mut - reaction A - RV	see p43
p42	Mut - reaction B - FW	see p42
p34	Mut - reaction B - RV	see p34
p44	Pfs25 - reaction A - FW	ATTTACATTATAAAAAAGCATACTGA
p45	Pfs25 - reaction A - RV	TTATTCTTTTAAAAATGAATAAACTTTAC
p46	Pfs25 - reaction B - FW	TGAAAATAGTATAAACATAATGCTTAG
p47	Pfs25 - reaction B - RV	ATGAATAAACTTTACAGTTTGTTTCT

bioRxiv preprint doi: https://doi.org/10.1101/2025.02.14.638310; this version posted June 15, 2025. The copyright holder for this preprint (which was not certified by peer review) is the author/funder, who has granted bioRxiv a license to display the preprint in perpetuity. It is made available under aCC-BY 4.0 International license.

Table S6: Amino acid sequence of variable chains of mAbs 18F25 and 2A2. *Related to Methods.*

mAb	Region	Amino acid sequence
18F25	Heavy chain (VDJ)	QVQLQQSGAELARPGASVKLSCKASGYTFTNYWMQWVKQRPGQGLEWFGAIYPGDGDTRYT<br>QKFKGKATLTADKSSSTAYMQLSSLASEDSAVYYCARSLYLGAMDYWGQGTSVTVSS
	Light chain (VJ)	DIVMTQSHKFMSTSVGDRVSITCKASQDVTNDVAWYQQKPGQSPKLLIYSASYRYTGVPDRFTG<br>SGSGTDFIFTISSVQAEDLAVYYCQQHYSAPPTFGGGTKLEIK
2A2	Heavy chain (VDJ)	EVQLQQSGPELEKPGASVKISCKASGYSITDYNMNWVKLSNGKSLEWIGNIDPSHGGTTYNQKF<br>KGKATLTVDKSSSTAYMQLKSLTSEDSAVYHCARSTLYGNSAMDCWGQGTSVTVSS
	Light chain (VJ)	SIVMTQTPKFLLVPAGDRVTITCKASQSVNNDVTWYQQKPGQSPKLLIYYASNRYTGVPDRFTGS<br>GYGTDFTFTISTVQAEDLAVYFCQQDYSSPLTFGAGTKLEL
