# Supplementary material for: Structure of endogenous Pfs230:Pfs48/45 in complex with potent malaria transmission-blocking antibodies
Source: bioRxiv. 2025 Feb 15:2025.02.14.638310. Preprint. [Version 1] doi: 10.1101/2025.02.14.638310 (PMC11844449; doi:10.1101/2025.02.14.638310)
Supplement: Supplementary file 1 [file NIHPP2025.02.14.638310v1-supplement-1.pdf]

bioRxiv preprint doi: https://doi.org/10.1101/2025.02.14.638310; this version posted February 15, 2025. The copyright holder for this preprint (which was not certified by peer review) is the author/funder, who has granted bioRxiv a license to display the preprint in perpetuity. It is made available under aCC-BY 4.0 International license.

Supplementary Figures

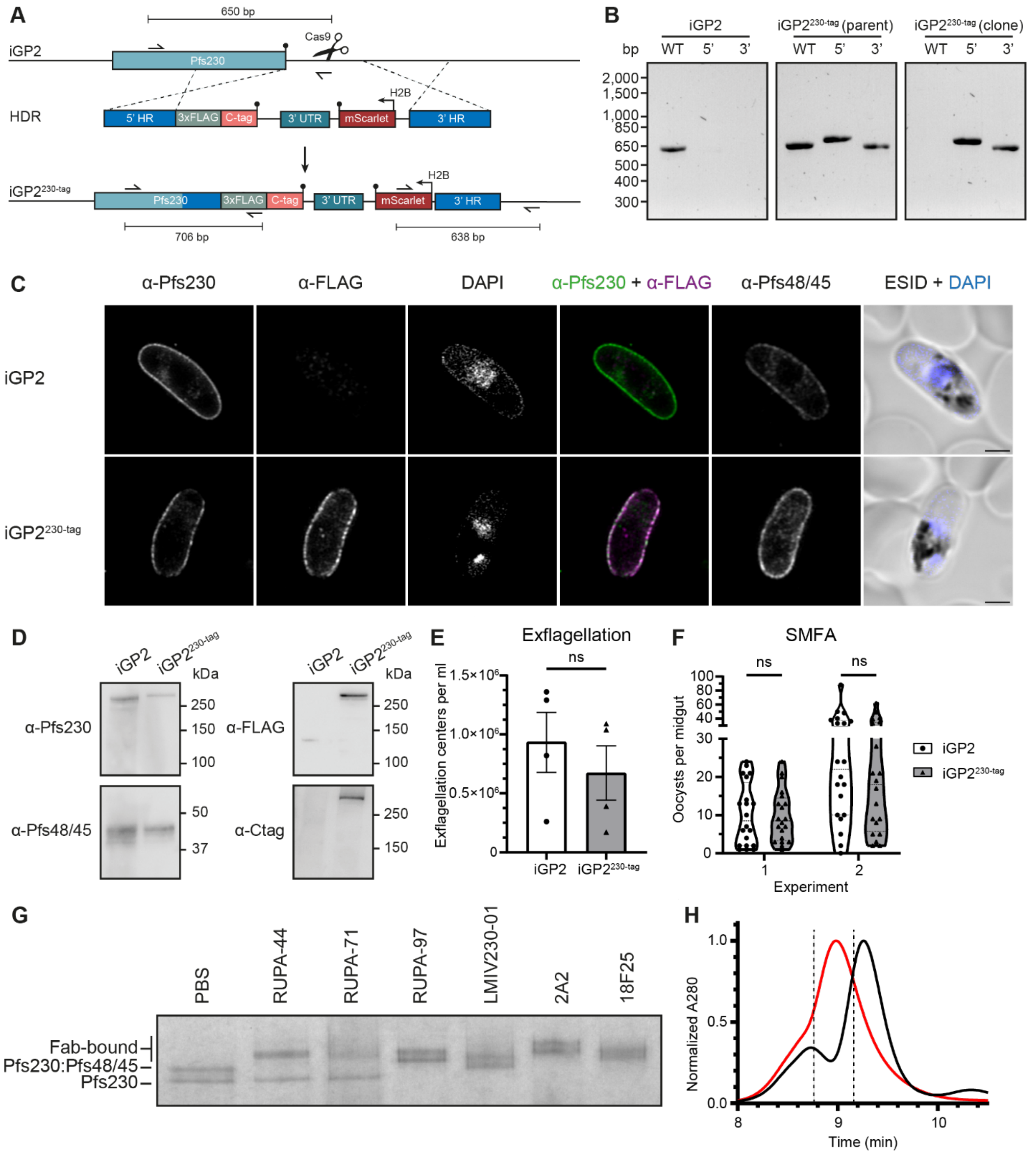

Figure S1: The iGP2$^{230\text{-tag}}$ parasite line allows for purification of endogenous Pfs230:Pfs48/45 complex recognized by transmission-blocking antibodies. (A) Schematic overview of the genomic modification to obtain the iGP2$^{230\text{-tag}}$ parasite line. HDR = Homology Directed Repair, HR = homology region, 3xFLAG = triple FLAG tag, C-tag = EPEA* tag, 3'UTR = bidirectional 3'untranslated region of PBANKA_142660, H2B = promoter of Pf3D7_1105100. (B) Genomic integration PCR to confirm 5' and 3' integration and the absence of wildtype parasites. Primers and expected PCR product are indicated in (A). (C) Immunofluorescence microscopy images of paraformaldehyde/glutaraldehyde-fixed and permeabilized iGP2 wildtype and iGP2$^{230\text{-tag}}$ stage V gametocytes.

bioRxiv preprint doi: https://doi.org/10.1101/2025.02.14.638310; this version posted February 15, 2025. The copyright holder for this preprint (which was not certified by peer review) is the author/funder, who has granted bioRxiv a license to display the preprint in perpetuity. It is made available under aCC-BY 4.0 International license.

Parasites were stained for Pfs230 (green, RUPA-55), FLAG-tag (magenta, M2), and Pfs48/45 (45.1), and DAPI (blue). Scalebar represents 5 µm. ESID = Electronically Switchable Illumination and Detection brightfield image. (D) Western blot analysis of iGP2 and iGP2$^{230\text{-tag}}$ to confirm FLAG-tag and C-tag integration. (E) Exflagellation of stage V iGP2 (white) and iGP2$^{230\text{-tag}}$ (gray) gametocytes. Bars represent mean ± standard deviation of number of exflagellation centers per ml from four independent cultures (dots). No statistical significance was found using an unpaired t-test. (F) Transmission of iGP2 (white) and iGP2$^{230\text{-tag}}$ (gray) to mosquitoes in two independent standard membrane feeding experiments. Dots represent the number of oocysts per midgut *Anopheles stephensi* mosquitoes (n=20 per experiment) in two independent standard membrane feeding assays using iGP2 (white) and iGP2$^{230\text{-tag}}$ (gray) parasites. Statistical analysis was done using a Mann-Whitney test with Holm-Šídák's multiple comparisons test ($\alpha = 0.05$). ns = not significant. (G) Coomassie blue stained Blue Native PAGE gel of purified Pfs230:Pfs48/45 (and free Pfs230) incubated with molar excess of the following individual Fab fragments: RUPA-44 (Pfs48/45D3-1a), RUPA-71 (Pfs48/45D1-ii), RUP-97 (Pfs230D1-i), LMIV230-01 (Pfs230D1-ii), 2A2 (Pfs230D4), or 18F25 (Pfs230D7). (H) Normalized absorption at 280 nm during high-pressure liquid size-exclusion chromatography of Pfs230:Pfs48/45 (black) or Pfs230-Pfs48/45:6Fab (red) complexes. Dotted lines indicate fractions of eluted Pfs230:Pfs48/45:6Fab that were used for cryo-electron microscopy. Related to **Figure 1**.

bioRxiv preprint doi: https://doi.org/10.1101/2025.02.14.638310; this version posted February 15, 2025. The copyright holder for this preprint (which was not certified by peer review) is the author/funder, who has granted bioRxiv a license to display the preprint in perpetuity. It is made available under aCC-BY 4.0 International license.

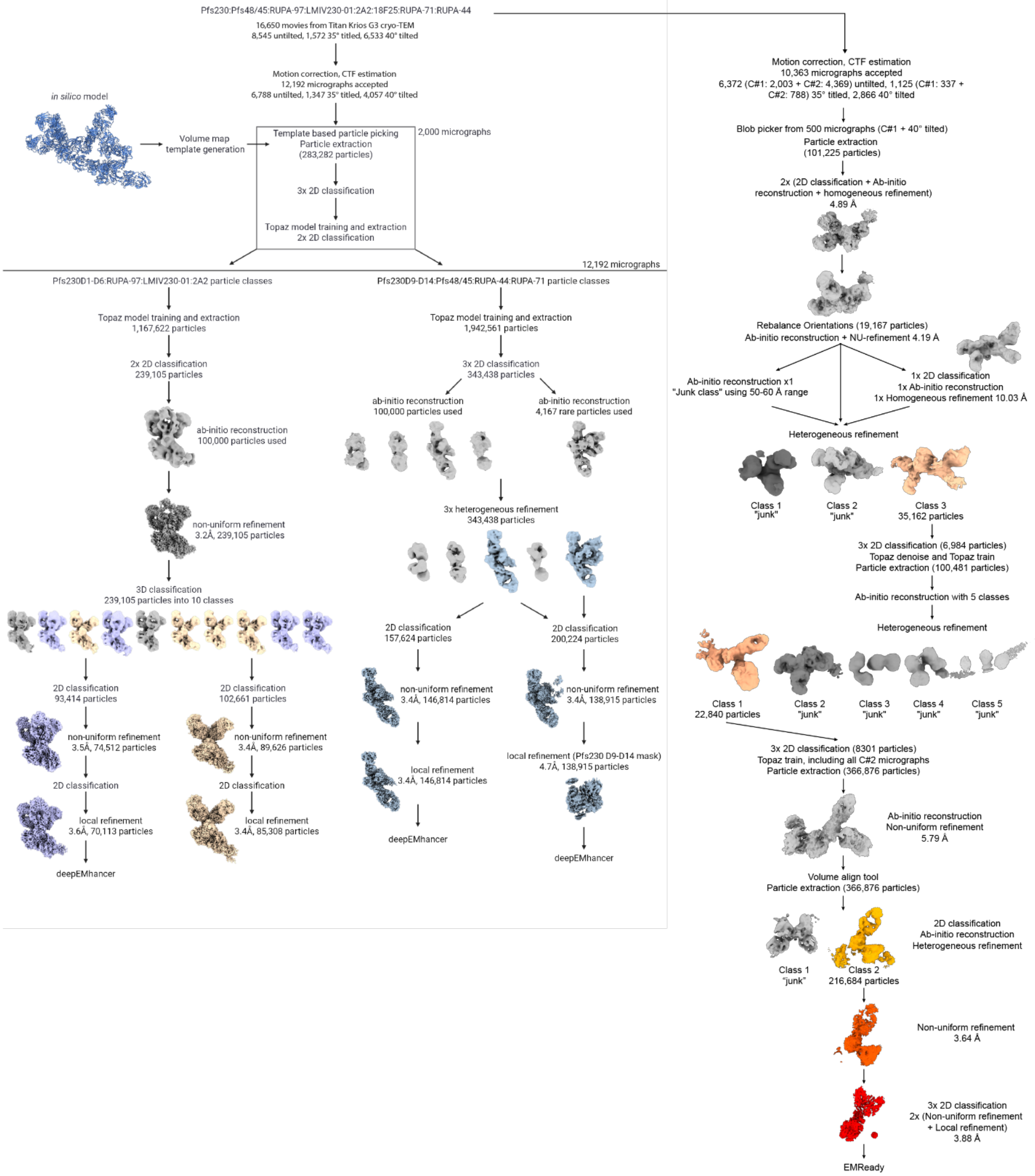

Figure S2: Cryo-EM processing workflow. Shown from left to right are the maps of Pfs230D1-6:LMIV230-01:RUPA-97:2A2, Pfs230D1-6:LMIV230-01:RUPA-97, Pfs230D13-14:Pfs48/45:RUPA-44:RUPA-71, the focused refinement for Pfs230D9-14:Pfs48/45, and the Pfs230D7-8:18F25 map. Abbreviations for the two data collection sessions are abbreviated in the Pfs230D7-8:18F25 workflow (C#1: collection C#2: collection 2). Related to **Figure 1**.

bioRxiv preprint doi: https://doi.org/10.1101/2025.02.14.638310; this version posted February 15, 2025. The copyright holder for this preprint (which was not certified by peer review) is the author/funder, who has granted bioRxiv a license to display the preprint in perpetuity. It is made available under aCC-BY 4.0 International license.

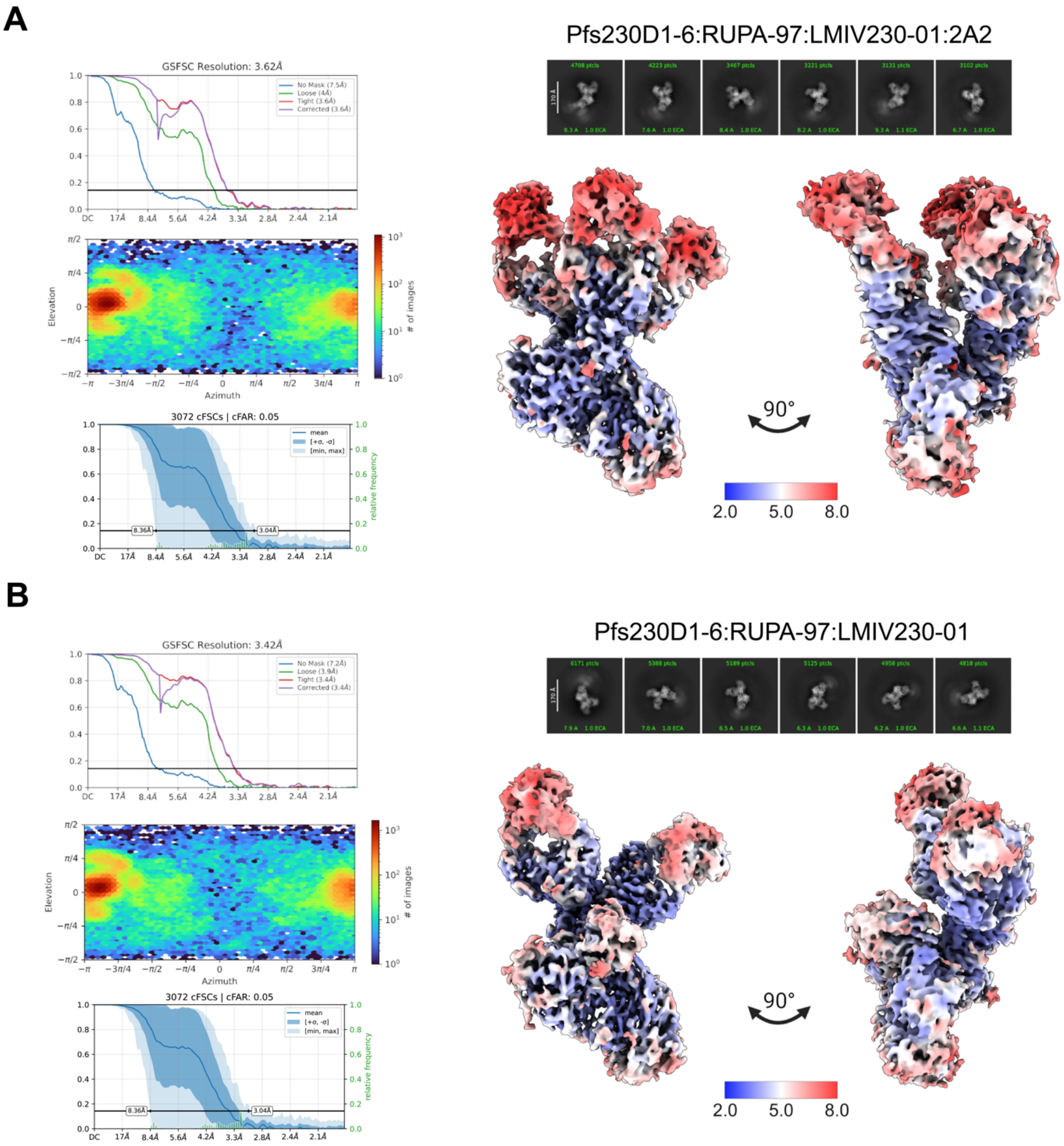

bioRxiv preprint doi: https://doi.org/10.1101/2025.02.14.638310; this version posted February 15, 2025. The copyright holder for this preprint (which was not certified by peer review) is the author/funder, who has granted bioRxiv a license to display the preprint in perpetuity. It is made available under aCC-BY 4.0 International license.

C

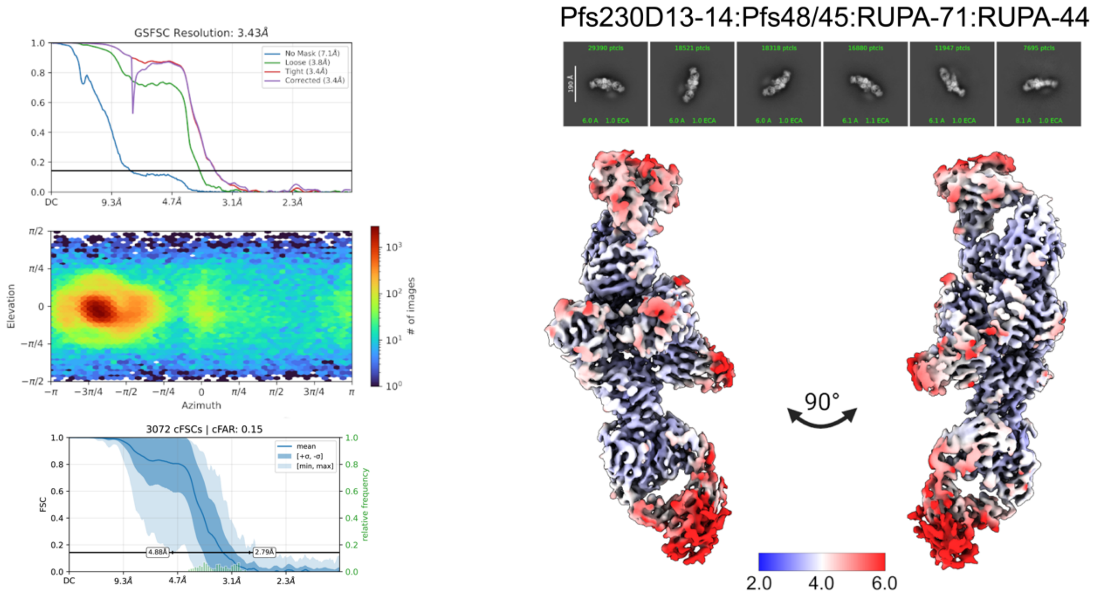

D

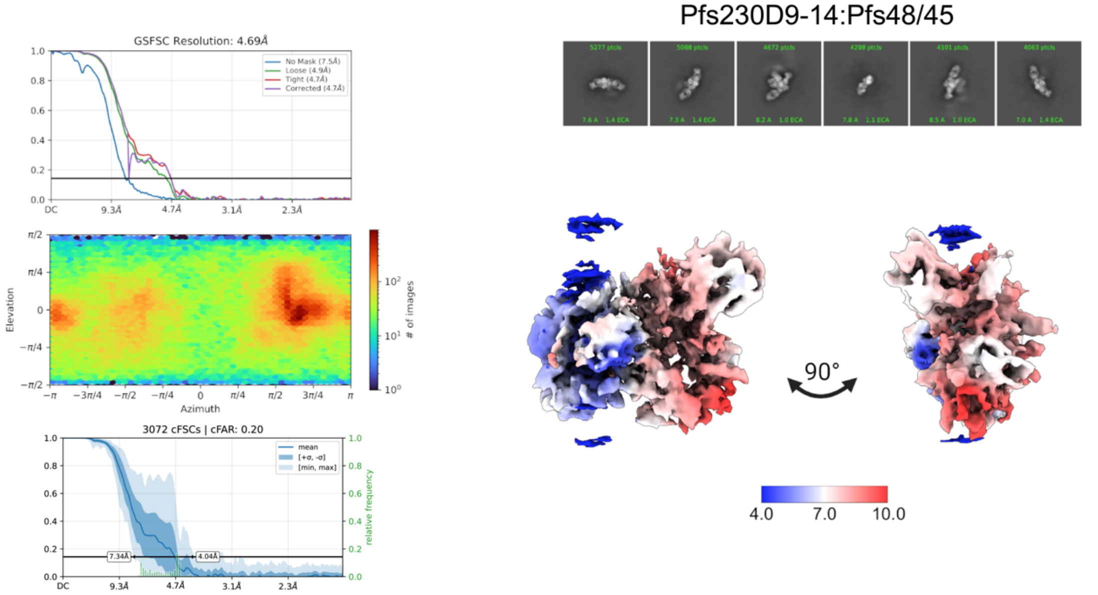

bioRxiv preprint doi: https://doi.org/10.1101/2025.02.14.638310; this version posted February 15, 2025. The copyright holder for this preprint (which was not certified by peer review) is the author/funder, who has granted bioRxiv a license to display the preprint in perpetuity. It is made available under aCC-BY 4.0 International license.

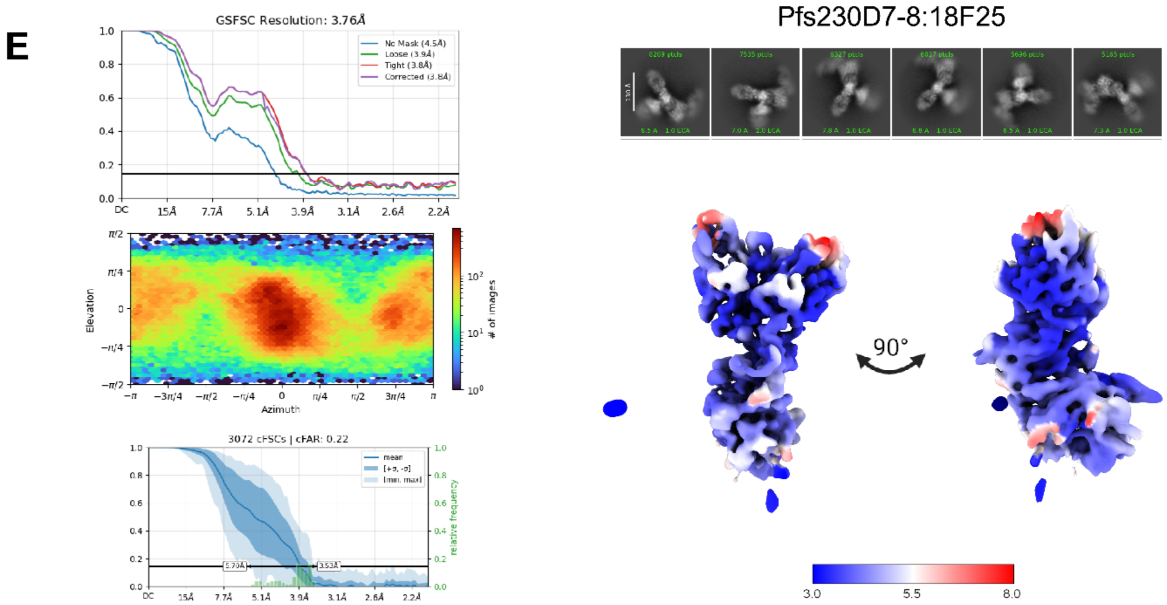

Figure S3: Cryo-EM map validation data. Shown are the structures of (A) Pfs230D1-6:RUPA-97:LMIV230-01:2A2; (B) Pfs230D1-6:RUPA-97:LMIV230-01; (C) Pfs230D13-14:Pfs48/45:RUPA-44:RUPA-71; (D) focused refinement for Pfs230D9-14:Pfs48/45; and (E) Pfs230D7-8:18F25. The Fourier shell correlation curves following a gold-standard refinement with correction for the effects of masking for varying masks, the viewing direction particle distribution, and cFAR plot and score are shown (left panel). Additionally, representative 2D classes and cryo-EM maps coloured by local resolution are shown for each processed map (right panel). Related to **Figure 1**.

bioRxiv preprint doi: https://doi.org/10.1101/2025.02.14.638310; this version posted February 15, 2025. The copyright holder for this preprint (which was not certified by peer review) is the author/funder, who has granted bioRxiv a license to display the preprint in perpetuity. It is made available under aCC-BY 4.0 International license.

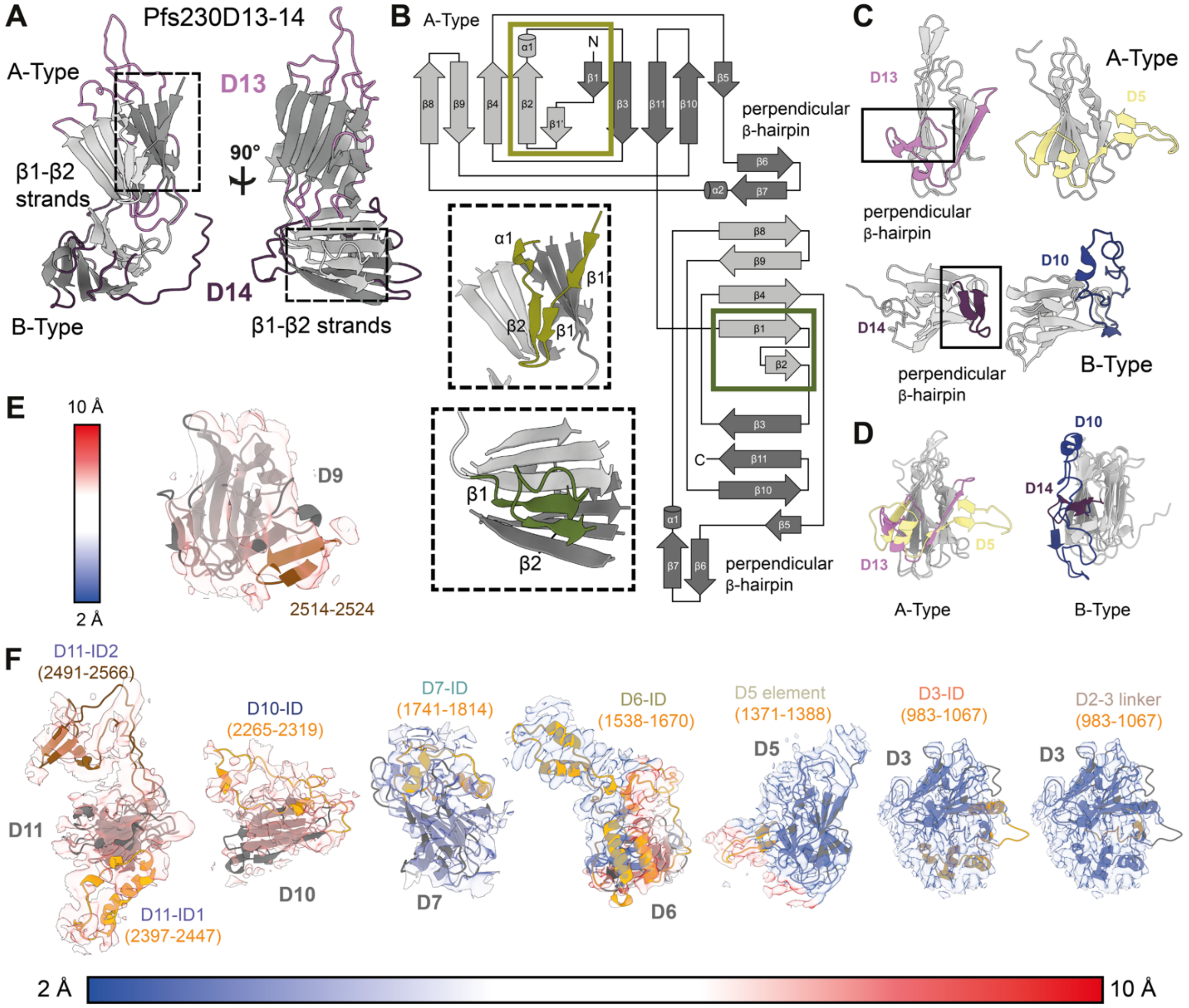

Figure S4: Structural features of Pfs230 6-Cys domains. (A) Pfs230D13-14 is shown as prototypical tandem A- and B-type 6-Cys domains of Pfs230. Loops of D13 and D14 are coloured according to the colouring scheme in Figure 1. The first β-sheet of the A-type and B-type domains is coloured distinctly (light gray) from the second β-sheet (gray). The distinguishing features of the A- and B-type domains (β1 to β2 strands) are indicated through the dashed box. (B) Secondary structure topology of the two 6-Cys domains are shown with distinguishing features of A- (olive) and B-type (green) domains (β1 to β2 strands) indicated by box (inset) with a 3D representation of these differences shown. (C-D) Comparison of prototypical tandem 6-Cys domains (D13 and D14) to D5 and D10 respectively, (C) side by side and (D) overlaid, highlighting the structural differences of D5 and D10 at the perpendicular β-hairpin region. The regions that are structurally distinct are coloured according to the scheme in Figure 1. (E) Pfs230D9 (gray) and a portion of the Pfs230D11-ID2 (2514-2524, brown) overlaid on top of the map which is coloured according to local resolution (left scale). (F) Pfs230 domains that harbour IDs (Pfs230D11, D10, D7, D6, D5, and D3, gray) or structural elements contributing to ID clustering (Pfs230D3 and D5) are shown (Pfs230D11-ID2: brown, rest: orange) as ribbon representations with the map overlaid (shown at 5 Å radius around the models) and coloured according to local resolution (scale bar below). Related to **Figure 2.**

bioRxiv preprint doi: https://doi.org/10.1101/2025.02.14.638310; this version posted February 15, 2025. The copyright holder for this preprint (which was not certified by peer review) is the author/funder, who has granted bioRxiv a license to display the preprint in perpetuity. It is made available under aCC-BY 4.0 International license.

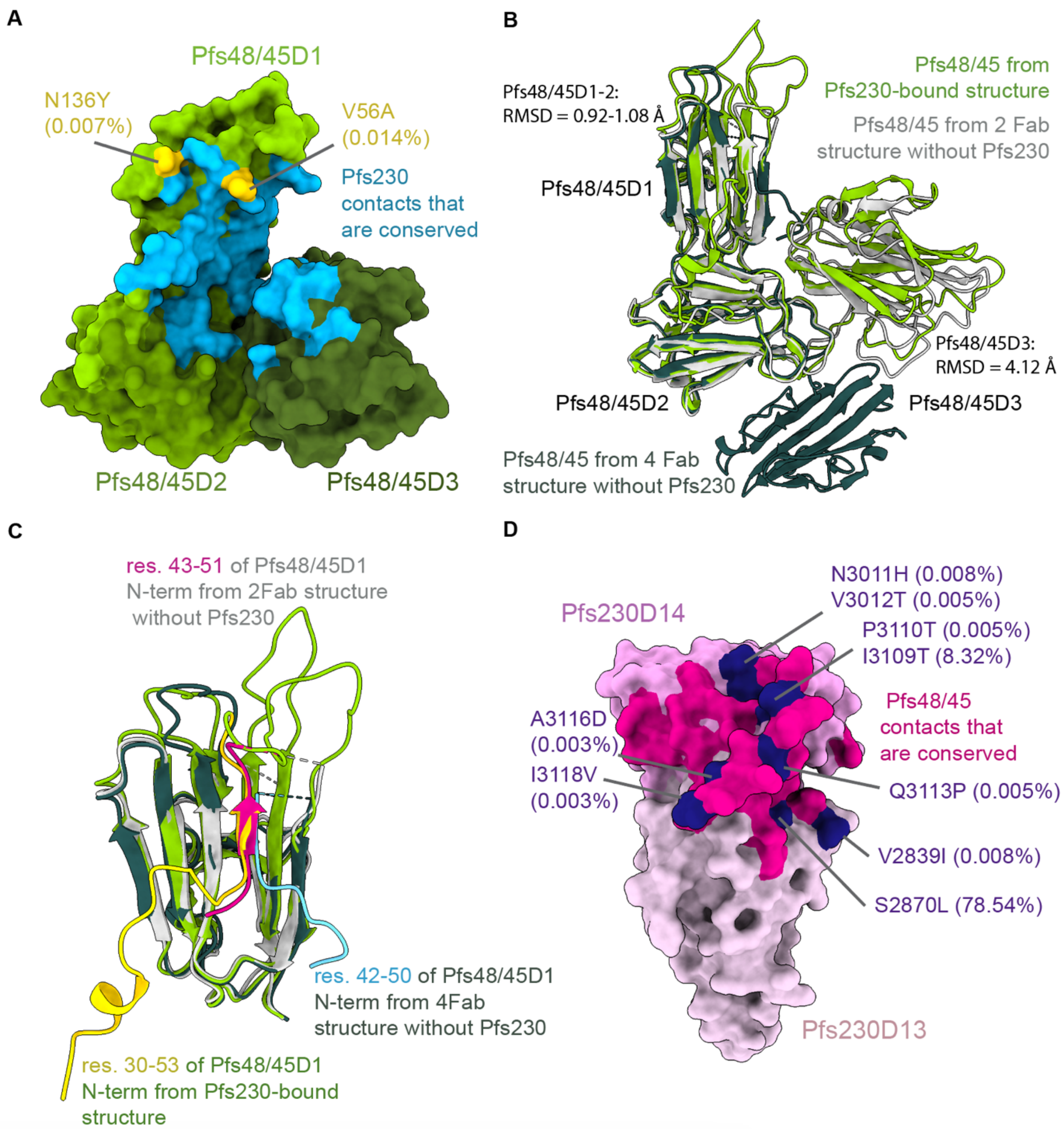

Figure S5: Comparison of Pfs48/45 conformation across determined structures and sequence conservation of Pfs230:Pfs48/45 binding site. (A) Structure of Pfs48/45 depicted as surface (yellow green, olive drab, and dark olive drab for Pfs48/45D1-3, respectively) with the Pfs230 binding site shown in sky blue and single nucleotide polymorphisms present within the epitope shown in gold. (B) Overlay of Pfs48/45 structures from Pfs48/45-Pfs230-6Fab complex (yellow green), Pfs48/45-4Fab complex (dark slate grey, PDB ID: 8U1P), and Pfs48/45-2 Fab complex (light grey, PDB ID: 7ZXF). (C) Overlay of N-terminal region of Pfs48/45-Pfs230-6Fab complex (green, yellow N-term), Pfs48/45-4Fab complex (dark slate grey, light blue N-term, PDB ID: 8U1P), and Pfs48/45-2 Fab complex (light grey, dark violet N-term, PDB ID: 7ZXF). (D) Pfs230D13 (thistle) and D14 (plum) shown as surface with the Pfs48/45 binding site in dark pink and SNPs in indigo. Related to **Figure 3.**

bioRxiv preprint doi: https://doi.org/10.1101/2025.02.14.638310; this version posted February 15, 2025. The copyright holder for this preprint (which was not certified by peer review) is the author/funder, who has granted bioRxiv a license to display the preprint in perpetuity. It is made available under aCC-BY 4.0 International license.

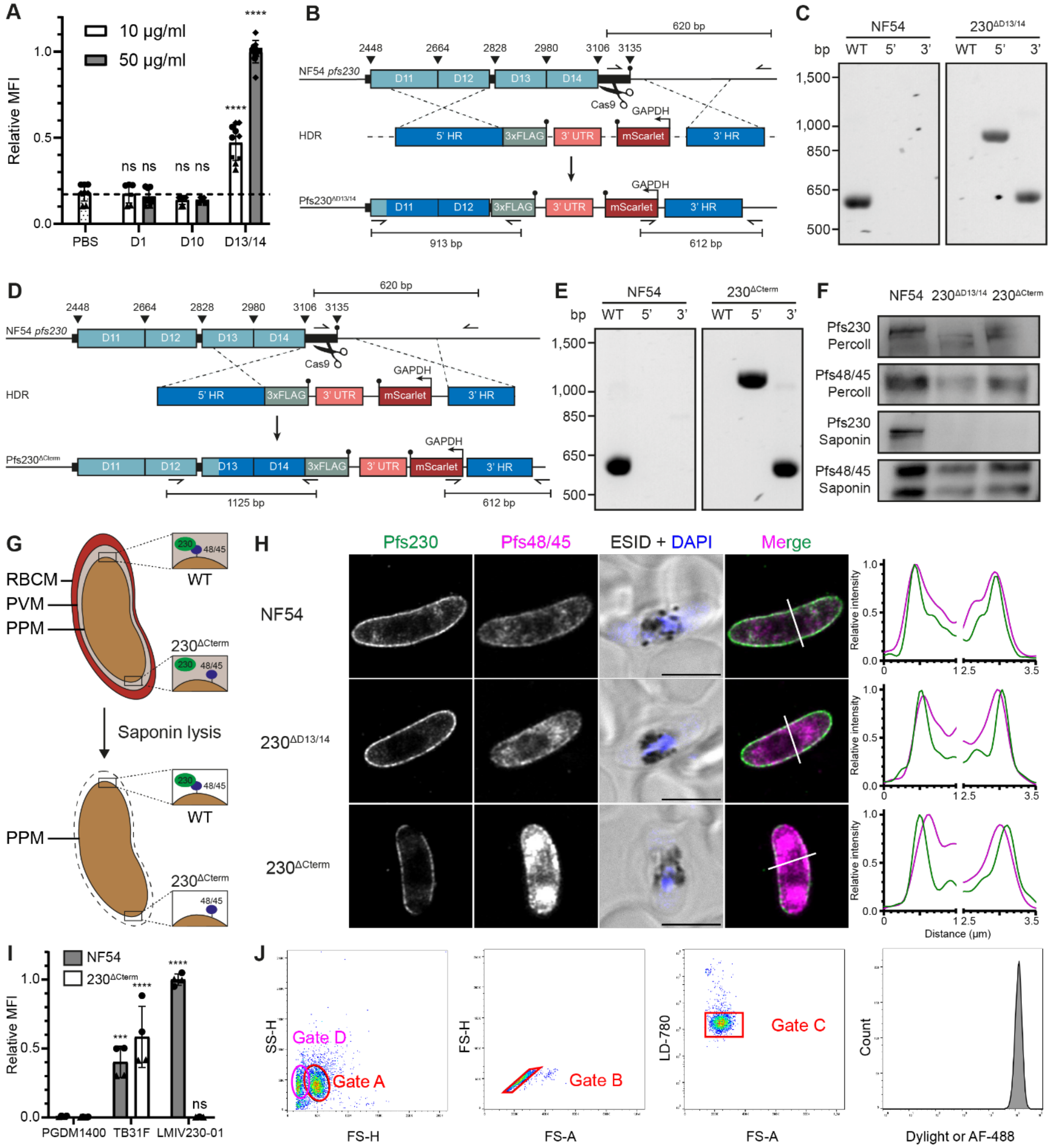

Figure S6: Pfs230:Pfs48/45 interactions are mediated by the C-terminal region Pfs230D13-14-Cterm *in vivo*. (A) Female gamete binding assay with recombinantly purified Pfs230D1, Pfs230D10 and Pfs230D13/14-Cterm at 10 µg/ml (white bar) or 50 µg/ml (gray bar). Bars show mean MFI (Mean Fluorescence Intensity) of two to four independent experiments with two to three technical replicates each. Error bars show standard deviation. Individual data points are shown, with different symbols for each independent experiment. Data was normalized against the mean of the highest value (50 µg/ml D13-14) in each independent experiment to allow for comparison across multiple experiments. Statistical analysis was done by comparing each group to the PBS control using an ordinary one-way ANOVA with Dunnett's multiple comparisons test with a single pooled variance. (B-E) Schematic overview of genomic integration of Pfs230$^{\Delta D13/14}$ (B) and Pfs230$^{\Delta Cterm}$ (D), and the corresponding diagnostic integration PCR (C, E). HDR = Homology Directed Repair, HR = homology region, 3xFLAG = triple FLAG tag, 3'UTR = bidirectional

bioRxiv preprint doi: https://doi.org/10.1101/2025.02.14.638310; this version posted February 15, 2025. The copyright holder for this preprint (which was not certified by peer review) is the author/funder, who has granted bioRxiv a license to display the preprint in perpetuity. It is made available under aCC-BY 4.0 International license.

3'untranslated region of PBANKA_142660, GAPDH = promoter of Pf3D7_1462800. (F) Western blot analysis of Pfs230 (18F25) and Pfs48/45 (32F3) expression in wildtype, Pfs230$^{\Delta D13/14}$ and Pfs230$^{\Delta Cterm}$ late-stage gametocytes, isolated with Percoll (intact RBC and PVM membrane) or saponin (permeabilized RBC and PVM membrane). (G) Schematic illustration of localization of the Pfs230:Pfs48/45 complex on the parasite plasma membrane (PPM), and the effect of saponin on the red blood cell membrane (RBCM) and parasite vacuole membrane (PVM). (H) Representative immunofluorescence microscopy images of paraformaldehyde/glutaraldehyde-fixed NF54 wildtype, Pfs230$^{\Delta D13/14}$ and Pfs230$^{\Delta Cterm}$ stage V gametocytes. Parasites were stained for Pfs230 (green, RUPA-55), Pfs48/45 (magenta, 45.1), and DNA (DAPI, blue). Right inset shows intensity plot profile analysis along the indicated section, normalized against maximum value along the profile per signal. Scale bar is 5 µm. ESID = Electronically Switchable Illumination and Detection brightfield image. (I) Binding assay with wildtype (white bar) and Pfs230$^{\Delta Cterm}$ (gray bar) female gametes, testing the binding of PDGM1400 (α-HIV-1 envelope glycoprotein), TB31F (α-Pfs48/45), and LMIV230-01 (α-Pfs230) antibodies at 1 µg/ml. Bars show the mean relative MFI of two independent experiments with two technical replicates each, error bars show standard deviation. MFI was normalized against the average of LMIV230-01 in the independent experiments. MFI values were compared within each parasite line to the PGDM1400 control using an ordinary two-way ANOVA and Šídák's multiple comparison's test with a single pooled variance. ns = not significant ; ***=p<0.001, ****=p<0.0001. (J) Exemplary plots to provide an overview for the gating strategy of macrogamete flow cytometry experiments. Live gametes (gate A) were gated based on side-scatter (SS) and forward scatter (FS). Single cells (gate B) were selected based on FS area (FS-A) versus height (FS-H), after which live cells (gate C) were selected based on absence of LD efluor780 staining. The geometric mean fluorescence intensity of the Dylight/AlexaFluor-488 channel of the resulting population was then used as the mean fluorescence intensity. Note that both gate A and D (containing dead gametes) were used in the case of the complement deposition assays, when no live/dead stain was used. Related to **Figure 3** and **Methods**.

bioRxiv preprint doi: https://doi.org/10.1101/2025.02.14.638310; this version posted February 15, 2025. The copyright holder for this preprint (which was not certified by peer review) is the author/funder, who has granted bioRxiv a license to display the preprint in perpetuity. It is made available under aCC-BY 4.0 International license.

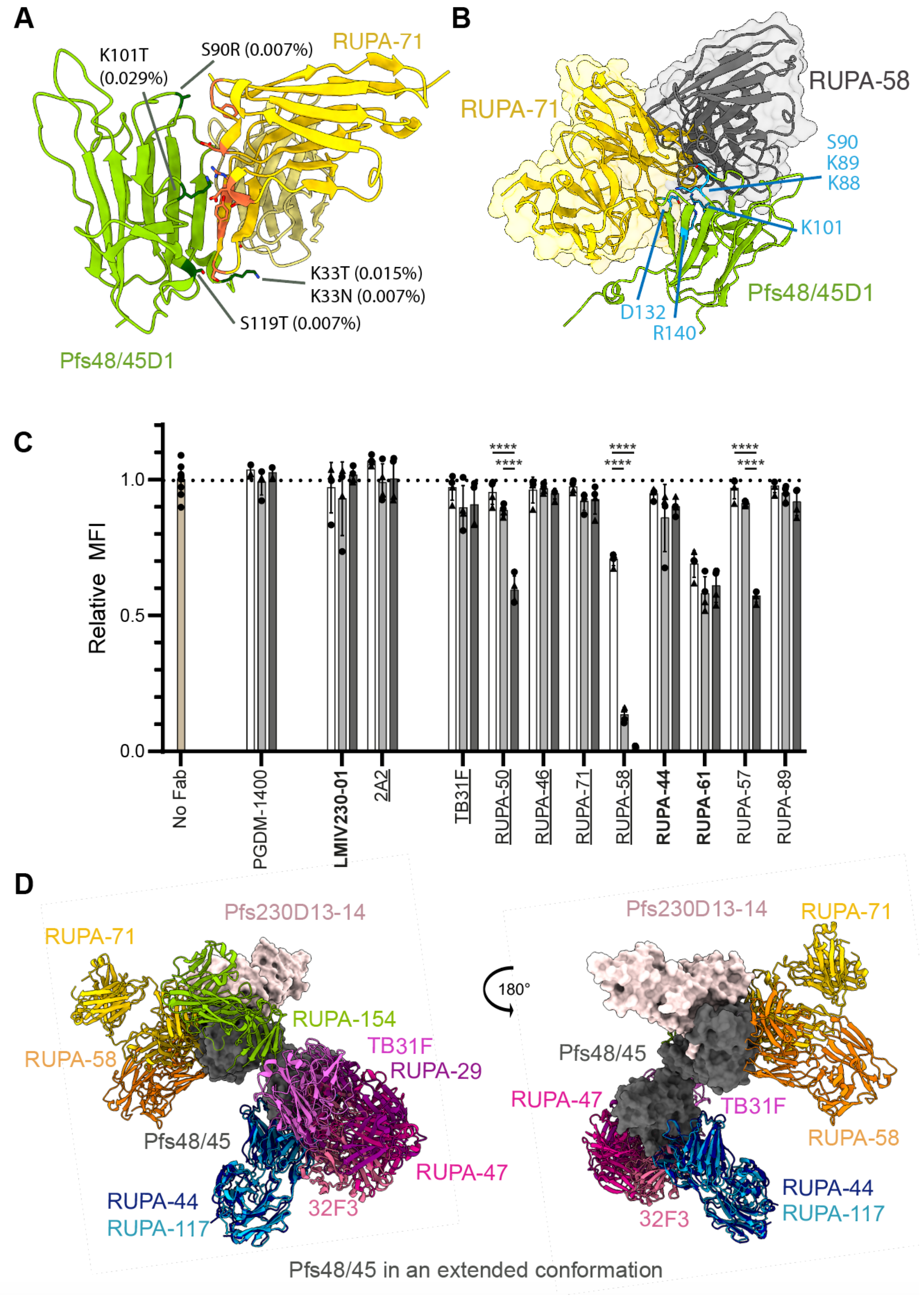

779
780

bioRxiv preprint doi: https://doi.org/10.1101/2025.02.14.638310; this version posted February 15, 2025. The copyright holder for this preprint (which was not certified by peer review) is the author/funder, who has granted bioRxiv a license to display the preprint in perpetuity. It is made available under aCC-BY 4.0 International license.

Figure S7: RUPA71 epitope comparison and Pfs48/45-targeted Fab-induced dissociation of the Pfs230:Pfs48/45 complex *in vivo*. (A) RUPA-71 (yellow) bound to Pfs48/45 (yellow green) with Pfs48/45 residues with single nucleotide polymorphism in dark green and RUPA-71 residues that contact them in coral. (B) Overlay of Pfs48/45D1 (yellow green, cartoon) bound to antibodies RUPA-71 (gold) and RUPA-58 (dim gray; PDB ID: 8U1P). Pfs48/45 residues that contact both antibodies are indicated in blue. (C) Live female *P. falciparum* NF54 macrogametes were incubated with increasing concentrations of Fab fragments (white bar: 1 µg/ml; light grey: 10 µg/ml; dark grey: 100 µg/ml), after which Pfs230 surface retention was measured by determining the binding of 18F25-DyLight488 by flow cytometry. MFI was normalized against the no Fab control to allow for averaging across experiments (two biological replicates with two technical replicates each). Bars depict mean ± standard deviation, different symbols depict different biological replicates. Anti-Pfs48/45 Fabs are sorted on potency as determined by the IC_{80} value (Underlined: $IC_{80} < 10$ µg/ml ; Bold: IC_{80} = 10-100 µg/ml, Others: $IC_{80} > 100$ µg/ml, see Table S1). Statistical analysis to test for a dose-dependent reduction in 18F25-488 binding by comparing all concentrations per individual Fab using an ordinary two-way ANOVA with a Šídák's multiple comparisons test with a single pooled variance as one family. Only significant comparisons are shown. **** = p<0.0001. (D) Model of Pfs48/45 (dark gray, depicted in surface) bound to Pfs230D13-14 (misty rose, depicted in surface) in the extended conformation overlayed with RUPA-71 (gold), RUPA-58 (orange, PDB ID: 8U1P), , RUPA-154 (green, PDB ID: 8U1P)), RUPA-44 (dark blue), RUPA-117 (sky blue, PDB ID: 7UNB), TB31F (orchid, PDB ID: 6E63), RUPA-29 (magenta, PDB ID: 7UXL), RUPA-47 (violet red, PDB ID: 7UNB), and 32F3 (pale violet red, PDB ID: 7ZWI)). Related to **Figure 4.**

bioRxiv preprint doi: https://doi.org/10.1101/2025.02.14.638310; this version posted February 15, 2025. The copyright holder for this preprint (which was not certified by peer review) is the author/funder, who has granted bioRxiv a license to display the preprint in perpetuity. It is made available under aCC-BY 4.0 International license.

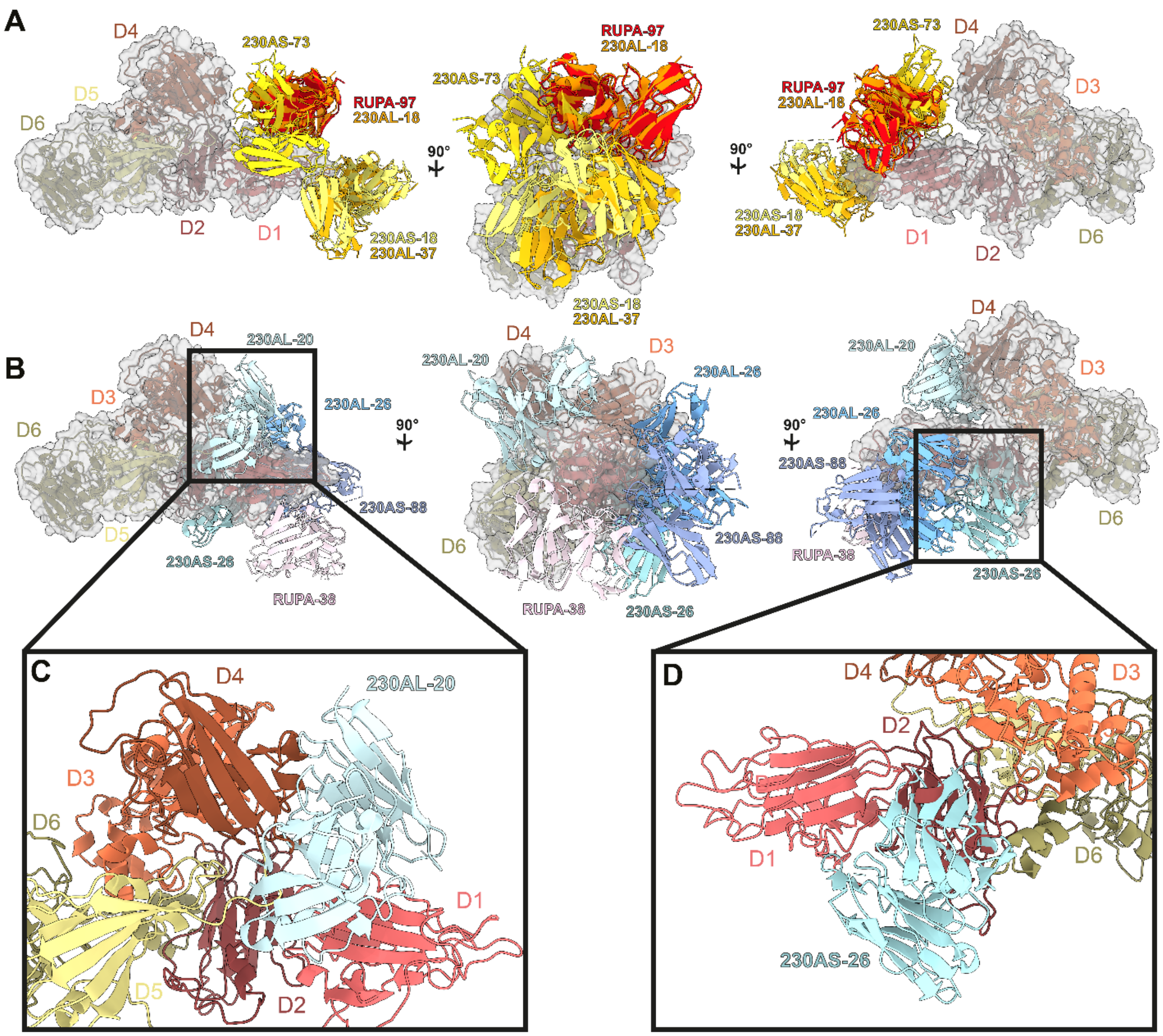

Figure S8. Overlay of Pfs230D1-EPA-elicted human mAbs (19). (A) Potent mAbs (TRA > 80% at 100 µg/mL in SMFA; shades of yellow and orange) are shown overlaid on Pfs230D1-6 (surface: gray, ribbons: coloured as done in Figure 1) compared to RUPA-97 (red). (A) Non-potent mAbs (TRA < 80% at 100 µg/mL in SMFA; shades of blue) are shown overlaid on Pfs230D1-6 compared to RUPA-38 (light pink). Zoom in of (C) 230AL-20 and (D) 230AS-26 showcasing steric clashes that occur with Pfs230D4 and Pfs230D2, respectively – as examples of mAbs elicited via a single Pfs230 domain subunit vaccine exposing non-native epitopes that would be incompatible with full-length Pfs230 binding. Related to **Figure 5**.

bioRxiv preprint doi: https://doi.org/10.1101/2025.02.14.638310; this version posted February 15, 2025. The copyright holder for this preprint (which was not certified by peer review) is the author/funder, who has granted bioRxiv a license to display the preprint in perpetuity. It is made available under aCC-BY 4.0 International license.

Supplementary Tables

Table S1. Overview of antibodies used or described in these studies. Shown are the targeted epitopes, structures in the RSCB/PDB database (structures in bold: this work), and estimates of the IC_{80} value (antibody concentration required to achieve 80% TRA in Standard Membrane Feeding Assay). * Estimate value from limited number of observations. ** No blocking in High-Throughput membrane feeding assay at 100 µg/ml (34). Related to **Figures 1, 4, and 5.**

Target	Antibody	Epitope	IC_{80} (µg/ml)	Reference	Structure
Pfs230	RUPA-97	D1 – i	0.7	Ivanochko[22]	7UVQ / **9N5H**
	4F12	D1 – i	55	MacDonald[36], Ivanochko[22]	6OHG
	LMIV230-01	D1 – ii	48	Coelho[21], Ivanochko[22]	7UFW / 7JUM / **9N5H**
	RUPA-38	D1 – ii	>>100	Ivanochko[22]	7UVI
	LMIV230-02	D1 – iii	>>100	Coelho[21], Ivanochko[22]	7UVS
	15C5	D1 – iii	>>100	Lee, Ivanochko[22]	7UVQ
	2A2	D4	1.9	Roeffen[51] , De Jong[40]	**9N5H, 9N89**
	18F25	D7	10 to 30 *	Roeffen[51], Inklaar[38]	**9N5O, 9N6U**
Pfs48/45	RUPA-58	D1-i	7.5	Kucharska[17]	8U1P
	RUPA-94	D1-i	<100 *	Fabra-García[34]	-
	RUPA-46	D1-ii	2 to 10 *	Fabra-García[34]	-
	RUPA-71	D1-ii	2 to 10 *	Fabra-García[34]	**9N5I, 9N7K**
	RUPA-154	D2	103.1	Kucharska[17]	8U1P
	RUPA-160	D2	± 10 *	Fabra-García[34]	-
	10D8	D2	>350 *	Ko[16]	7ZXF/7ZXG/7ZWM
	TB31F	D3 - 1a	0.86	Kundu[43], Kucharska[17], Ko[16]	6E63/7ZXF/6H5N
	RUPA-29	D3 - 1a	0.4 to 2 *	Fabra-García[34]	7UXL
	RUPA-50	D3 - 1a	0.4 to 2 *	Fabra-García[34]	-
	RUPA-47	D3 - 1a	10 to 100 *	Fabra-García[34]	7UNB
	RUPA-57	D3 - 1a	>100**	Fabra-García[34]	-
	RUPA-72	D3 - 1a	>100**	Fabra-García[34]	-
	RUPA-89	D3 - 1a	>100**	Fabra-García[34]	-
	32F3	D3 - 1ab	<25 *	Vermeulen[27], Ko[16]	7ZWI/7ZWF/7ZWM
	RUPA-61	D3 - 1b	± 10 *	Fabra-García[34]	-
	RUPA-44	D3 - 1b	11	Fabra-García[34]; Kucharska[17]	7UXL / **9N5I**
	RUPA-117	D3 - 1b	± 10 *	Fabra-García[34]	7UNB
Negative controls	PGDM1400	HIV Env.	n/a	n/a	n/a
	CIS43	PfCSP	n/a	n/a	n/a

bioRxiv preprint doi: https://doi.org/10.1101/2025.02.14.638310; this version posted February 15, 2025. The copyright holder for this preprint (which was not certified by peer review) is the author/funder, who has granted bioRxiv a license to display the preprint in perpetuity. It is made available under aCC-BY 4.0 International license.

Table S2: Pfs230 – Pfs48/45 residue contact table. Related to **Figure 3**.

Pfs48/45 Domain	Pfs48/45 Residue	BSA (Å^2)	Pfs230 Residue contacts
Domain 1	ASN38	18.0	3108
	GLU40	25.2	3011, 3013
	ILE41	59.9	3011, 3112, 3013
	SER 42	87.6	2986, 2997, 2998, 3011,3012
	GLY43	33.5	2997
	PHE44	15.0	2997
	ILE45	85.7	2984, 3108-3110
	GLY46	15.8	3110
	TYR47	65.2	3110-3114
	LYS48	49.4	2982, 2997, 3114
	ASN50	16.6	2993
	GLU54	47.5	2990, 2993, 3001
	GLY55	9.4	2993
	VAL56	90.1	2942, 2991, 2993, 2994
	HIS57	78.1	2942, 3116, 3118
	GLU65	34.2	2943, 3118
	ARG67	30.1	3118
	SER68	20.4	3117-3118
	ILE69	24.9	3116-3118
	PHE70	55.7	3115-3117
	CYS71	24.5	3114-3116
	THR72	49.9	3113-3115
	ILE73	15.8	3114
	HIS74	24.1	3115
	SER75	8.1	3112
	ASP80	16.6	3110
	ASN136	38.9	3001
	TYR137	17.5	2999
Domain 2	ASN247	13.0	3111
	LYS248	70.9	3109-3111
	ILE249	10.7	3108
	ILE250	17.4	3108
Domain 3	ILE348	27.8	2839
	ILE349	11.3	2839
	PHE354	48.1	2871, 2980
	GLN355	24.8	2870
	ILE369	53.2	2868, 2870, 2977
	VAL370	8.5	2977
	TYR371	10.9	2870, 2871, 2978-2980
	ASP373	46.6	2980, 3113
	ASN377	31.1	3115
	GLY379	25.3	3111, 3112
	ASP380	25.8	3111, 3112
	GLU385	12.4	3079

bioRxiv preprint doi: https://doi.org/10.1101/2025.02.14.638310; this version posted February 15, 2025. The copyright holder for this preprint (which was not certified by peer review) is the author/funder, who has granted bioRxiv a license to display the preprint in perpetuity. It is made available under aCC-BY 4.0 International license.

Table S3. Data collection and refinement statistics for the RUPA-71, 2A2, and 18F25 Fab crystal structures. Related to **Figures 4-5.**

	RUPA-71 Fab	2A2 Fab	18F25 Fab
Wavelength (Å)	0.95357	0.920194	0.920194
Space group	$P\ 2_1\ 2_1\ 2_1$	$P\ 2_1\ 2_1\ 2_1$	$P\ 2_1\ 2_1\ 2_1$
Cell dimensions			
a, b, c (Å)	64.66, 74.55, 204.9	59.97, 76.68, 91.87	37.64, 81.80, 140.53
α, β, γ (°)	90, 90, 90	90, 90, 90	90, 90, 90
Resolution (Å)	48.85 - 2.28 (2.36 –2.28)	28.51 - 1.20 (1.21 -1.20)	34.19 - 1.60 (1.63 - 1.60)
No. molecules in ASU	2	1	1
No. unique observations	46,105 (4434)	254,175 (8275)	109,994 (5231)
Multiplicity	15.4 (15.2)	3.5 (3.6)	3.3 (3.3)
R_{meas}[1] (%)	39.3 (229.3)	8.1 (68.5)	86.2 (74.4)
R_{pim}[2] (%)	13.7 (80.7)	4.2 (35.6)	4.6 (40.2)
<I/sigma I>	7.5 (1.7)	8.9 (2.1)	9.8 (1.8)
$CC_{1/2}$ (%)	99.1 (56.1)	99.7 (71.6)	99.8 (55.6)
Completeness (%)	100 (100)	99.5 (98.2)	99.4 (98.6)
Refinement Statistics			
Non-hydrogen atoms	7037	3708	4056
Macromolecule	6776	3343	3432
Heteroatom	52	0	20
Water	209	427	604
R_{work}[3]/ R_{free}[4] (%)	19.1/ 24.1	18.6 / 19.4	18.8 / 21.3
Rms deviations from ideality			
Bond lengths (Å)	0.009	0.004	0.006
Bond angle (°)	0.99	0.78	0.83
Ramachandran plot			
Favoured regions (%)	98.0	98.3	97.4
Allowed regions (%)	2.0	1.7	2.6
B-factors (Å^2)			
Wilson B-factor	36.5	9.8	15.2
Average B-factors	41.4	12.8	17.1
PDB code	9N7K	9N89	9N6U

*Values in parentheses are for the highest-resolution shell.

[1]$R_{meas} = \Sigma hkl\ [N/(N-1)]1/2\ \Sigma i\ |\ Ihkl, i - <Ihkl>|\ /\ \Sigma hkl <Ihkl>$.

[2]$R_{pim} = \Sigma hkl\ [1/(N-1)]1/2\ \Sigma i\ |\ Ihkl, i - <Ihkl>|\ /\ \Sigma hkl <Ihkl>$.

[3]$R_{work} = \Sigma||F_o| - |F_c||/\Sigma|F_o|$.

[4]R_{free} is calculated for 5% of the data that was not included in refinement.

bioRxiv preprint doi: https://doi.org/10.1101/2025.02.14.638310; this version posted February 15, 2025. The copyright holder for this preprint (which was not certified by peer review) is the author/funder, who has granted bioRxiv a license to display the preprint in perpetuity. It is made available under aCC-BY 4.0 International license.

Table S4: 2A2, 18F25, and RUPA-71 contacts table. Related to **Figures 4 and 5**.

Pfs230 Residue	Structural Element	BSA (Å²)	2A2 HC Residue	Structural Element	BSA (Å²)	2A2 KC Residue	Structural Element	BSA (Å²)
HIS1159	D4: β2-β3 loop	37.1	ASN33	HCDR1	16.0	SER1	N-term / FR1-IMGT	27.1
GLU1160	D4: β3	13.6	ASN35	FR2-IMGT	2.3	ILE2	N-term / FR1-IMGT	7.9
TYR1194	D4: β5	27.2	TRP47	FR2-IMGT	21.6	GLN27	KCDR1	72.7
GLN1196	D4: β5	67.5	ASN50	FR2-IMGT	19.2	SER28	KCDR1	8.2
GLU1198	D4: β5-β6 loop	20.1	ASP52	HCDR2	21.4	ASN30	KCDR1	6.2
TYR1205	D4: β6	33.6	HIS55	HCDR2	57.7	ASP91	KCDR3	8.1
LYS1206	D4: β6-β7 loop	51.1	GLY57	HCDR2	23.1	TYR92	KCDR3	80.0
GLY1207	D4: β6-β7 loop	21.4	THR58	HCDR2	11.2	SER93	KCDR3	41.9
LEU1208	D4: β6-β7 loop	169.6	THR59	FR3-IMGT	60.3	SER94	KCDR3	74.0
ASN1209	D4: β6-β7 loop	107.2	TYR60	FR3-IMGT	7.9	PRO95	KCDR3	12.4
SER1210	D4: β7	53.5	ASN61	FR3-IMGT	1.0	LEU96	KCDR3	26.3
VAL1211	D4: β7	43.9	GLN62	FR3-IMGT	51.2			
ASP1212	D4: β7	0.4	LYS65	FR3-IMGT	14.3			
THR1215	D4: α1	31.4	SER99	HCDR3	0.1			
GLN1250	D4: β10	32.6	THR100	HCDR3	0.9			
THR1252	D4: β10	8.9	LEU101	HCDR3	19.9			
GLN1255	D4: β10-β11	4.3	TYR102	HCDR3	69.5			
VAL1256	D4: β11	8.0	GLY103	HCDR3	16.5			
VAL1257	D4: β11	69.7	SER105	HCDR3	32.1			
LYS1259	D4: β11	40.9						
LYS1261	D4: β11	15.1						

Pfs230 Residue	Structural Element	BSA (Å²)	18F25 HC Residue	Structural Element	BSA (Å²)	18F25 KC Residue	Structural Element	BSA (Å²)
LYS1751	D7: β4-β5 loop (D7-ID)	35.7	TYR32	HCDR1	33.3	THR30	KCDR1	15.8
VAL1752	D7: β4-β5 loop (D7-ID)	46.5	TRP33	HCDR1	10.2	ASN31	KCDR1	10.0
ILE1754	D7: β4-β5 loop (D7-ID)	26.6	TYR52	HCDR2	23.3	ASP32	KCDR1	32.2
GLU1756	D7: β4-β5 loop (D7-ID)	35.4	ASP54	HCDR2	8.8	TYR49	FR2-IMGT	24.3
VAL1801	D7: β4-β5 loop (D7-ID)	19.7	ASP56	HCDR2	12.1	SER50	KCDR2	3.6
LEU1802	D7: β4-β5 loop (D7-ID)	79.2	ARG58	HCDR2	10.5	TYR53	KCDR2	32.0
ASP1803	D7: β4-β5 loop (D7-ID)	52.0	ARG94	HCDR3	1.5	TYR55	FR3-IMGT	7.2
ASN1804	D7: β4-β5 loop (D7-ID)	6.7	LEU96	HCDR3	27.3	HIS91	KCDR3	27.5
THR1806	D7: β4-β5 loop (D7-ID)	31.6	TYR97	HCDR3	42.3	TYR92	KCDR3	84.9
PHE1807	D7: β4-β5 loop (D7-ID)	114.5	LEU98	HCDR3	69.8	SER93	KCDR3	1.3
GLU1808	D7: β4-β5 loop (D7-ID)	40.0						
LYS1809	D7: β4-β5 loop (D7-ID)	1.8						
LYS1856	D7: β8-β9 loop	24.8						
ASP1857	D7: β8-β9 loop	0.6						

Pfs48/45 Residue	Structural Element	BSA (Å²)	RUPA-71 HC Res.	Structural Element	BSA (Å²)	RUPA-71 KC Res.	Structural Element	BSA (Å²)
LYS33	D1: N-term	28.2	GLU2	N-term / FR1-IMGT	47.1	LYS32	KCDR1	40.3
SER35	D1: N-term	13.8	GLY26	HCDR1	19.3	LEU46	FR2-IMGT	20.0
SER36	D1: N-term	10.97	PHE27	HCDR1	14.0	TYR49	FR2-IMGT	56.8

bioRxiv preprint doi: https://doi.org/10.1101/2025.02.14.638310; this version posted February 15, 2025. The copyright holder for this preprint (which was not certified by peer review) is the author/funder, who has granted bioRxiv a license to display the preprint in perpetuity. It is made available under aCC-BY 4.0 International license.

LYS88	D1: β3-β4 loop	235.1	THR28	HCDR1	26.0	THR56	FR3-IMGT	78.8
LYS89	D1: β3-β4 loop	40.3	ASP31	HCDR1	24.4	SER67	FR3-IMGT	39.2
SER90	D1: β3-β4 loop	45.4	TYR32	HCDR1	24.7			
LYS101	D1: β4-β5 loop	81.1	ASP52C	HCDR2	48.3			
GLN104	D1: β4-β5 loop	61.0	GLU53	HCDR2	17.5			
SER119	D1: α1	20.8	ARG94	HCDR3	13.6			
TYR126	D1: α1-β7 loop	47.0	GLY96	HCDR3	23.8			
GLU127	D1: β7	41.9	LEU97	HCDR3	60.2			
ILE128	D1: β7	41.9	ARG98	HCDR3	86.1			
GLU129	D1: β7	61.1	TRP99	HCDR3	58.6			
GLU130	D1: β7	76.2	TYR100	HCDR3	96.1			
ASN131	D1: β7	51.4	ASP100A	HCDR3	35.2			
ASP132	D1: β7-β8 loop	71.4	SER100B	HCDR3	68.9			
THR133	D1: β7-β8 loop	113.1	ASP101	HCDR3	29.5			
ASN134	D1: β7-β8 loop	24.3						
PRO135	D1: β7-β8 loop	25.5						
ARG140	D1: β8	5.7						

bioRxiv preprint doi: https://doi.org/10.1101/2025.02.14.638310; this version posted February 15, 2025. The copyright holder for this preprint (which was not certified by peer review) is the author/funder, who has granted bioRxiv a license to display the preprint in perpetuity. It is made available under aCC-BY 4.0 International license.

Table S5: Oligonucleotides used in this study. Red or green nucleotides depict restriction sites used for cloning. Underlined nucleotides were used for overlap-PCR. Blue nucleotides highlight the CRISPR/Cas9 guide RNA sequence. Orange nucleotides are three sequential guanosine nucleotides used for template switching, as described in (76). Related to **Methods.**

Name	Function	Sequence
**HDR template - primers**		
p1	5'HR(-A) Pfs230-tag FW	cagttCGTCTCtggagCCACCATATGTACATAAAGATATACATTTCTCATTAG
p2	5'HR-A Pfs230-tag RV	aGCcCTactcTGAGGTTCTGGGATTATATAATTAGGATCAAATGTG
p3	5'HR Pfs230-tag RV	gtcaaCGTCTCataccAAGCTTTCTCAAGTATTTTG
p4	3'HR Pfs230-tag FW	cagttCGTCTCtgcacTCGTAAATAATTAATCAAACATATATATAATCAAAAGG
p5	3'HR Pfs230-tag RV	cagttCGTCTCtatggGGACTCTAAGATATTTCTTTCGCTAATCC
p6	H2B_mScarlet_biUTR-A FW	cagttGGTCTCTggagCGTCTCttaagCTAAATGGATGATTCCCCTCTTTGCAATATG
p7	H2B_mScarlet_biUTR-A RV	CAGTTggtctctTGACatttcatatttgcatgtgcatattaaaagtatcg
p8	H2B_mScarlet_biUTR-B FW	cagttggtctctGTCAgtctctgcataattttcgctatttaaatatat
p9	H2B_mScarlet_biUTR-B RV	cagttGGTCTCTatggCGTCTCtGTGCgTTCGAATCAACTCCGTTCAGTTAAAAATATTAC
p10	5'HR Pfs230ΔD13-14 FW	CAGTcgtctcTagagGGGATGATGTACATTTATTTTATCCTCCTC
p11	5'HR Pfs230ΔD13-14 RV	CAGTcgtctcTatccaccTGTTTTTTTTTCATCTATTTTTATTTTATCCATTGTAC
p12	5'HR Pfs230ΔCterm FW	CAGTcgtctcTagagCAATTGGTAAAGATATATGTAAATATGATGTTACTAC
p13	5'HR Pfs230ΔCterm RV	CAGTcgtctcTatccaccATTAGGATCAAATGTGATTCGTATAGTATAATTTG
p4	3'HR Pfs230Δ FW	See p4
p14	3'HR Pfs230Δ RV	cagtCGTCTCtcggtGGACTCTAAGATATTTCTTTCGCTAATCC
p15	GAPDH_swap FW	cagtGCTAGCGAAAAGAATTAAAAAGCCGAAGAAAAAATATATG
p16	GAPDH_swap RV	cagtGAGCTCGAGCTATGAAAAACATGGGTGTG
p17	C-terminal 3xFLAG FW	cagtGAATTcgtctcTggatCTGGTGATTACAAAGATCATGATGG
p18	C-terminal 3xFLAG RV	cagtAAGCTTcgtctcTcttaGTCATGATCCTTGTAATCTATATCGTG
**HDR template - oligonucleotides**		
p19	5'HR-B anneal Pfs230 FW	CTAATTATATAATCCCAGAACCTCAgagtAGgGCtATCATTAAATATGTAGATCTGCA AGATAAAAATTTTGCAAAATACTTGAGAAAGCTTggtaTGAGACGttgac
p20	5'HR-B anneal Pfs230 RV	gtcaaCGTCTCataccAAGCTTTCTCAAGTATTTTGCAAAATTTTTATCTTGCAGATCTA CATATTTAATGATaGCcCTactcTGAGGTTCTGGGATTATATAATTAG
p21	FLAG-C-tag FW	gatccgtctctGGTACATCTGGTGATTACAAAGATCATGATGGAGATTATAAAGATCAC GATATAGATTACAAGGATCATGACGGTTCAGGTGAACCAGAAGCATAAgtGAGACG
p22	FLAG-C-tag RV	aattCGTCTCacTTATGCTTCTGGTTCACCTGAACCGTCATGATCCTTGTAATCTATA TCGTGATCTTTATAATCTCCATCATGATCTTTGTAATCACCAGATGTACCagagacg
**CRISPR/Cas9 guide plasmid oligonucleotides**		
p23	Guide Pfs230 Cterm FW	tattgTTAATGATGGCTCTTGATTG
p24	Guide Pfs230 Cterm RV	aaacCAATCAAGAGCCATCATTAAc
p25	Guide Pfs230 D13 FW	tattgATTCTATTACATTATCAAGA
p26	Guide Pfs230 D13 RV	aaacTCTTGATAATGTAATAGAATc
**Template-switch oligonucleotide for hybridoma sequencing**		
p27	RT-PCR template switch FW	*AAGCAGTGGTATCAACGCAGAGTACATrGrGrG*
p28	RT-PCR for mIgK RV	TTGTCGTTCACTGCCATCAATC
p29	RT-PCR for mIgH RV	AGCTGGGAAGGTGTGCACAC
p30	Universal ISPCR primer FW	AAGCAGTGGTATCAACGCAGAG

bioRxiv preprint doi: https://doi.org/10.1101/2025.02.14.638310; this version posted February 15, 2025. The copyright holder for this preprint (which was not certified by peer review) is the author/funder, who has granted bioRxiv a license to display the preprint in perpetuity. It is made available under aCC-BY 4.0 International license.

p31	mIgK RV	ACATTGATGTCTTTGGGGTAGAAG
p32	mIgH RV	GGGATCCAGAGTTCCAGGTC

bioRxiv preprint doi: https://doi.org/10.1101/2025.02.14.638310; this version posted February 15, 2025. The copyright holder for this preprint (which was not certified by peer review) is the author/funder, who has granted bioRxiv a license to display the preprint in perpetuity. It is made available under aCC-BY 4.0 International license.

Table S6: Amino acid sequence of variable chains of mAbs 18F25 and 2A2. Related to **Methods.**

mAb	Region	Amino acid sequence
18F25	Heavy chain (VDJ)	QVQLQQSGAELARPGASVKLSCKASGYTFTNYWMQWVKQRPGQGLEWFGAIYPGDGD TRYTQKFKGKATLTADKSSSTAYMQLSSLASEDSAVYYCARSLYLGAMDYWGQGTSVTVSS
	Light chain (VJ)	DIVMTQSHKFMSTSVGDRVSITCKASQDVTNDVAWYQQKPGQSPKLLIYSAS YRYTGVPDRFTGSGSGTDFIFTISSVQAEDLAVYYCQQHYSAPPTFGGGTKLEIK
2A2	Heavy chain (VDJ)	EVQLQQSGPELEKPGASVKISCKASGYSITDYNMNWVKLSNGKSLEWIGNIDPSHGGTTY NQKFKGKATLTVDKSSSTAYMQLKSLTSEDSAVYHCARSTLYGNSAMDCWGQGTSVTVSS
	Light chain (VJ)	SIVMTQTPKFLLVPAGDRVTITCKASQSVNNDVTWYQQKPGQSPKLLIYYASN RYTGVPDRFTGSGYGTDFTFTISTVQAEDLAVYFCQQDYSSPLTFGAGTKLEL
